# Supplementary material for: Birds have peramorphic skulls, too: anatomical network analyses reveal oppositional heterochronies in avian skull evolution
Source: Commun Biol. 2020 Apr 24;3:195. doi: 10.1038/s42003-020-0914-4 (PMC7181600; doi:10.1038/s42003-020-0914-4)

Supplementary information

Birds have peramorphic skulls, too: Anatomical Network Analyses reveal oppositional heterochronies in avian skull evolution

Content

1. Robustness of the results
2. Anatomical Network Analysis (*AnNA*) vs. *EMMLi* analysis
3. Supplementary figures
4. References
5. Skull network analysis results

1. Robustness of the results

Skull modularity of Tyrannosauridae. For the most part, the connectivity modularity pattern of the adult *Tarbosaurus* resembles that of *Tyrannosaurus* from the Werneburg et al.[1] study (Supplementary Fig. 1). Both species share the same number of modules. However, the assignment of some bones is not identical. While in *Tyrannosaurus*, for instance, the nasals are part of the skull roof module that includes also the lacrimal, prefrontal, frontal and postorbital, they belong to the preorbital module in *Tarbosaurus*. As we used a different cluster method (*Ward.2D* instead of *UPGMA*) and treated the fused elements like the nasals, frontals, parietals and vomers as one unit, these discrepancies are probably related to differences in the methodology.

Impact of phylogeny. The repetition of the *pFDA* and ancestral state reconstruction analyses using the crown-group topology of Ericson et al.[2] does not change the general outcome of the results. As for the Hackett et al.[3] topology, non-avian theropods cluster closer with juvenile than adult birds (Supplementary data 2 file). Mapping *PCo1* and *PCo2* onto both trees, the substitution of adult crown-group species with the juvenile specimens is more parsimonious for both topologies (using the standard deviation as a proxy for parsimony). This effect becomes even clearer after *PCo* values are standardized for early juvenile size (i.e., residual values; Supplementary data 2 file). This signal retains after reshuffling the terminal taxa 1,000 times across the topology. This is also true for *PCo1* when the log-transformed product of ancestral values is used as a proxy for parsimony. In contrast, for *PCo2* the signal is only present between adults and juveniles standardized for size (Supplementary Fig. 4). Nevertheless, we consider the heterochronic signal to be robust with respect to the phylogenetic relationships.

Impact of network parameters on PCoA. In a recent study Esteve-Altava et al.[4] applied multidimensional scaling in form of principal component analysis (*PCA*), using a different set of the network parameters, including nodes (*N*), links (*K*), density (*D*), clustering coefficient (*C*), path length (*L*), heterogeneity (*H*), assortativity (*A*), and parcellation (*P*). To test, if the selection of network parameters affects the outcome of the *PCoA*, we applied different combinations of parameters and compared the distribution of the resulting *PCo1* and *PCo2* values with the original ones (Supplementary Fig. 5). In all cases, ordinary least square linear regression analyses indicate a highly significant correlation between the outcome of the original and alternative *PCoA*'s. This is even the case for *PCo1*, if only the two parameters *N* and *K* are selected (Supplementary data 2 file). These comparisons indicate that the outcome of our original analysis seems to be robust in terms of the selection of network parameters.

Impact of module asymmetry. As reported in the main text, many species in our dataset have a left-right asymmetry in terms of module distribution. This asymmetry might be partly explainable with the methodology, in which modularity is computed in the basis of cluster analysis with dichotomous branching[1]. To test this, we applied three alternative network analyses for the basal avialan *Archaeopteryx lithographica*: 1) skull configuration including only paired bones, 2) skull configuration including only paired bones from the left side, and 3) skull configuration including only paired bones from the left side and unpaired bones (see Supplementary Table S1). The deletion of unpaired bones, results in a fully symmetric modularity between the left and right skull side, in which *A. lithographica* possesses left and right snout modules including the zygomatic arches and majority of palatal bones, left and right skull roof/temporal modules and left and right mandible modules. This pattern is more or less similar to the module distribution of the left skull side in the original analysis (Supplementary Fig. 6). Analyzing only one skull side reveals quite similar module distributions, although here the zygomatic arch and palatal bones form an own module (Supplementary Fig. 7). Based on these comparisons, we conclude that module asymmetry is at least partly driven by the presence of unpaired elements, but the phenomenon has to be tested in more detail in the future. However, as the deletion of the *S*-modules and *Q*-modules and the corresponding Q_{max} parameter from the *PCoA* did not change the general outcome of the analysis, module asymmetry seems not to affect our macroevolutionary findings.

2. Anatomical Network Analysis (*AnNA*) vs. *EMMLi* analysis

While *AnNA* estimates the number of modules based on the number of bones and their connections to each other (see connectivity modularity)[5], *EMMLi* based on geometric morphometric shape data (see variational modularity)[6]. For adult bird skulls, both analyses produced a different number of modules, in which *EMMLi* produces a higher number of

modules than *AnNA*[1,7,8]. This discrepancy is obviously a result of the different methodological approaches, measuring different types of modularity. However, as indicated by our study, juvenile birds actually possess a higher number of modules than adults, which fall in the range of the *EMMLi* approach. Thus, it could be possible that the latter approach is just less sensitive to ontogenetic changes that include a reduction of modules due to bone fusion. In this case, *EMMLi* reproduces the juvenile condition with a high module number as the best solution for geometric morphometric shape data. How this outcome is dependent on the respective (semi-)landmark concept and the superimposition method has to be tested in the future[9]. Nevertheless, both methodologies provide congruent results, as non-avian theropods have a higher cranial modularity than crown group birds[1,7,8].

3. Supplementary figures

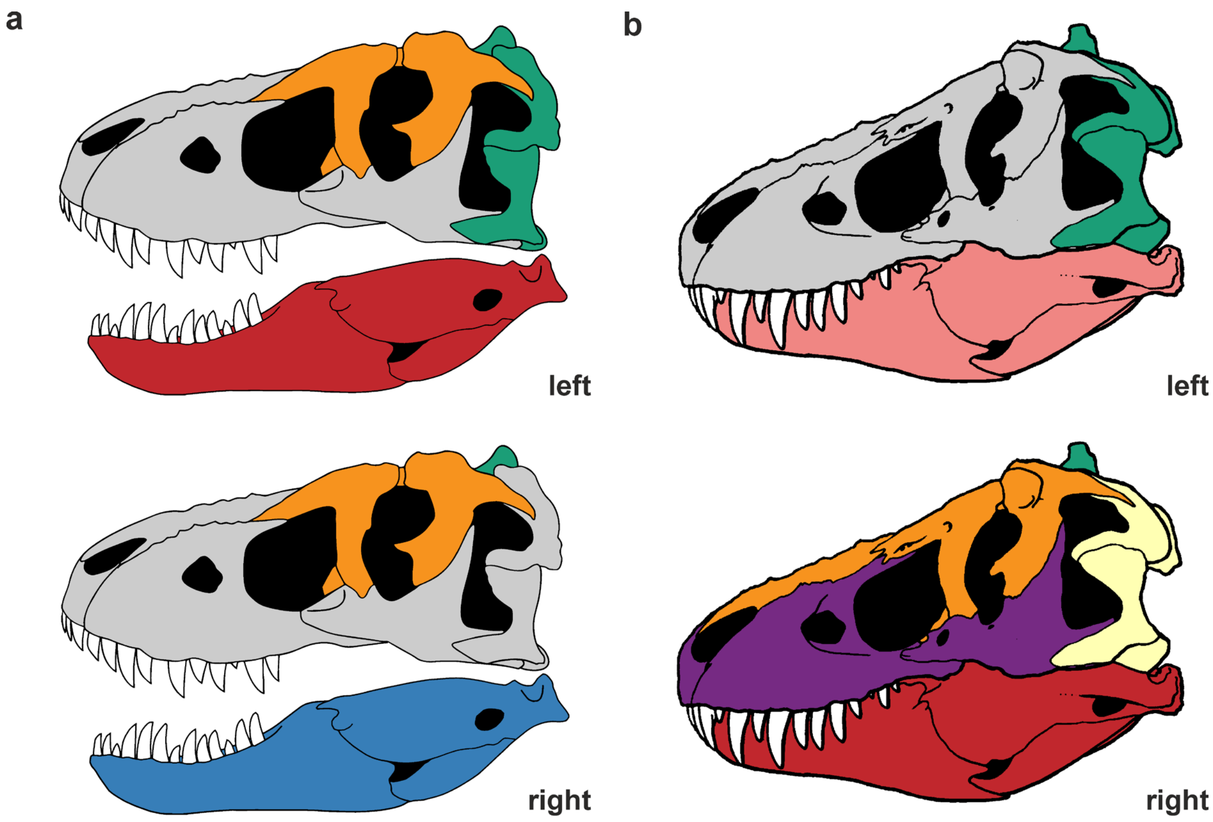

Supplementary Figure 1 | Comparison of skull modularity in large bodied tyrannosaurids. a, *Tarbosaurus bataar* (modified after Hurum & Sabath[10]) using *Ward.2D* cluster (this study). **b,** *Tyrannosaurus rex* (modified after Carr[11]) using *UPGMA* cluster[1].

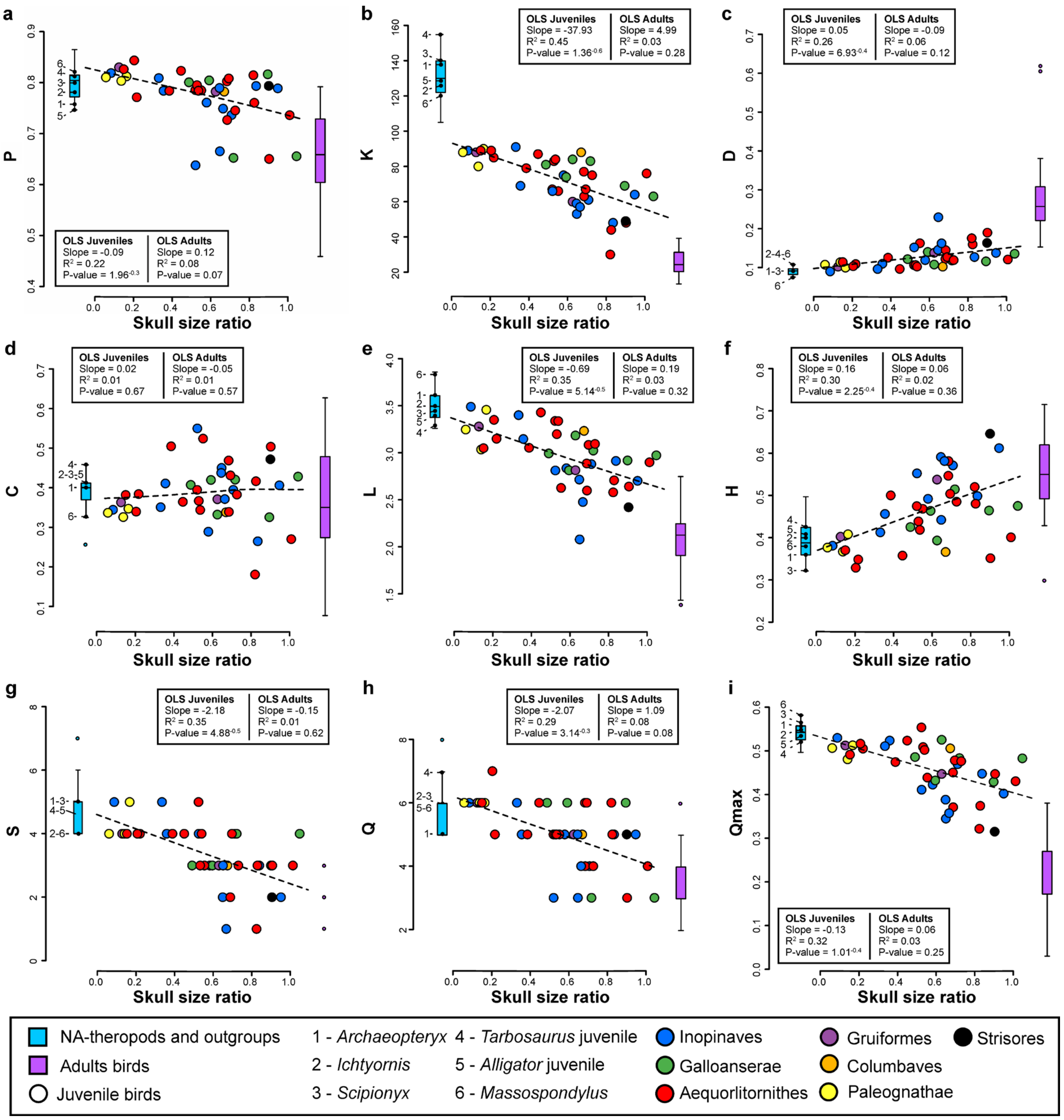

Supplementary Figure 2 | Range of number of network parameters in non-avian archosaurs, juvenile and adult birds. a, Parcellation (*P*). **b**, Number of connections (*K*). **c**, Density of connections (*D*). **d**, Mean clustering coefficient (*C*). **e**, Mean shorted path length (*L*). **f**, Variance of connectivity (*H*). **g**, Number of *S*-modules. **h**, Number of *Q*-modules. **i**, The strength of modular organization is expressed by the parameter Q_{max}. For juvenile birds, the range of network parameters is plotted against relative skull size (ratio of skull box volume). Results of ordinary least square regression analysis (OLS) describing the correlation

between network parameters and relative skull size of juvenile and adult birds are given in the box. The plot for the number of nodes (*N*) is given in Fig. 2 of the main text.

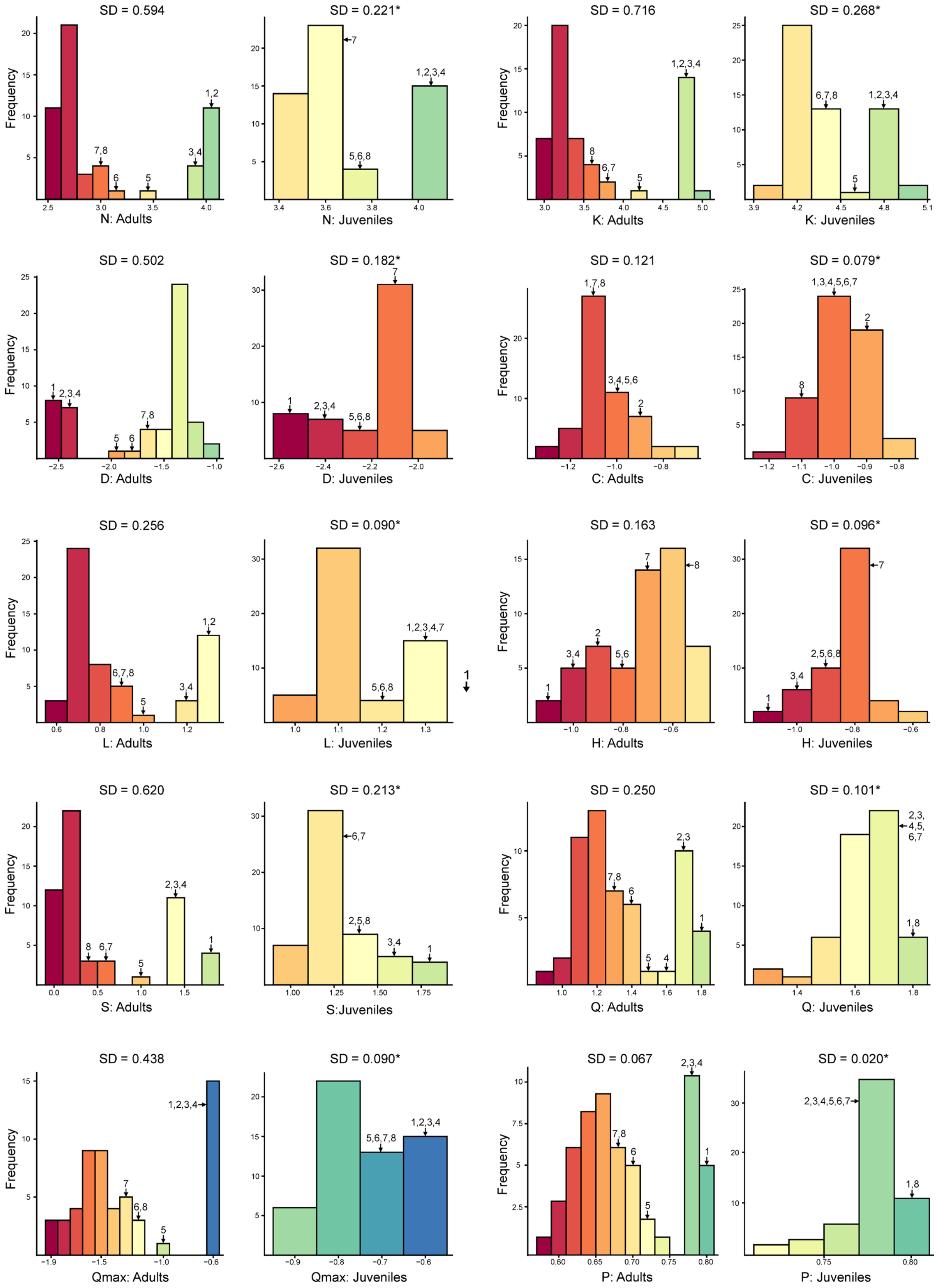
SD = 0.594
N: Adults
SD = 0.221*
N: Juveniles
SD = 0.716
K: Adults
SD = 0.268*
K: Juveniles
SD = 0.502
D: Adults
SD = 0.182*
D: Juveniles
SD = 0.121
C: Adults
SD = 0.079*
C: Juveniles
SD = 0.256
L: Adults
SD = 0.090*
L: Juveniles
SD = 0.163
H: Adults
SD = 0.096*
H: Juveniles
SD = 0.620
S: Adults
SD = 0.213*
S:Juveniles
SD = 0.250
Q: Adults
SD = 0.101*
Q: Juveniles
SD = 0.438
Qmax: Adults
SD = 0.090*
Qmax: Juveniles
SD = 0.067
P: Adults
SD = 0.020*
P: Juveniles
Frequency

Supplementary Figure 3 | Histograms summarizing the distribution of ancestral values all network parameters and *PCo1* for adult and juvenile crown birds (original values). For all parameters, the hypothetical substitution of adult crown birds by their juvenile counterparts leads to a more parsimonious evolution as indicated by the standard deviation *SD* (*). (1) Theropoda; (2) Coelurosauria; (3) Eumaniraptora; (4) Avialae; (5) Ornithurae; (6) Aves; (7) Neognathae; (8) Palaeognathae.

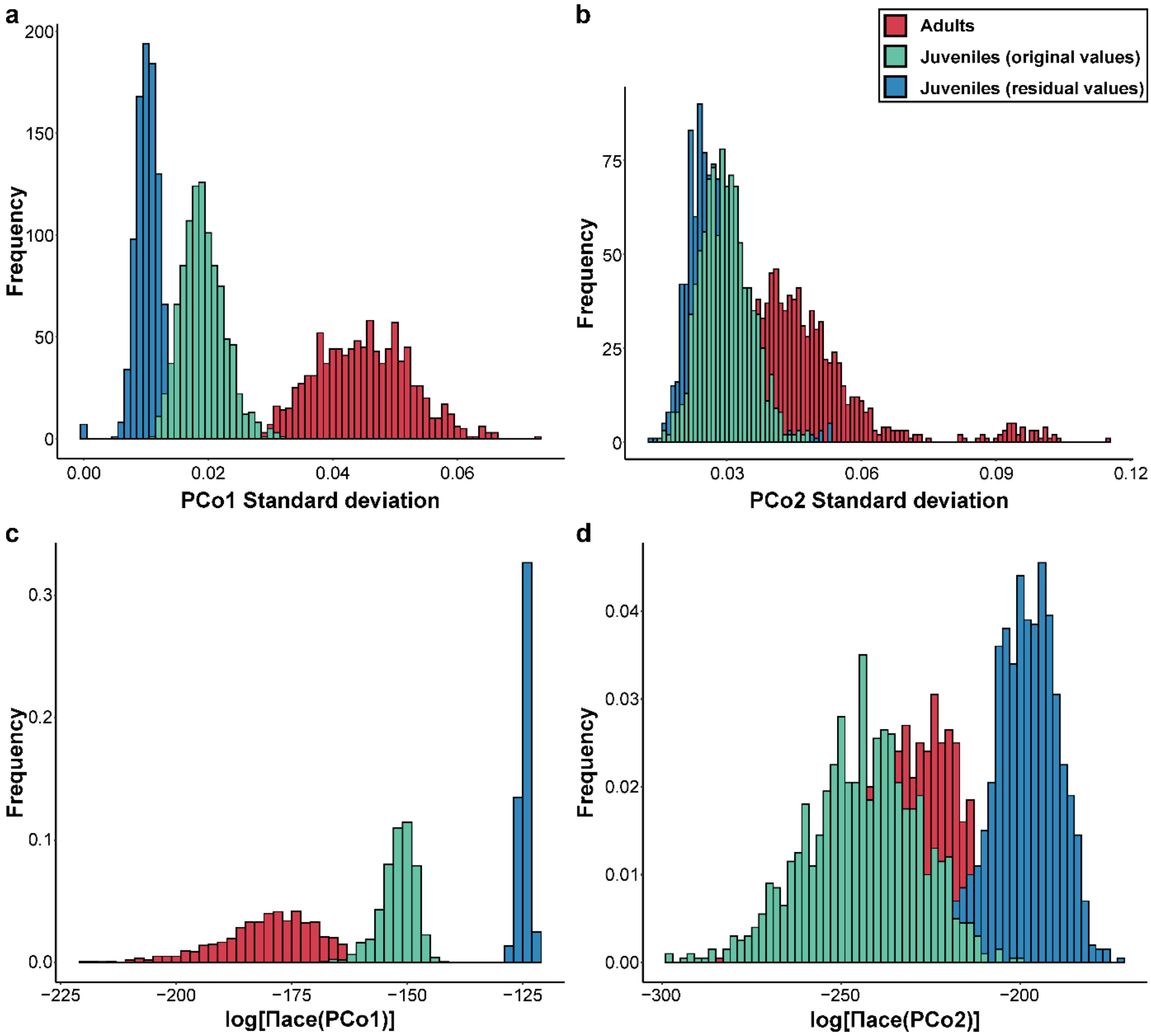

Supplementary Figure 4 | Histograms of ancestral values of *PCo1* and *PCo2* after permutation over 1,000 trees. a, Standard deviation of ancestral values of *PCo1*. **b**, Standard deviation of ancestral values of *PCo2*, **c**, Log-transformed product of ancestral values of *PCo1*. **d**, Log-transformed product of ancestral values of *PCo2*.

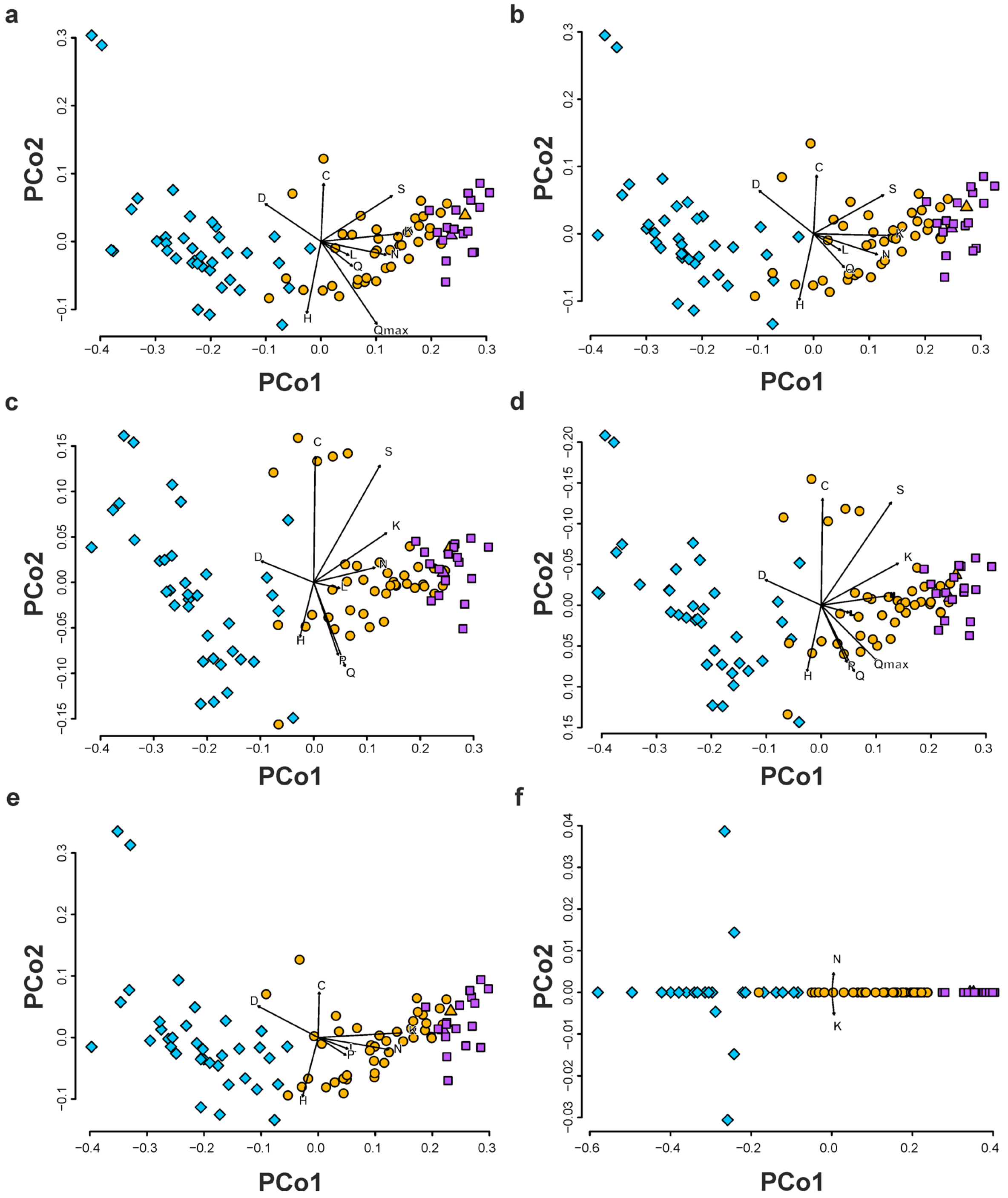

Supplementary Figure 5 | Principal coordinate analyses based on different network parameters. a, original *PCoA*. **b**, *PCoA* without modular partition Q_{max}. **c**, *PCoA* with parcellation *P* and without Q_{max}. **d**, *PCoA* with *P* and Q_{max}. **e**, *PCoA* with *P* and without *S*-modules, *Q*-modules and Q_{max}. **f**, *PCoA* just based on number of bones *N* and bone contacts *K*.

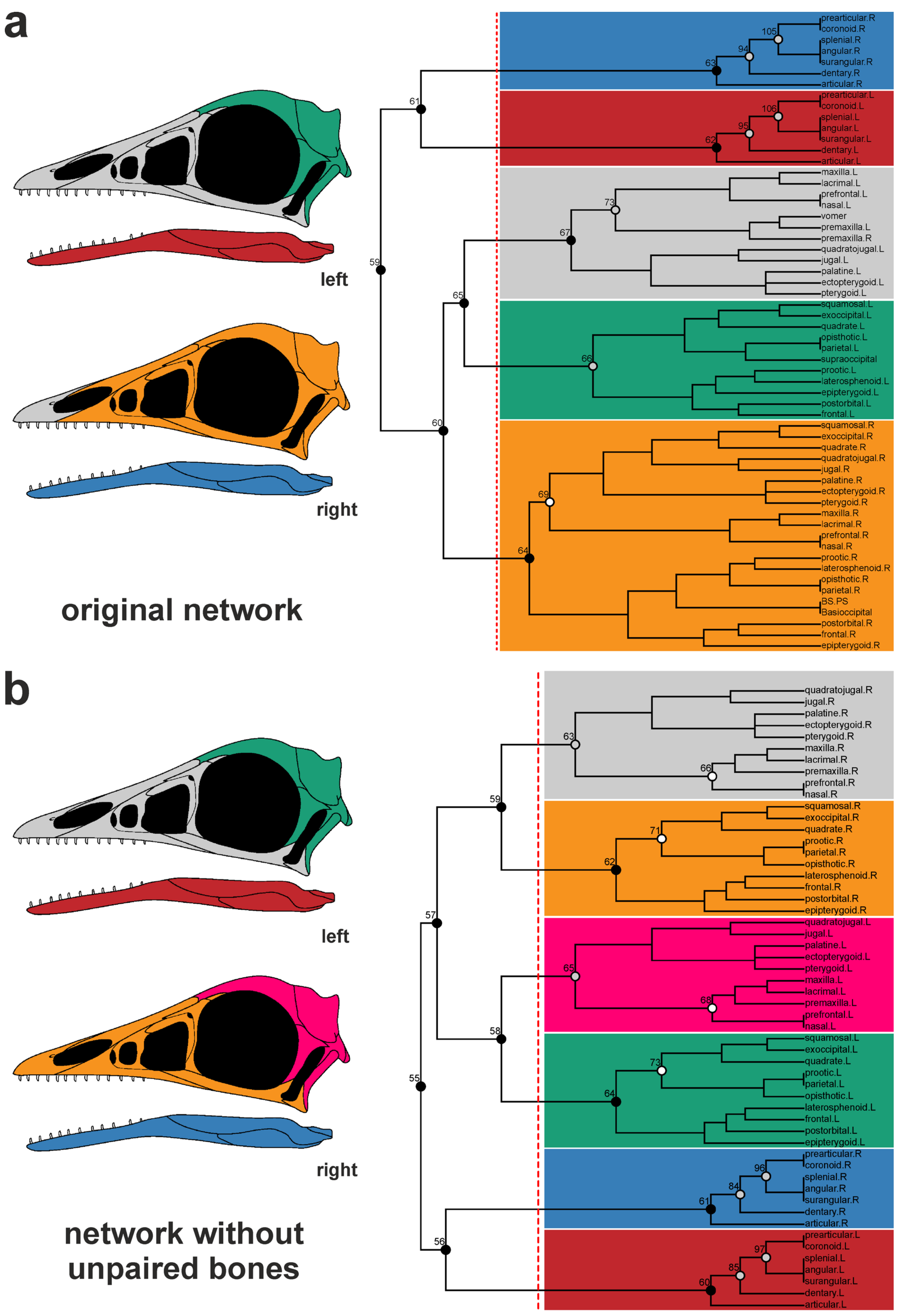
a
left
right
original network
prearticular.R
coronoid.R
splenial.R
angular.R
surangular.R
dentary.R
articular.R
prearticular.L
coronoid.L
splenial.L
angular.L
surangular.L
dentary.L
articular.L
maxilla.L
lacrimal.L
prefrontal.L
nasal.L
vomer
premaxilla.L
premaxilla.R
quadratojugal.L
jugal.L
palatine.L
ectopterygoid.L
pterygoid.L
squamosal.L
exoccipital.L
quadrate.L
opisthotic.L
parietal.L
supraoccipital
prootic.L
laterosphenoid.L
epipterygoid.L
postorbital.L
frontal.L
squamosal.R
exoccipital.R
quadrate.R
quadratojugal.R
jugal.R
palatine.R
ectopterygoid.R
pterygoid.R
maxilla.R
lacrimal.R
prefrontal.R
nasal.R
prootic.R
laterosphenoid.R
opisthotic.R
parietal.R
BS.PS
Basioccipital
postorbital.R
frontal.R
epipterygoid.R
b
left
right
network without
unpaired bones
quadratojugal.R
jugal.R
palatine.R
ectopterygoid.R
pterygoid.R
maxilla.R
lacrimal.R
premaxilla.R
prefrontal.R
nasal.R
squamosal.R
exoccipital.R
quadrate.R
prootic.R
parietal.R
opisthotic.R
laterosphenoid.R
frontal.R
postorbital.R
epipterygoid.R
quadratojugal.L
jugal.L
palatine.L
ectopterygoid.L
pterygoid.L
maxilla.L
lacrimal.L
premaxilla.L
prefrontal.L
nasal.L
squamosal.L
exoccipital.L
quadrate.L
prootic.L
parietal.L
opisthotic.L
laterosphenoid.L
frontal.L
postorbital.L
epipterygoid.L
prearticular.R
coronoid.R
splenial.R
angular.R
surangular.R
dentary.R
articular.R
prearticular.L
coronoid.L
splenial.L
angular.L
surangular.L
dentary.L
articular.L

Supplementary Figure 6 | Alternative modularity for *Archaeopteryx lithographica* (modified after Rauhut[12]). a, Left-right modularity based on original *Ward.D2* cluster. **b,** Left-right modularity based on *Ward.D2* cluster with unpaired bones excluded.

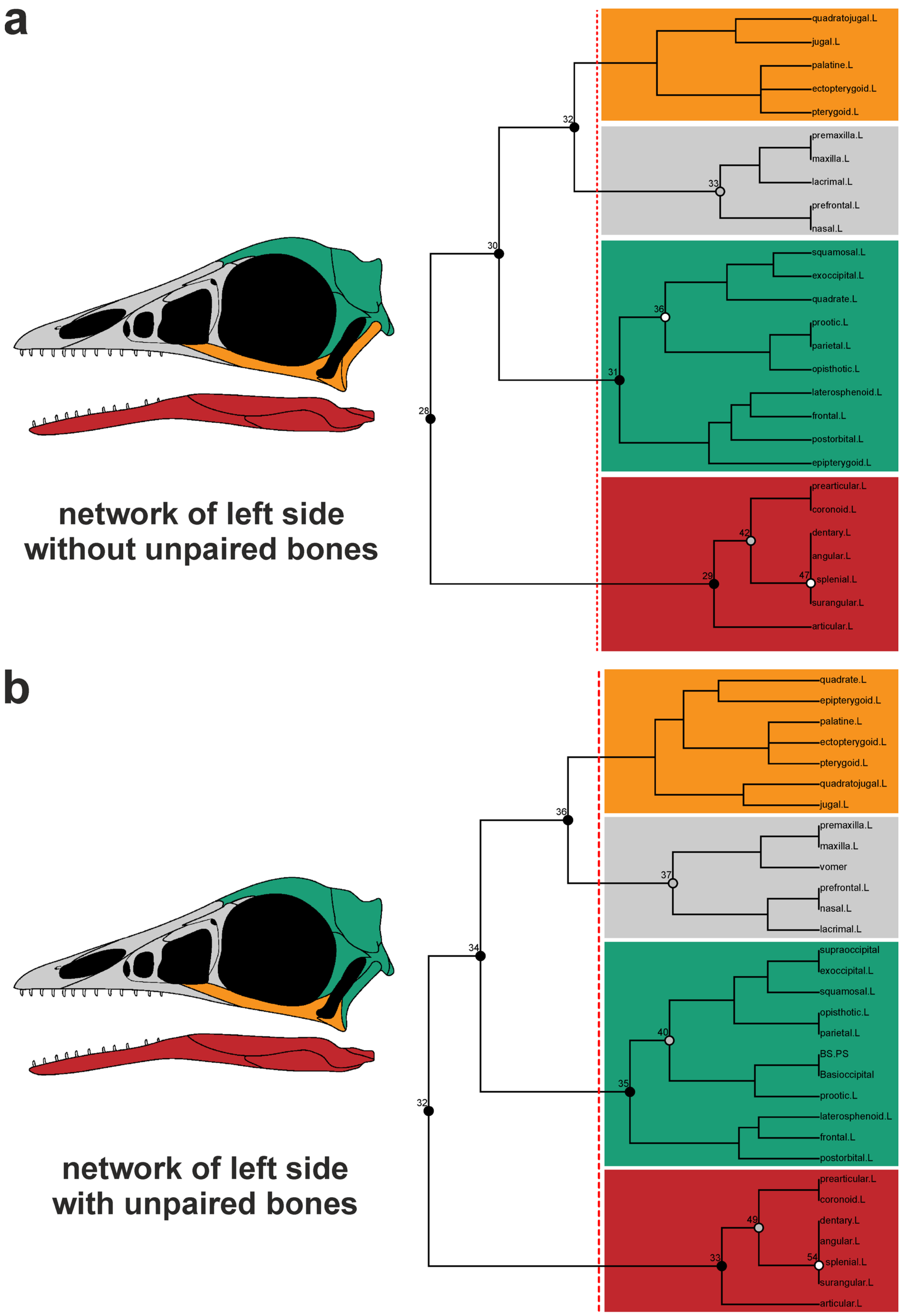
a
network of left side
without unpaired bones
quadratojugal.L
jugal.L
palatine.L
ectopterygoid.L
pterygoid.L
premaxilla.L
maxilla.L
lacrimal.L
prefrontal.L
nasal.L
squamosal.L
exoccipital.L
quadrate.L
prootic.L
parietal.L
opisthotic.L
laterosphenoid.L
frontal.L
postorbital.L
epipterygoid.L
prearticular.L
coronoid.L
dentary.L
angular.L
splenial.L
surangular.L
articular.L
28
29
30
31
32
33
36
42
47
b
network of left side
with unpaired bones
quadrate.L
epipterygoid.L
palatine.L
ectopterygoid.L
pterygoid.L
quadratojugal.L
jugal.L
premaxilla.L
maxilla.L
vomer
prefrontal.L
nasal.L
lacrimal.L
supraoccipital
exoccipital.L
squamosal.L
opisthotic.L
parietal.L
BS.PS
Basioccipital
prootic.L
laterosphenoid.L
frontal.L
postorbital.L
prearticular.L
coronoid.L
dentary.L
angular.L
splenial.L
surangular.L
articular.L
32
33
34
35
36
37
40
49
54

Supplementary Figure 7 | Alternative modularity for *Archaeopteryx lithographica* (modified after Rauhut[12]). a, Modularity based on original *Ward.D2* cluster including paired bones from the left skull side. **b,** Modularity based on original *Ward.D2* cluster including unpaired bones and paired bones from the left skull side.

4. References

1. Werneburg, I., Esteve-Altava, B., Bruno, J., Ladeira, M. T. & Diogo, R. Unique skull network complexity of *Tyrannosaurus rex* among land vertebrates. *Sci. Rep.* **9**, 1520 (2019).
2. Ericson, P. G. P. et al. Diversification of Neoaves: integration of molecular sequence data and fossils. *Biol. Lett.* **2**, 543–547 (2006).
3. Hackett, S. J. et al. A phylogenomic study of birds reveals their evolutionary history. *Science* **320**, 1763–1768 (2008).
4. Esteve-Altava, B. et al. Evolutionary parallelisms of pectoral and pelvic network-anatomy from fins to limbs. *Sci. Adv.* **5**, 7459 (2019).
5. Rasskin-Gutman, D. & Esteve-Altava, B. Connecting the dots: anatomical network analysis in morphological EvoDevo. *Biol. Theory* **9**, 178–193 (2014).
6. Goswami, A. & Finarelli, J. A. EMMLi: A maximum likelihood approach to the analysis of modularity. *Evolution* **70**, 1622–1637 (2016).
7. Felice, R. N. & Goswami, A. Developmental origins of mosaic evolution in the avian cranium. *Proc. Natl. Acad. Sci. U.S.A.* **115**, 555–560 (2018).
8. Felice, R. N. et al. Evolutionary integration and modularity in the archosaur cranium. *Integr. Comp. Biol.* **59**, 371–382 (2019).
9. Cardini, A. Integration and modularity in Procrustes shape data: is there a risk of spurious results? *Evol. Biol.* **46**, 90–105 (2019).
10. Hurum, J. H. & Sabath, K. Giant theropod dinosaurs from Asia and North America: skulls of *Tarbosaurus bataar* and *Tyrannosaurus rex* compared. *Acta Palaeontol. Pol.* **48**, 161–190 (2003).
11. Carr, T. D. Craniofacial ontogeny in Tyrannosauridae (Dinosauria, Coelurosauria). *J. Vertebr. Paleontol.* **19**, 497–520 (1999).

12. Rauhut, O. W. M. New observations on the skull of *Archaeopteryx*. *Pal. Z.* **88**, 211–221 (2014).

5. Skull network analysis results

The skull modularity of non-avian dinosaurs and the ontogenetic pairs of crown birds is documented by the network model and the *Ward.2D* cluster. The latter shows the hierarchical organization of each anatomical network, in which the horizontal dashed line marks the partition into *Q*-modules, while filled circles at nodes mark the statistical significance of *S*-modules (white, $p\text{-value} < 0.05$; grey, $p\text{-value} < 0.01$; black, $p\text{-value} < 0.001$). Based on the *Q*-modules in the cluster, the network and a skull reconstruction were colour coded to illustrate skull modularity. All sources for skull reconstructions are listed in Supplementary data 2 file.

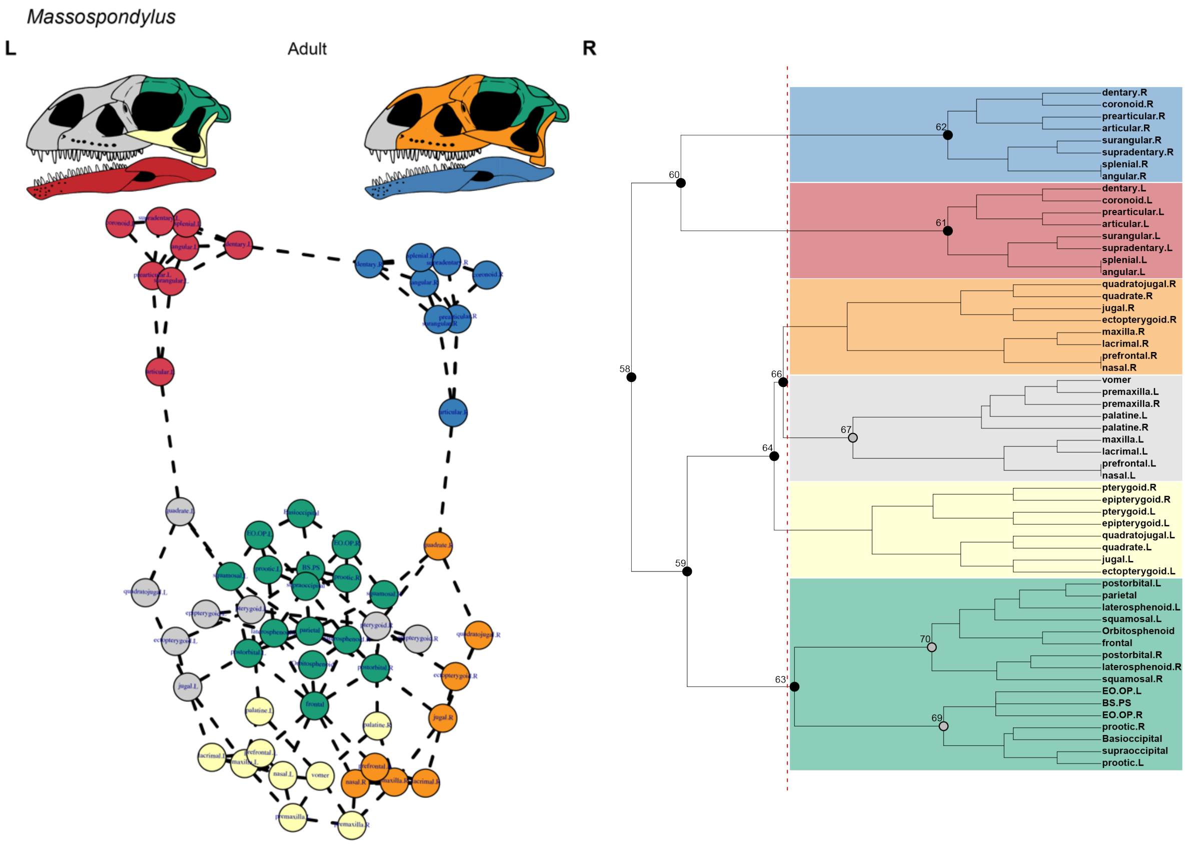
Massospondylus
L
Adult
R
dentary.R
coronoid.R
prearticular.R
articular.R
surangular.R
supradentary.R
splenial.R
angular.R
dentary.L
coronoid.L
prearticular.L
articular.L
surangular.L
supradentary.L
splenial.L
angular.L
quadratojugal.R
quadrate.R
jugal.R
ectopterygoid.R
maxilla.R
lacrimal.R
prefrontal.R
nasal.R
vomer
premaxilla.L
premaxilla.R
palatine.L
palatine.R
maxilla.L
lacrimal.L
prefrontal.L
nasal.L
pterygoid.R
epipterygoid.R
pterygoid.L
epipterygoid.L
quadratojugal.L
quadrate.L
jugal.L
ectopterygoid.L
postorbital.L
parietal
laterosphenoid.L
squamosal.L
Orbitosphenoid
frontal
postorbital.R
laterosphenoid.R
squamosal.R
EO.OP.L
BS.PS
EO.OP.R
prootic.R
Basioccipital
supraoccipital
prootic.L
62
61
60
58
66
67
64
59
70
63
69

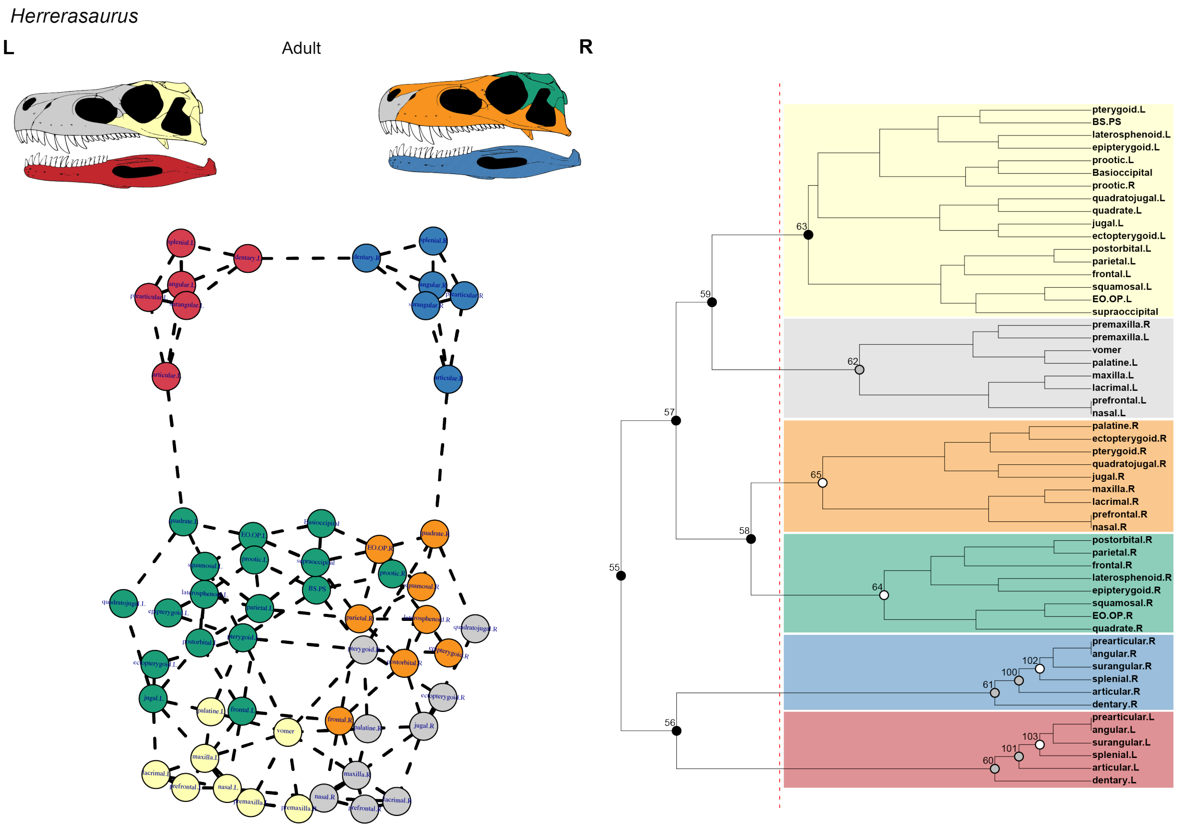
Herrerasaurus
L
Adult
R
pterygoid.L
BS.PS
laterosphenoid.L
epipterygoid.L
prootic.L
Basioccipital
prootic.R
quadratojugal.L
quadrate.L
jugal.L
ectopterygoid.L
postorbital.L
parietal.L
frontal.L
squamosal.L
EO.OP.L
supraoccipital
premaxilla.R
premaxilla.L
vomer
palatine.L
maxilla.L
lacrimal.L
prefrontal.L
nasal.L
palatine.R
ectopterygoid.R
pterygoid.R
quadratojugal.R
jugal.R
maxilla.R
lacrimal.R
prefrontal.R
nasal.R
postorbital.R
parietal.R
frontal.R
laterosphenoid.R
epipterygoid.R
squamosal.R
EO.OP.R
quadrate.R
prearticular.R
angular.R
surangular.R
splenial.R
articular.R
dentary.R
prearticular.L
angular.L
surangular.L
splenial.L
articular.L
dentary.L
63
59
62
57
65
58
55
64
102
100
61
56
103
101
60

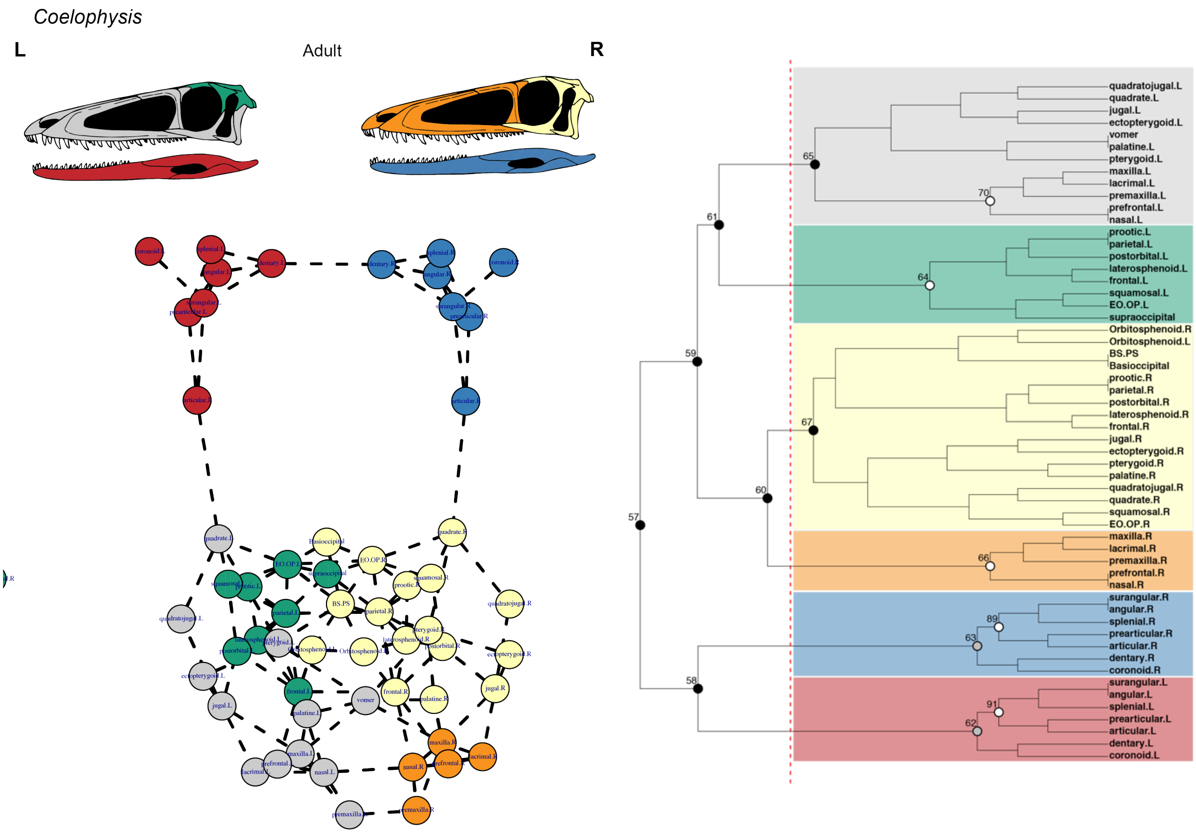
Coelophysis
L
Adult
R
quadratojugal.L
quadrate.L
jugal.L
ectopterygoid.L
vomer
palatine.L
pterygoid.L
maxilla.L
lacrimal.L
premaxilla.L
prefrontal.L
nasal.L
prootic.L
parietal.L
postorbital.L
laterosphenoid.L
frontal.L
squamosal.L
EO.OP.L
supraoccipital
Orbitosphenoid.R
Orbitosphenoid.L
BS.PS
Basioccipital
prootic.R
parietal.R
postorbital.R
laterosphenoid.R
frontal.R
jugal.R
ectopterygoid.R
pterygoid.R
palatine.R
quadratojugal.R
quadrate.R
squamosal.R
EO.OP.R
maxilla.R
lacrimal.R
premaxilla.R
prefrontal.R
nasal.R
surangular.R
angular.R
splenial.R
prearticular.R
articular.R
dentary.R
coronoid.R
surangular.L
angular.L
splenial.L
prearticular.L
articular.L
dentary.L
coronoid.L

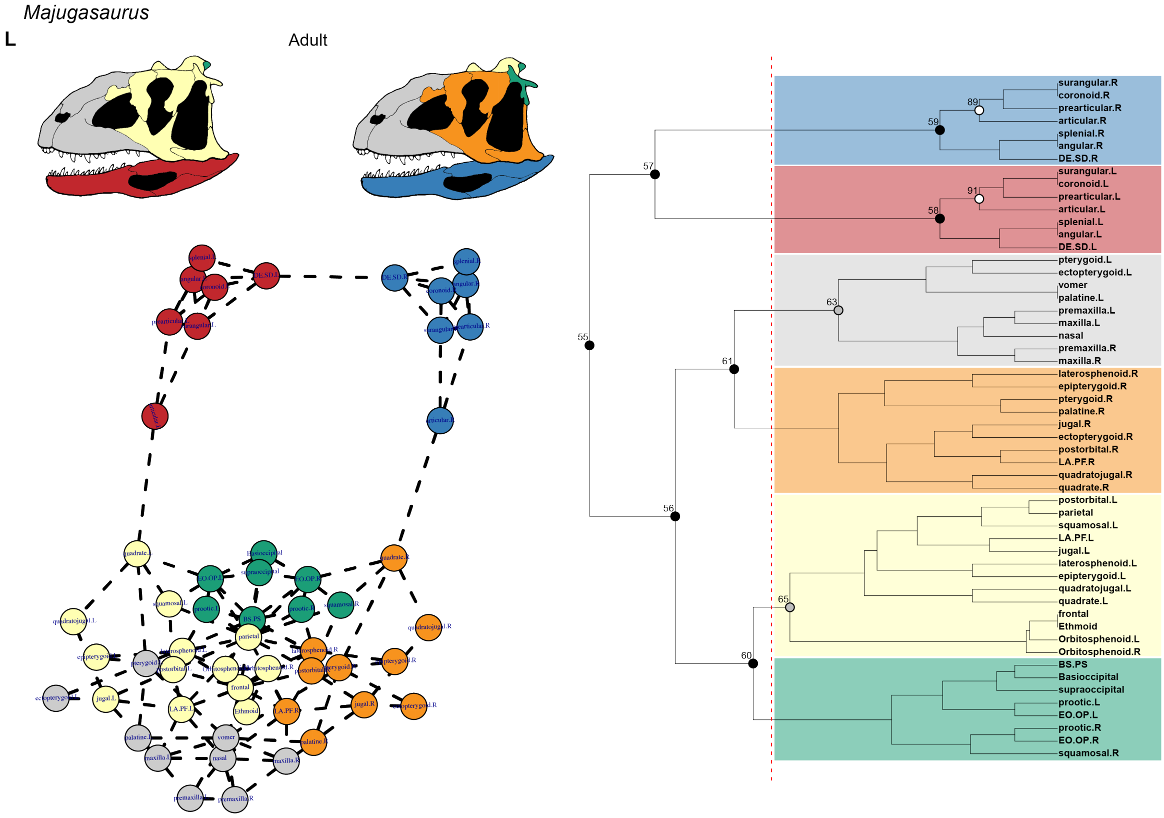
Majugasaurus
L
Adult
surangular.R
coronoid.R
prearticular.R
articular.R
splenial.R
angular.R
DE.SD.R
surangular.L
coronoid.L
prearticular.L
articular.L
splenial.L
angular.L
DE.SD.L
pterygoid.L
ectopterygoid.L
vomer
palatine.L
premaxilla.L
maxilla.L
nasal
premaxilla.R
maxilla.R
laterosphenoid.R
epipterygoid.R
pterygoid.R
palatine.R
jugal.R
ectopterygoid.R
postorbital.R
LA.PF.R
quadratojugal.R
quadrate.R
postorbital.L
parietal
squamosal.L
LA.PF.L
jugal.L
laterosphenoid.L
epipterygoid.L
quadratojugal.L
quadrate.L
frontal
Ethmoid
Orbitosphenoid.L
Orbitosphenoid.R
BS.PS
Basioccipital
supraoccipital
prootic.L
EO.OP.L
prootic.R
EO.OP.R
squamosal.R

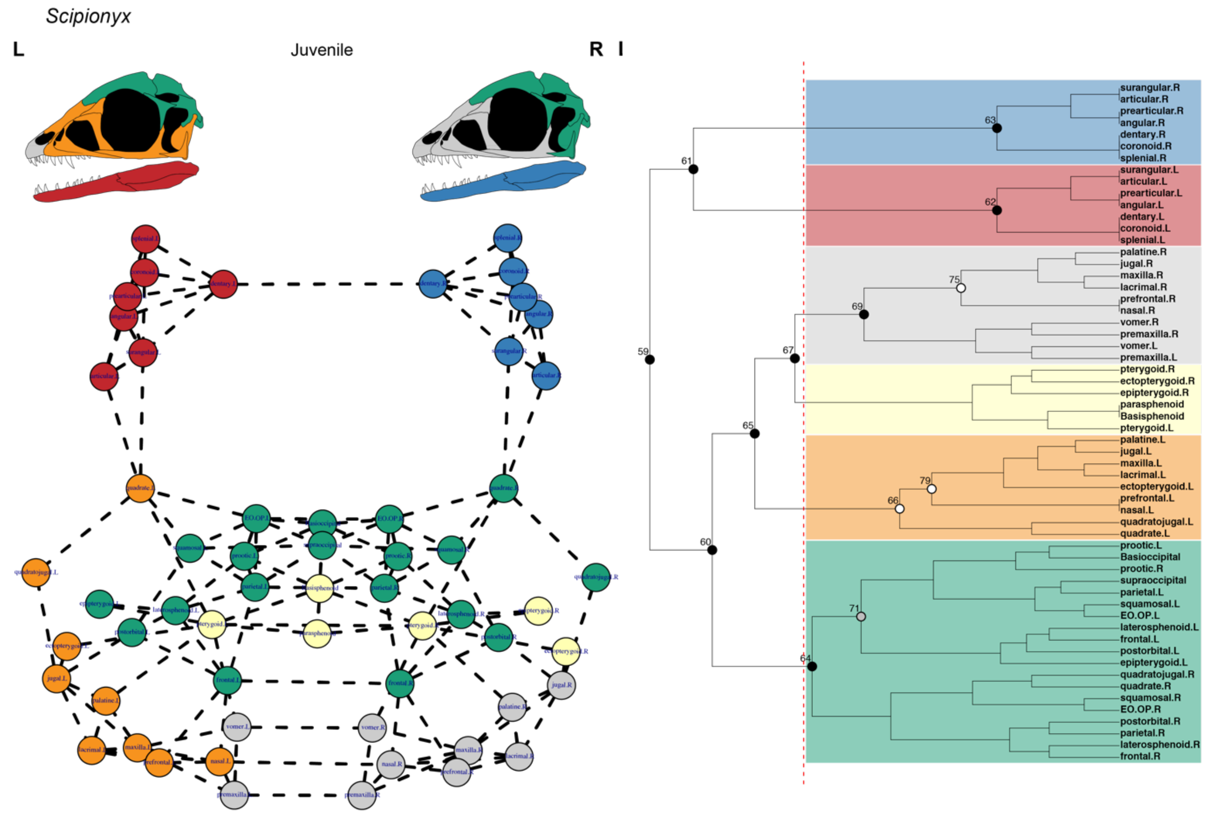
Scipionyx
L
Juvenile
R
I
surangular.R
articular.R
prearticular.R
angular.R
dentary.R
coronoid.R
splenial.R
surangular.L
articular.L
prearticular.L
angular.L
dentary.L
coronoid.L
splenial.L
palatine.R
jugal.R
maxilla.R
lacrimal.R
prefrontal.R
nasal.R
vomer.R
premaxilla.R
vomer.L
premaxilla.L
pterygoid.R
ectopterygoid.R
epipterygoid.R
parasphenoid
Basisphenoid
pterygoid.L
palatine.L
jugal.L
maxilla.L
lacrimal.L
ectopterygoid.L
prefrontal.L
nasal.L
quadratojugal.L
quadrate.L
prootic.L
Basioccipital
prootic.R
supraoccipital
parietal.L
squamosal.L
EO.OP.L
laterosphenoid.L
frontal.L
postorbital.L
epipterygoid.L
quadratojugal.R
quadrate.R
squamosal.R
EO.OP.R
postorbital.R
parietal.R
laterosphenoid.R
frontal.R
59
60
61
62
63
64
65
66
67
69
71
75
79

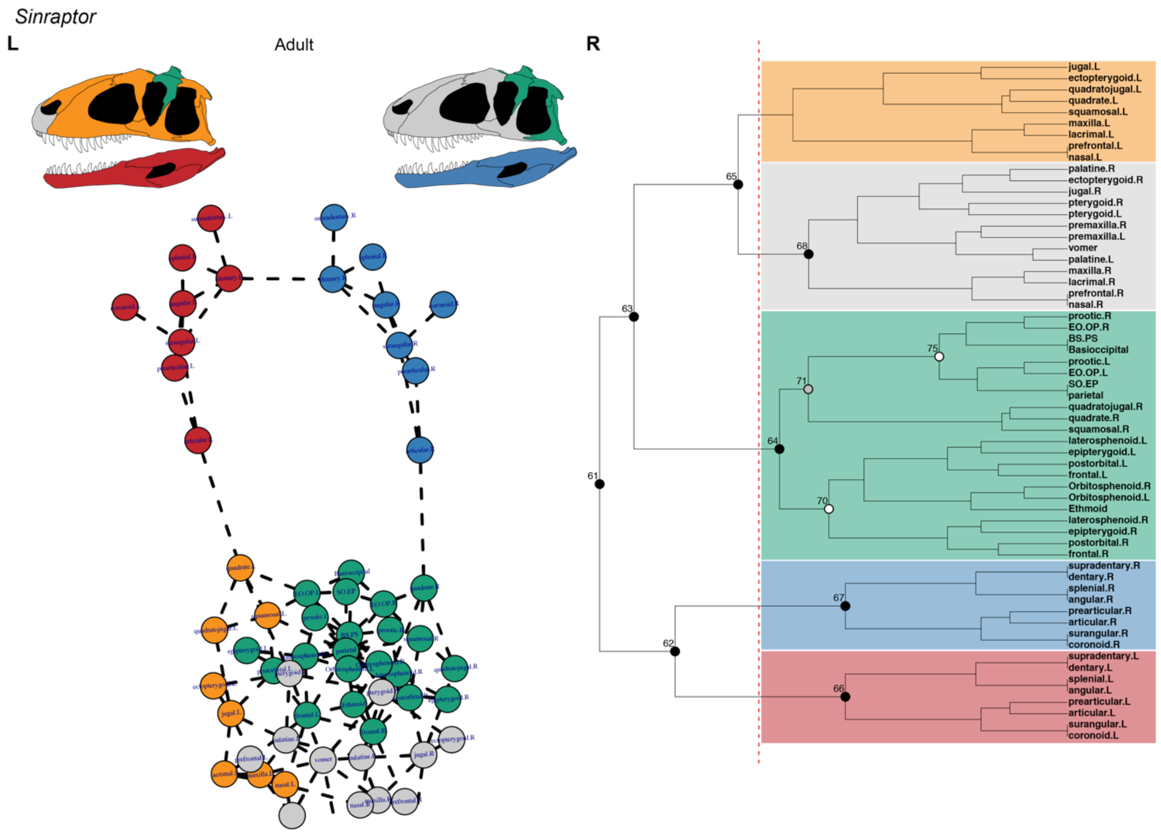
Sinraptor
L
Adult
R
jugal.L
ectopterygoid.L
quadratojugal.L
quadrate.L
squamosal.L
maxilla.L
lacrimal.L
prefrontal.L
nasal.L
palatine.R
ectopterygoid.R
jugal.R
pterygoid.R
pterygoid.L
premaxilla.R
premaxilla.L
vomer
palatine.L
maxilla.R
lacrimal.R
prefrontal.R
nasal.R
prootic.R
EO.OP.R
BS.PS
Basioccipital
prootic.L
EO.OP.L
SO.EP
parietal
quadratojugal.R
quadrate.R
squamosal.R
laterosphenoid.L
epipterygoid.L
postorbital.L
frontal.L
Orbitosphenoid.R
Orbitosphenoid.L
Ethmoid
laterosphenoid.R
epipterygoid.R
postorbital.R
frontal.R
supradentary.R
dentary.R
splenial.R
angular.R
prearticular.R
articular.R
surangular.R
coronoid.R
supradentary.L
dentary.L
splenial.L
angular.L
prearticular.L
articular.L
surangular.L
coronoid.L
61
62
63
64
65
66
67
68
70
71
75

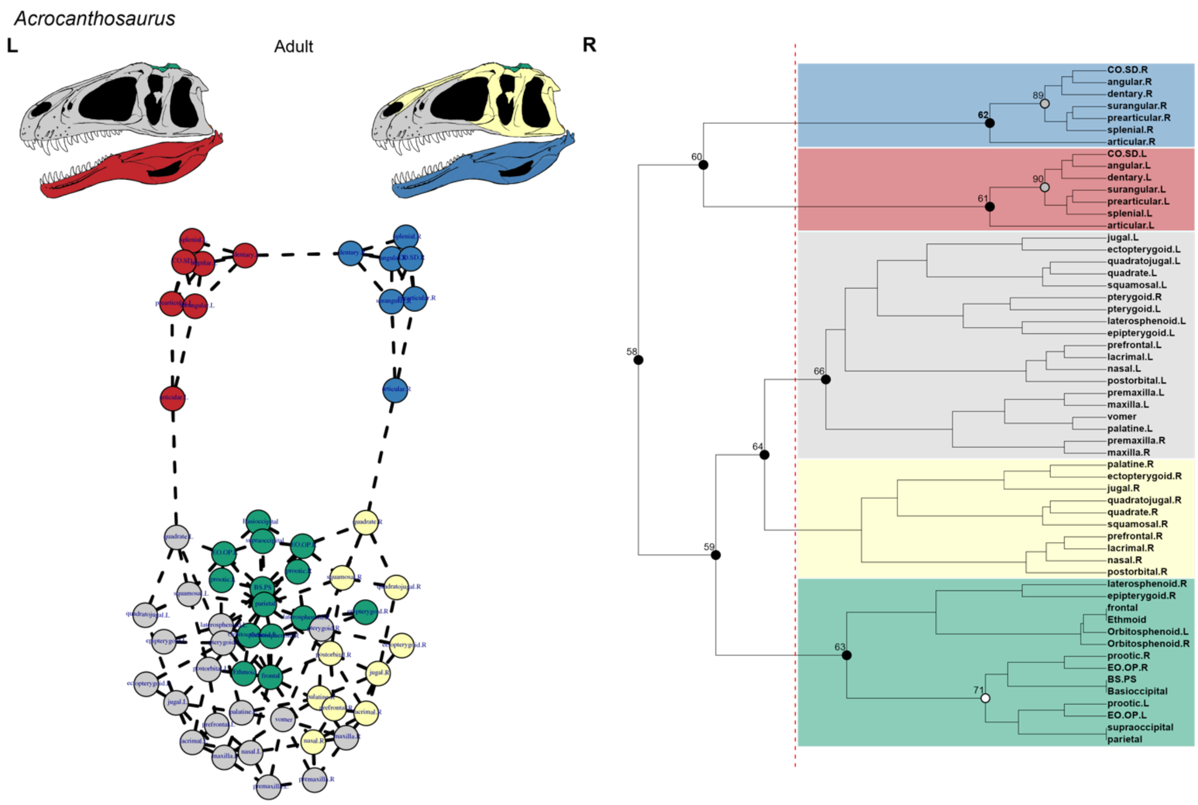
Acrocanthosaurus
L
Adult
R
CO.SD.R
angular.R
dentary.R
surangular.R
prearticular.R
splenial.R
articular.R
CO.SD.L
angular.L
dentary.L
surangular.L
prearticular.L
splenial.L
articular.L
jugal.L
ectopterygoid.L
quadratojugal.L
quadrate.L
squamosal.L
pterygoid.R
pterygoid.L
laterosphenoid.L
epipterygoid.L
prefrontal.L
lacrimal.L
nasal.L
postorbital.L
premaxilla.L
maxilla.L
vomer
palatine.L
premaxilla.R
maxilla.R
palatine.R
ectopterygoid.R
jugal.R
quadratojugal.R
quadrate.R
squamosal.R
prefrontal.R
lacrimal.R
nasal.R
postorbital.R
laterosphenoid.R
epipterygoid.R
frontal
Ethmoid
Orbitosphenoid.L
Orbitosphenoid.R
prootic.R
EO.OP.R
BS.PS
Basioccipital
prootic.L
EO.OP.L
supraoccipital
parietal
89
62
60
90
61
58
66
64
59
63
71

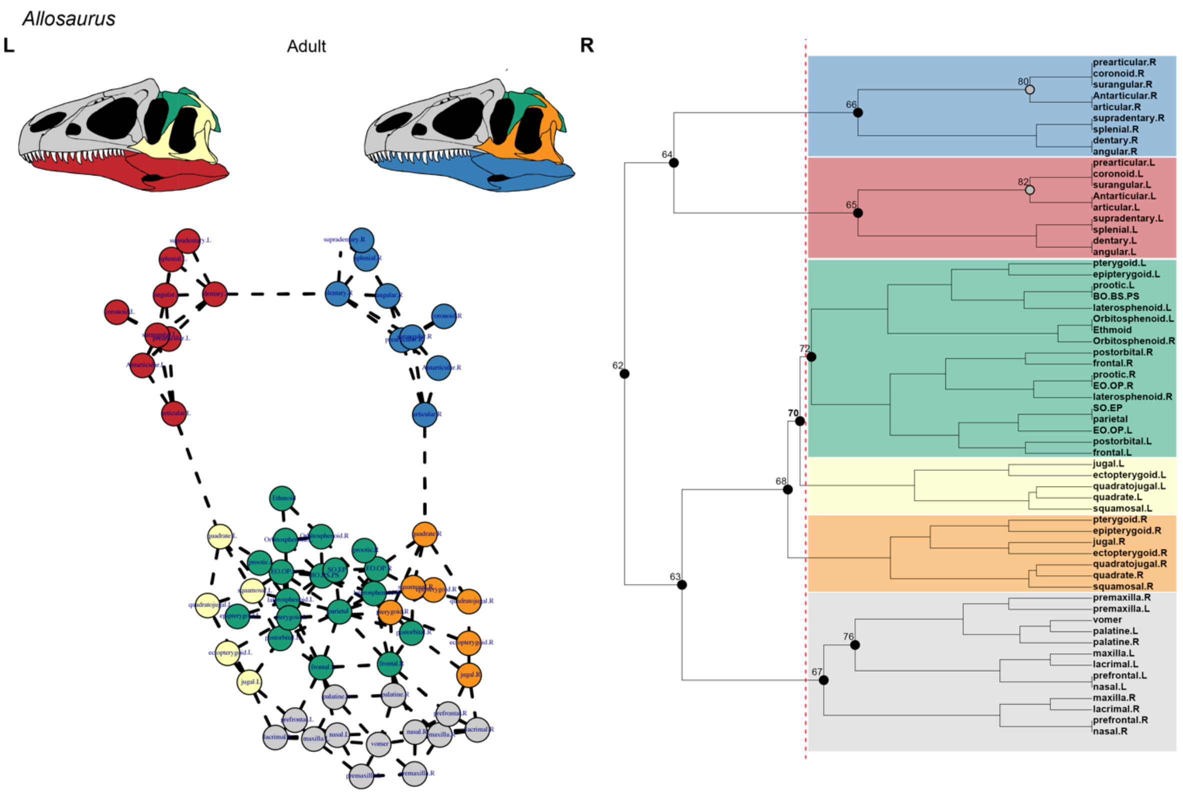
Allosaurus
L
Adult
R
prearticular.R
coronoid.R
surangular.R
Antarticular.R
articular.R
supradentary.R
splenial.R
dentary.R
angular.R
prearticular.L
coronoid.L
surangular.L
Antarticular.L
articular.L
supradentary.L
splenial.L
dentary.L
angular.L
pterygoid.L
epipterygoid.L
prootic.L
BO.BS.PS
laterosphenoid.L
Orbitosphenoid.L
Ethmoid
Orbitosphenoid.R
postorbital.R
frontal.R
prootic.R
EO.OP.R
laterosphenoid.R
SO.EP
parietal
EO.OP.L
postorbital.L
frontal.L
jugal.L
ectopterygoid.L
quadratojugal.L
quadrate.L
squamosal.L
pterygoid.R
epipterygoid.R
jugal.R
ectopterygoid.R
quadratojugal.R
quadrate.R
squamosal.R
premaxilla.R
premaxilla.L
vomer
palatine.L
palatine.R
maxilla.L
lacrimal.L
prefrontal.L
nasal.L
maxilla.R
lacrimal.R
prefrontal.R
nasal.R
80
66
64
82
65
62
72
70
68
63
76
67

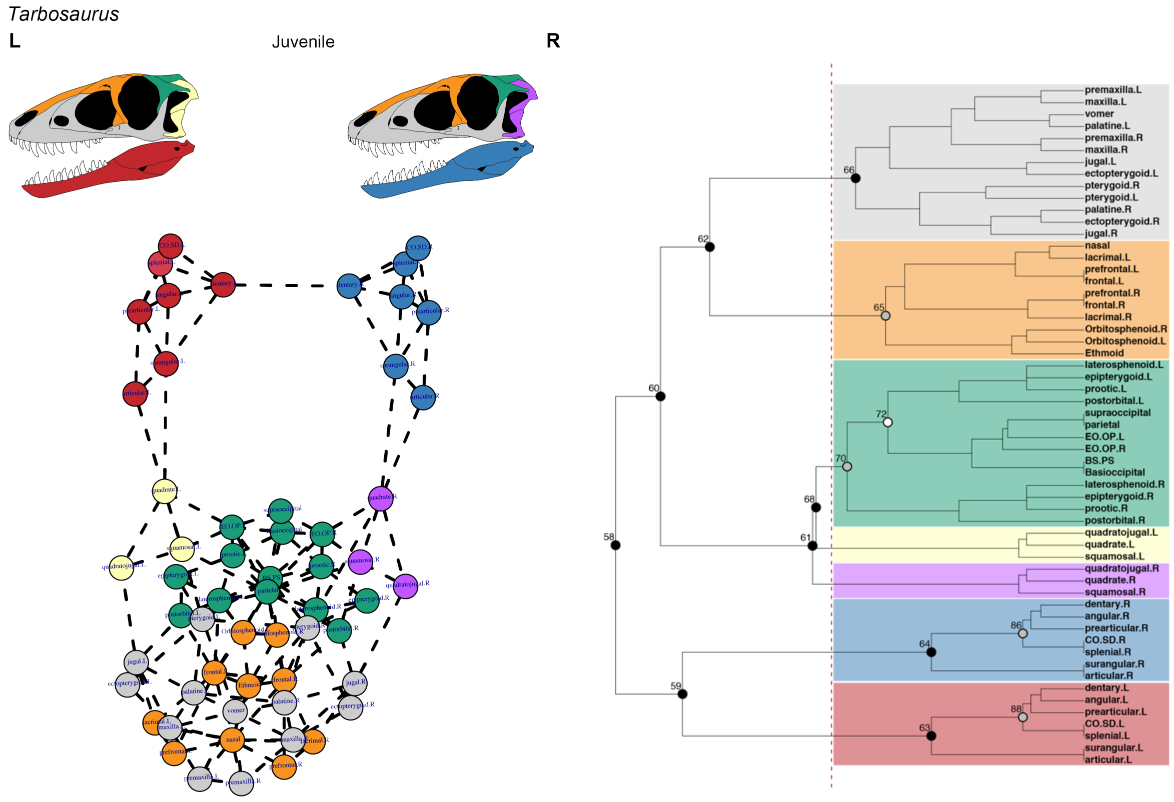
Tarbosaurus
L
Juvenile
R
premaxilla.L
maxilla.L
vomer
palatine.L
premaxilla.R
maxilla.R
jugal.L
ectopterygoid.L
pterygoid.R
pterygoid.L
palatine.R
ectopterygoid.R
jugal.R
nasal
lacrimal.L
prefrontal.L
frontal.L
prefrontal.R
frontal.R
lacrimal.R
Orbitosphenoid.R
Orbitosphenoid.L
Ethmoid
laterosphenoid.L
epipterygoid.L
prootic.L
postorbital.L
supraoccipital
parietal
EO.OP.L
EO.OP.R
BS.PS
Basioccipital
laterosphenoid.R
epipterygoid.R
prootic.R
postorbital.R
quadratojugal.L
quadrate.L
squamosal.L
quadratojugal.R
quadrate.R
squamosal.R
dentary.R
angular.R
prearticular.R
CO.SD.R
splenial.R
surangular.R
articular.R
dentary.L
angular.L
prearticular.L
CO.SD.L
splenial.L
surangular.L
articular.L

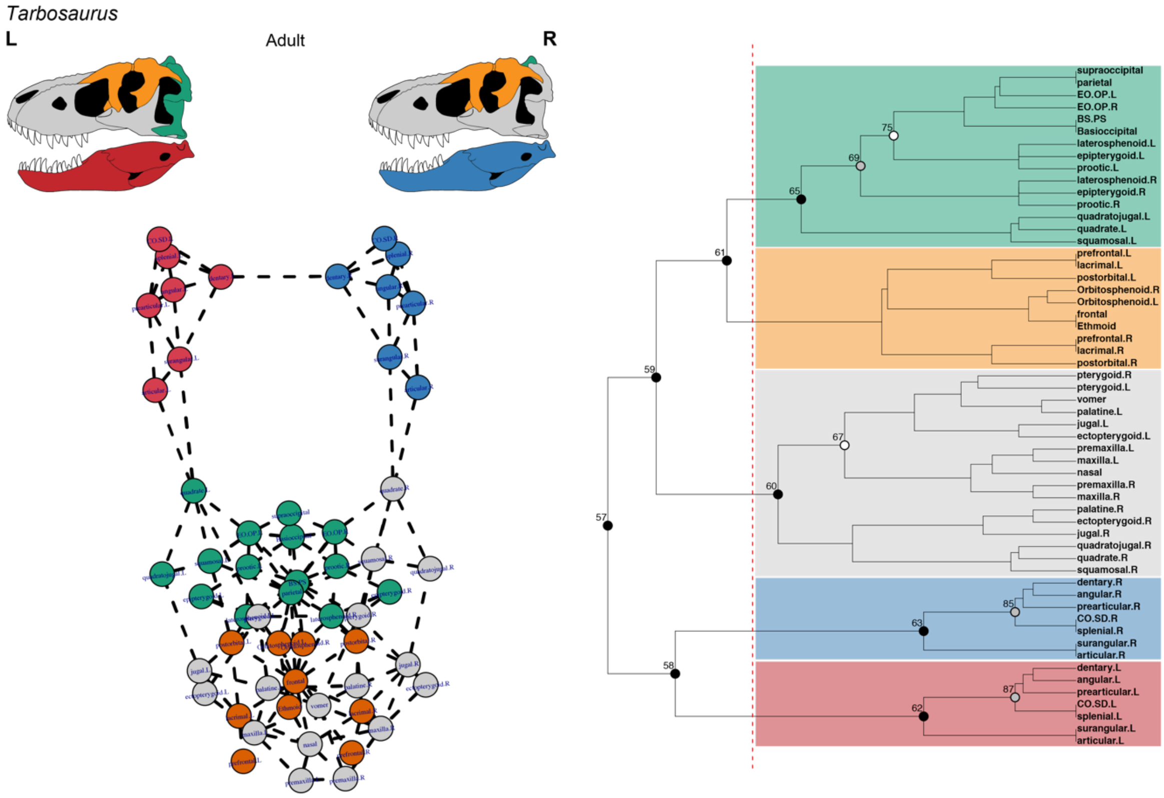
Tarbosaurus
L
Adult
R
supraoccipital
parietal
EO.OP.L
EO.OP.R
BS.PS
Basioccipital
laterosphenoid.L
epipterygoid.L
prootic.L
laterosphenoid.R
epipterygoid.R
prootic.R
quadratojugal.L
quadrate.L
squamosal.L
prefrontal.L
lacrimal.L
postorbital.L
Orbitosphenoid.R
Orbitosphenoid.L
frontal
Ethmoid
prefrontal.R
lacrimal.R
postorbital.R
pterygoid.R
pterygoid.L
vomer
palatine.L
jugal.L
ectopterygoid.L
premaxilla.L
maxilla.L
nasal
premaxilla.R
maxilla.R
palatine.R
ectopterygoid.R
jugal.R
quadratojugal.R
quadrate.R
squamosal.R
dentary.R
angular.R
prearticular.R
CO.SD.R
splenial.R
surangular.R
articular.R
dentary.L
angular.L
prearticular.L
CO.SD.L
splenial.L
surangular.L
articular.L

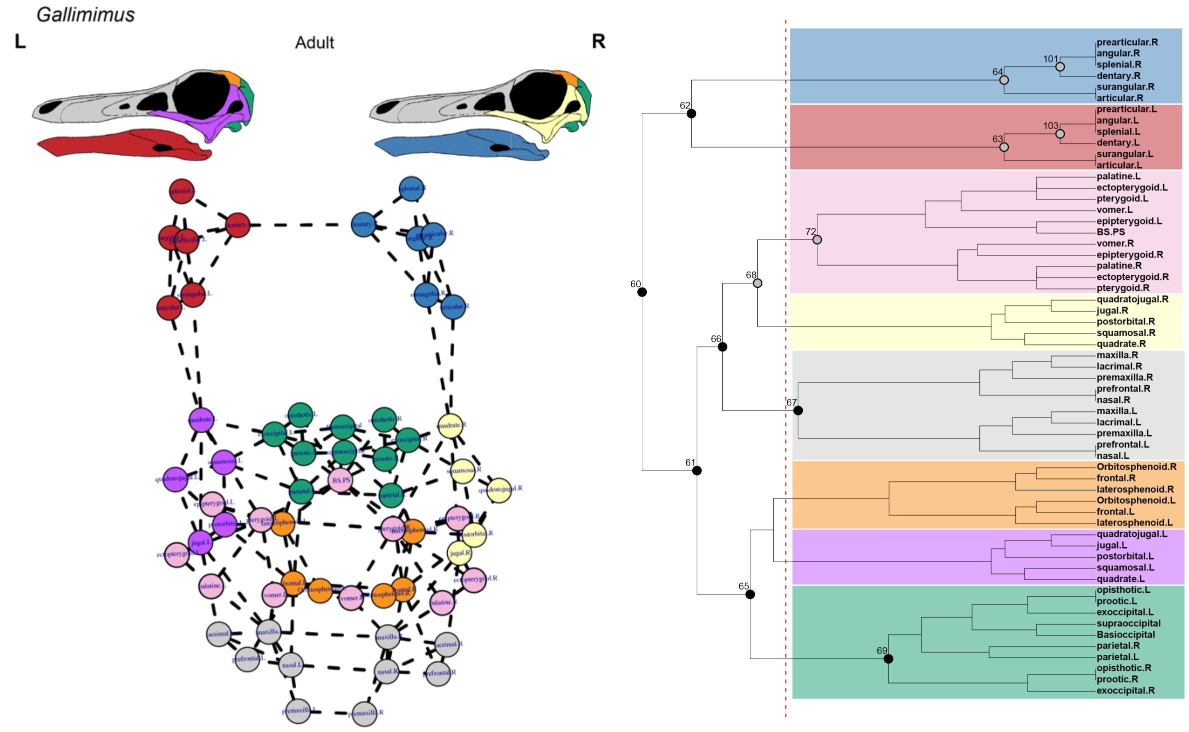

Gallimimus
L
Adult
R
prearticular.R
angular.R
splenial.R
dentary.R
surangular.R
articular.R
prearticular.L
angular.L
splenial.L
dentary.L
surangular.L
articular.L
palatine.L
ectopterygoid.L
pterygoid.L
vomer.L
epipterygoid.L
BS.PS
vomer.R
epipterygoid.R
palatine.R
ectopterygoid.R
pterygoid.R
quadratojugal.R
jugal.R
postorbital.R
squamosal.R
quadrate.R
maxilla.R
lacrimal.R
premaxilla.R
prefrontal.R
nasal.R
maxilla.L
lacrimal.L
premaxilla.L
prefrontal.L
nasal.L
Orbitosphenoid.R
frontal.R
laterosphenoid.R
Orbitosphenoid.L
frontal.L
laterosphenoid.L
quadratojugal.L
jugal.L
postorbital.L
squamosal.L
quadrate.L
opisthotic.L
prootic.L
exoccipital.L
supraoccipital
Basioccipital
parietal.R
parietal.L
opisthotic.R
prootic.R
exoccipital.R

5

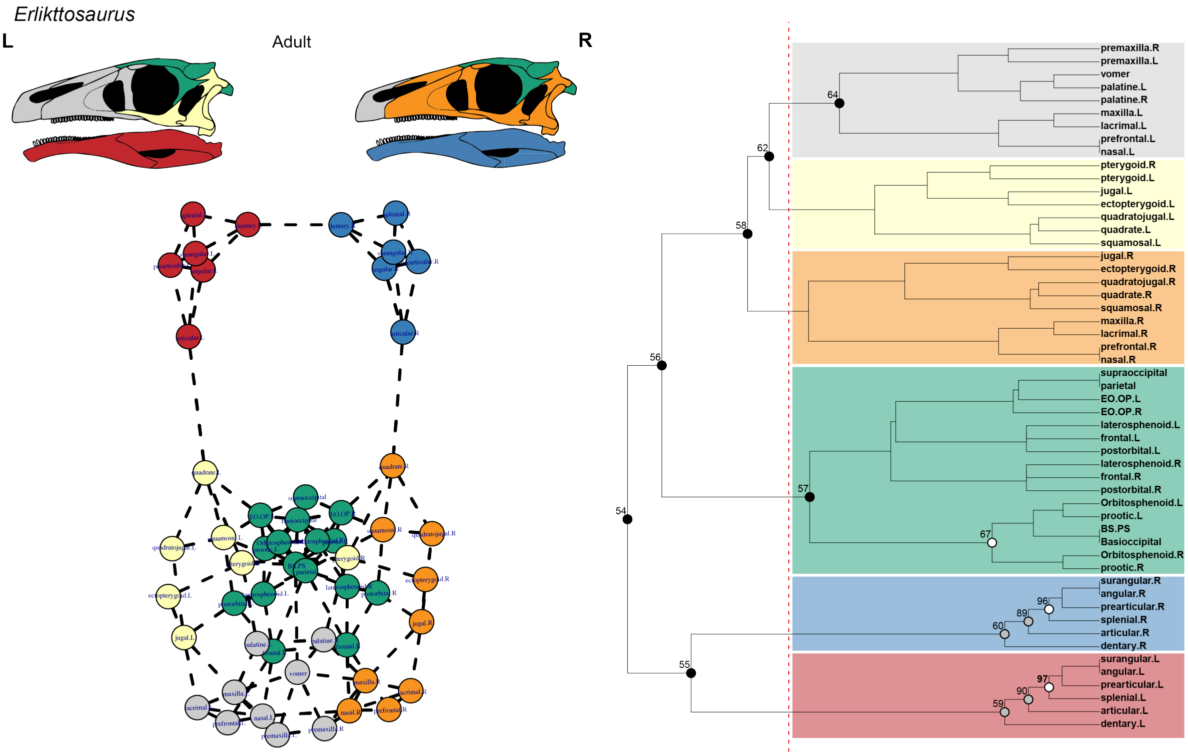

Erlikttosaurus
L
Adult
R
premaxilla.R
premaxilla.L
vomer
palatine.L
palatine.R
maxilla.L
lacrimal.L
prefrontal.L
nasal.L
pterygoid.R
pterygoid.L
jugal.L
ectopterygoid.L
quadratojugal.L
quadrate.L
squamosal.L
jugal.R
ectopterygoid.R
quadratojugal.R
quadrate.R
squamosal.R
maxilla.R
lacrimal.R
prefrontal.R
nasal.R
supraoccipital
parietal
EO.OP.L
EO.OP.R
laterosphenoid.L
frontal.L
postorbital.L
laterosphenoid.R
frontal.R
postorbital.R
Orbitosphenoid.L
prootic.L
BS.PS
Basioccipital
Orbitosphenoid.R
prootic.R
surangular.R
angular.R
prearticular.R
splenial.R
articular.R
dentary.R
surangular.L
angular.L
prearticular.L
splenial.L
articular.L
dentary.L

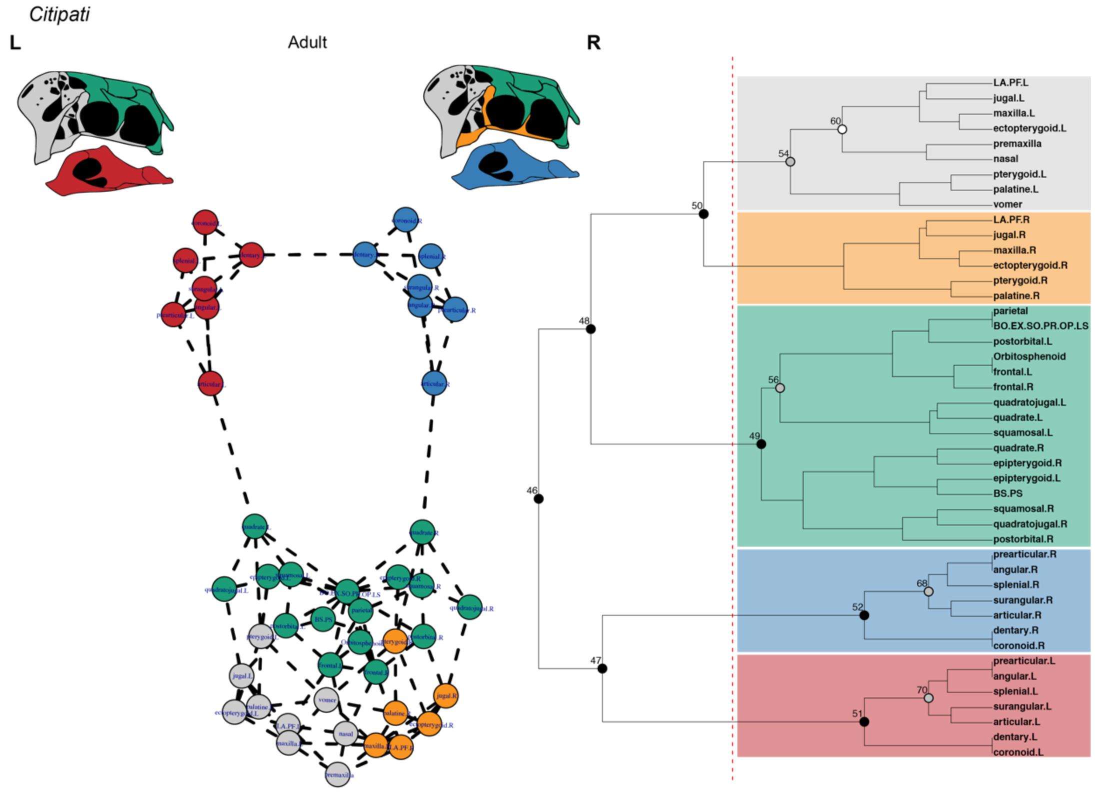
Citipati
L
Adult
R
LA.PF.L
jugal.L
maxilla.L
ectopterygoid.L
premaxilla
nasal
pterygoid.L
palatine.L
vomer
LA.PF.R
jugal.R
maxilla.R
ectopterygoid.R
pterygoid.R
palatine.R
parietal
BO.EX.SO.PR.OP.LS
postorbital.L
Orbitosphenoid
frontal.L
frontal.R
quadratojugal.L
quadrate.L
squamosal.L
quadrate.R
epipterygoid.R
epipterygoid.L
BS.PS
squamosal.R
quadratojugal.R
postorbital.R
prearticular.R
angular.R
splenial.R
surangular.R
articular.R
dentary.R
coronoid.R
prearticular.L
angular.L
splenial.L
surangular.L
articular.L
dentary.L
coronoid.L
60
54
50
48
56
49
46
68
52
47
70
51

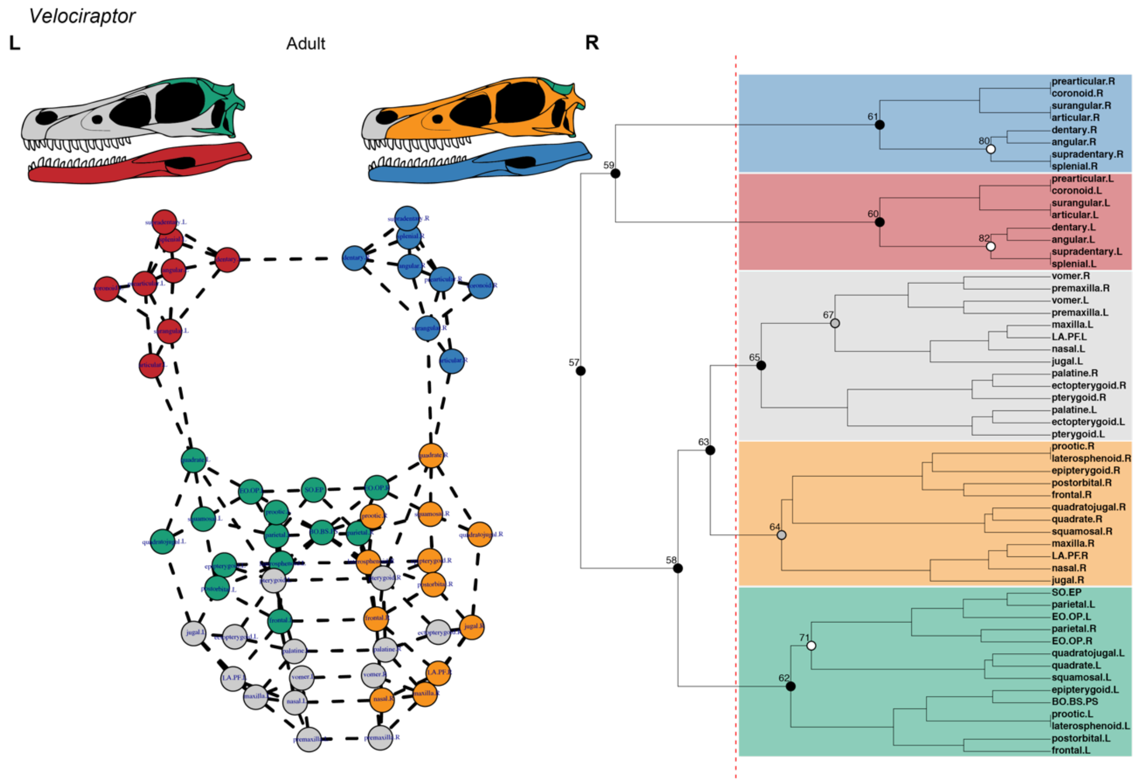
Velociraptor
L
Adult
R
prearticular.R
coronoid.R
surangular.R
articular.R
dentary.R
angular.R
supradentary.R
splenial.R
prearticular.L
coronoid.L
surangular.L
articular.L
dentary.L
angular.L
supradentary.L
splenial.L
vomer.R
premaxilla.R
vomer.L
premaxilla.L
maxilla.L
LA.PF.L
nasal.L
jugal.L
palatine.R
ectopterygoid.R
pterygoid.R
palatine.L
ectopterygoid.L
pterygoid.L
prootic.R
laterosphenoid.R
epipterygoid.R
postorbital.R
frontal.R
quadratojugal.R
quadrate.R
squamosal.R
maxilla.R
LA.PF.R
nasal.R
jugal.R
SO.EP
parietal.L
EO.OP.L
parietal.R
EO.OP.R
quadratojugal.L
quadrate.L
squamosal.L
epipterygoid.L
BO.BS.PS
prootic.L
laterosphenoid.L
postorbital.L
frontal.L
61
80
59
60
82
67
65
57
63
64
58
71
62

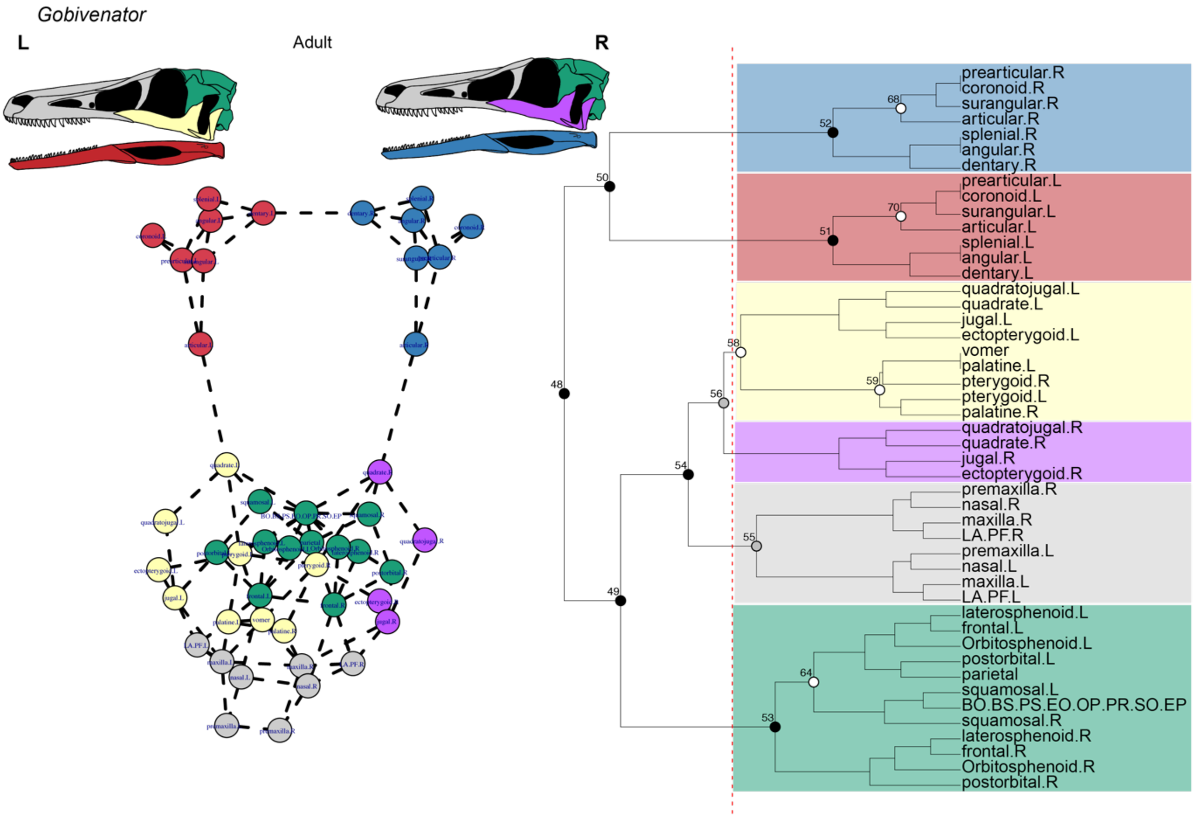
Gobivenator
L
Adult
R
48
49
50
51
52
53
54
55
56
58
59
64
68
70
prearticular.R
coronoid.R
surangular.R
articular.R
splenial.R
angular.R
dentary.R
prearticular.L
coronoid.L
surangular.L
articular.L
splenial.L
angular.L
dentary.L
quadratojugal.L
quadrate.L
jugal.L
ectopterygoid.L
vomer
palatine.L
pterygoid.R
pterygoid.L
palatine.R
quadratojugal.R
quadrate.R
jugal.R
ectopterygoid.R
premaxilla.R
nasal.R
maxilla.R
LA.PF.R
premaxilla.L
nasal.L
maxilla.L
LA.PF.L
laterosphenoid.L
frontal.L
Orbitosphenoid.L
postorbital.L
parietal
squamosal.L
BO.BS.PS.EO.OP.PR.SO.EP
squamosal.R
laterosphenoid.R
frontal.R
Orbitosphenoid.R
postorbital.R

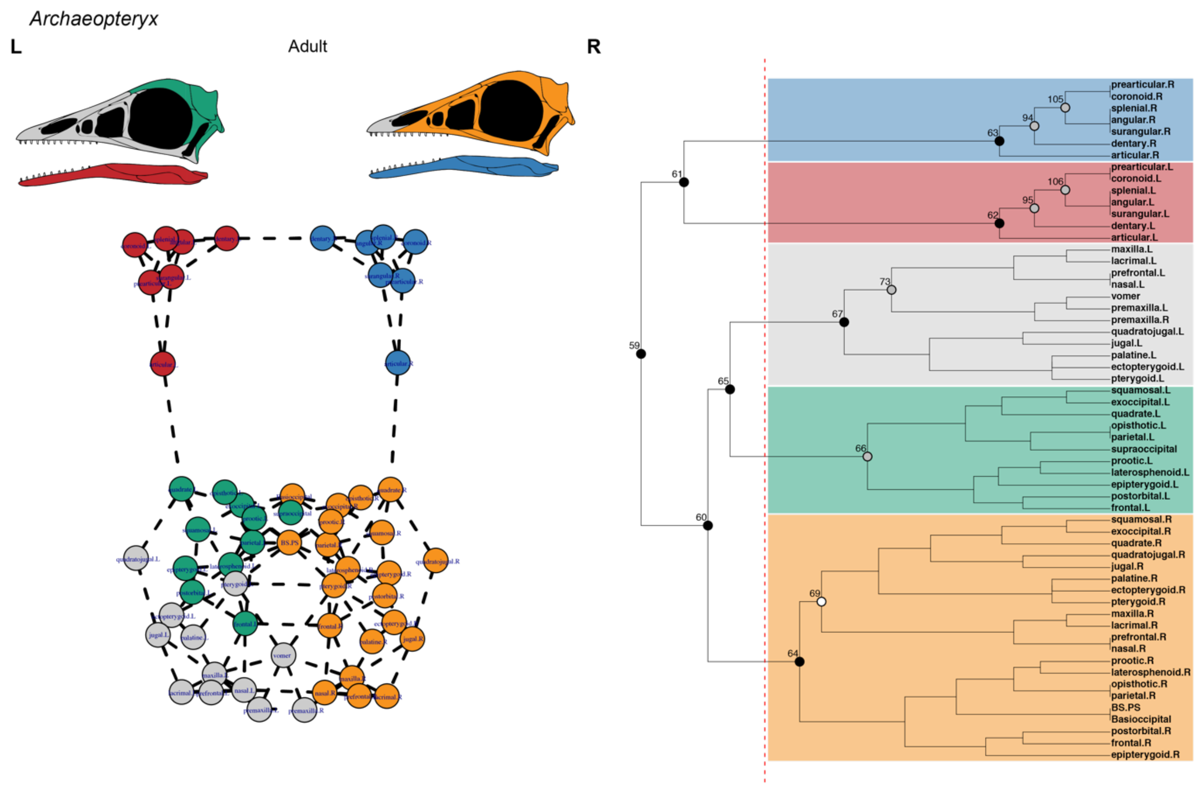

Archaeopteryx
L
Adult
R
105
94
63
61
106
95
62
73
67
59
65
66
60
69
64
prearticular.R
coronoid.R
splenial.R
angular.R
surangular.R
dentary.R
articular.R
prearticular.L
coronoid.L
splenial.L
angular.L
surangular.L
dentary.L
articular.L
maxilla.L
lacrimal.L
prefrontal.L
nasal.L
vomer
premaxilla.L
premaxilla.R
quadratojugal.L
jugal.L
palatine.L
ectopterygoid.L
pterygoid.L
squamosal.L
exoccipital.L
quadrate.L
opisthotic.L
parietal.L
supraoccipital
prootic.L
laterosphenoid.L
epipterygoid.L
postorbital.L
frontal.L
squamosal.R
exoccipital.R
quadrate.R
quadratojugal.R
jugal.R
palatine.R
ectopterygoid.R
pterygoid.R
maxilla.R
lacrimal.R
prefrontal.R
nasal.R
prootic.R
laterosphenoid.R
opisthotic.R
parietal.R
BS.PS
Basioccipital
postorbital.R
frontal.R
epipterygoid.R

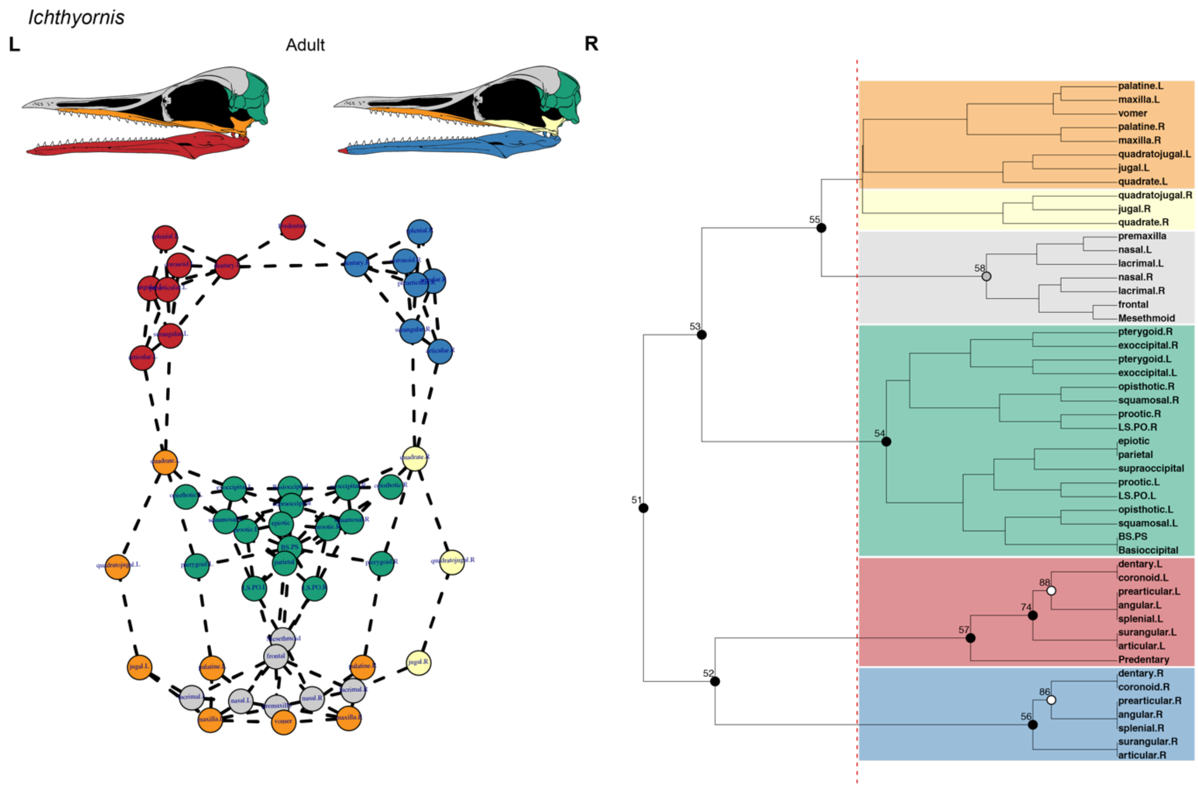

Ichthyornis
L
Adult
R
55
58
53
54
51
88
74
57
52
86
56
palatine.L
maxilla.L
vomer
palatine.R
maxilla.R
quadratojugal.L
jugal.L
quadrate.L
quadratojugal.R
jugal.R
quadrate.R
premaxilla
nasal.L
lacrimal.L
nasal.R
lacrimal.R
frontal
Mesethmoid
pterygoid.R
exoccipital.R
pterygoid.L
exoccipital.L
opisthotic.R
squamosal.R
prootic.R
LS.PO.R
epiotic
parietal
supraoccipital
prootic.L
LS.PO.L
opisthotic.L
squamosal.L
BS.PS
Basioccipital
dentary.L
coronoid.L
prearticular.L
angular.L
splenial.L
surangular.L
articular.L
Predentary
dentary.R
coronoid.R
prearticular.R
angular.R
splenial.R
surangular.R
articular.R

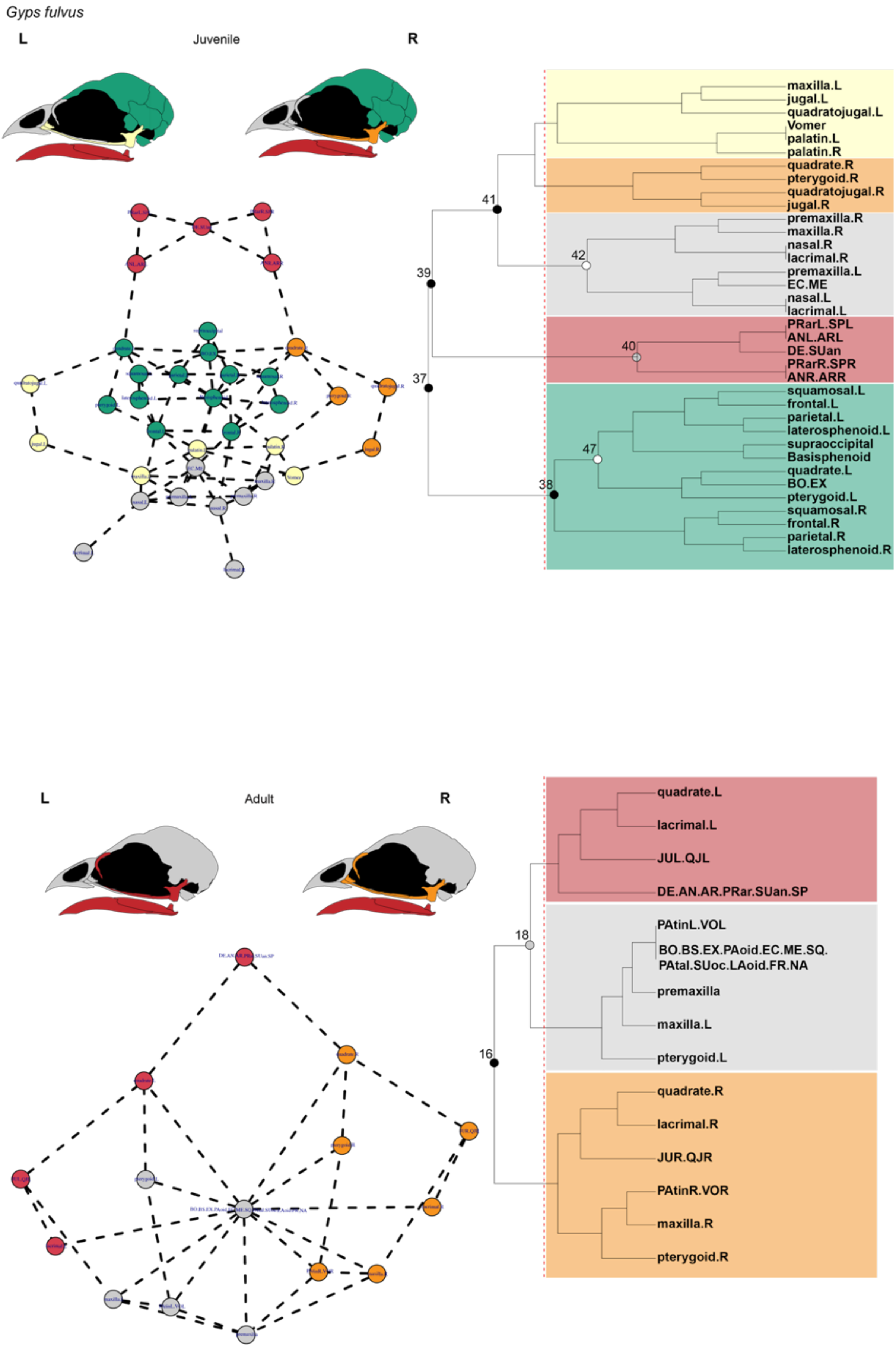

Gyps fulvus
L
Juvenile
R
maxilla.L
jugal.L
quadratojugal.L
Vomer
palatin.L
palatin.R
quadrate.R
pterygoid.R
quadratojugal.R
jugal.R
premaxilla.R
maxilla.R
nasal.R
lacrimal.R
premaxilla.L
EC.ME
nasal.L
lacrimal.L
PRarL.SPL
ANL.ARL
DE.SUan
PRarR.SPR
ANR.ARR
squamosal.L
frontal.L
parietal.L
laterosphenoid.L
supraoccipital
Basisphenoid
quadrate.L
BO.EX
pterygoid.L
squamosal.R
frontal.R
parietal.R
laterosphenoid.R
41
42
39
40
37
47
38
L
Adult
R
quadrate.L
lacrimal.L
JUL.QJL
DE.AN.AR.PRar.SUan.SP
PAtinL.VOL
BO.BS.EX.PAoid.EC.ME.SQ.
PAtal.SUoc.LAoid.FR.NA
premaxilla
maxilla.L
pterygoid.L
quadrate.R
lacrimal.R
JUR.QJR
PAtinR.VOR
maxilla.R
pterygoid.R
18
16

Gypaetus barbatus

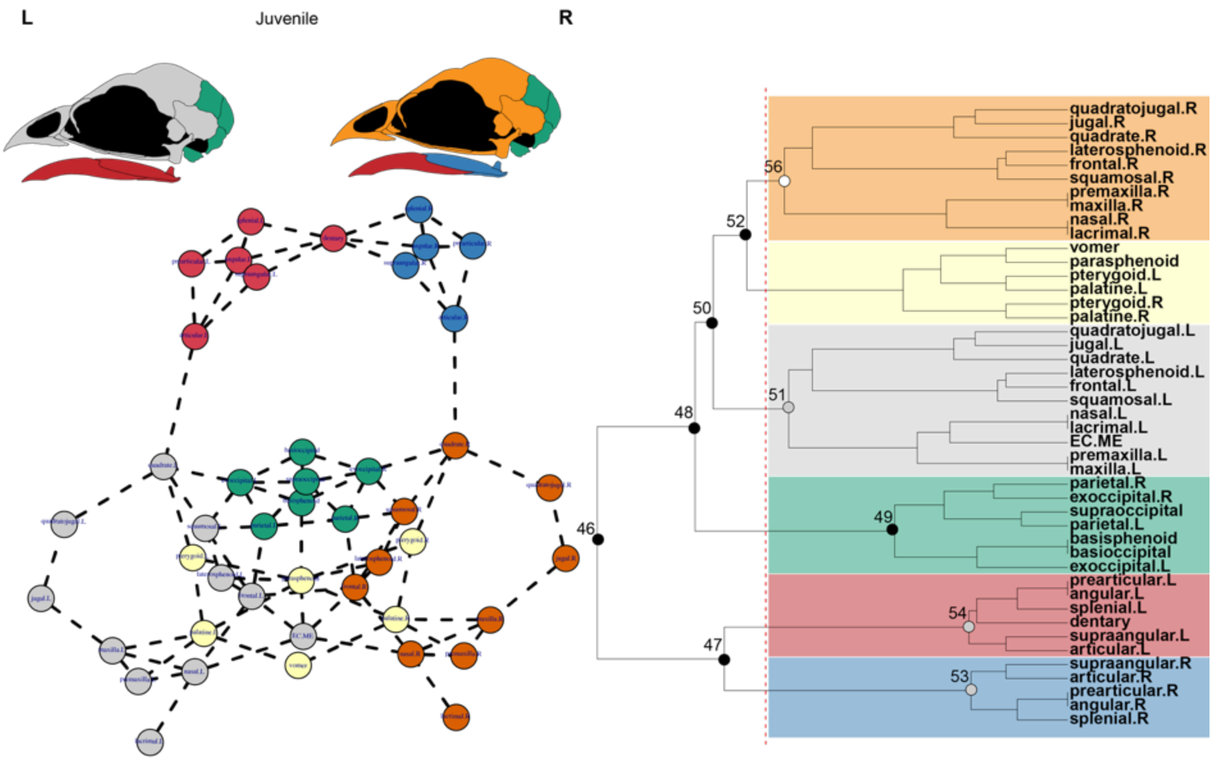

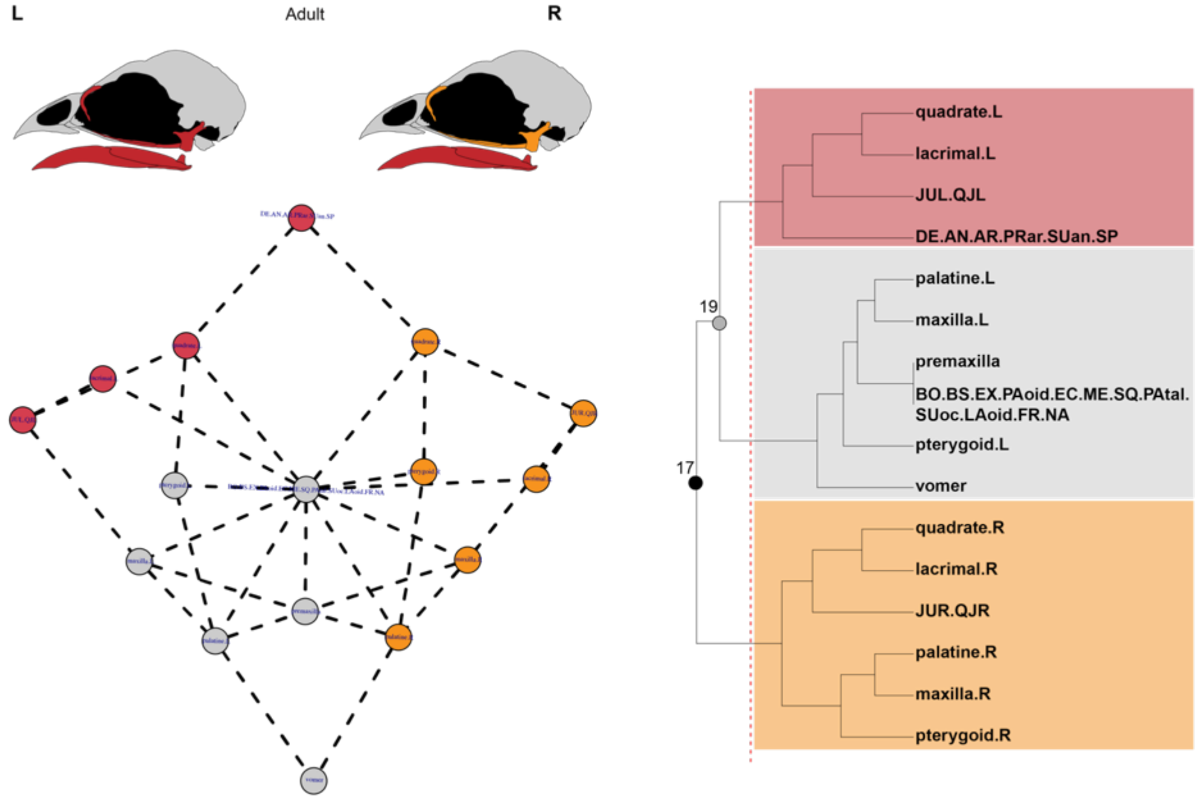

Milvus milvus

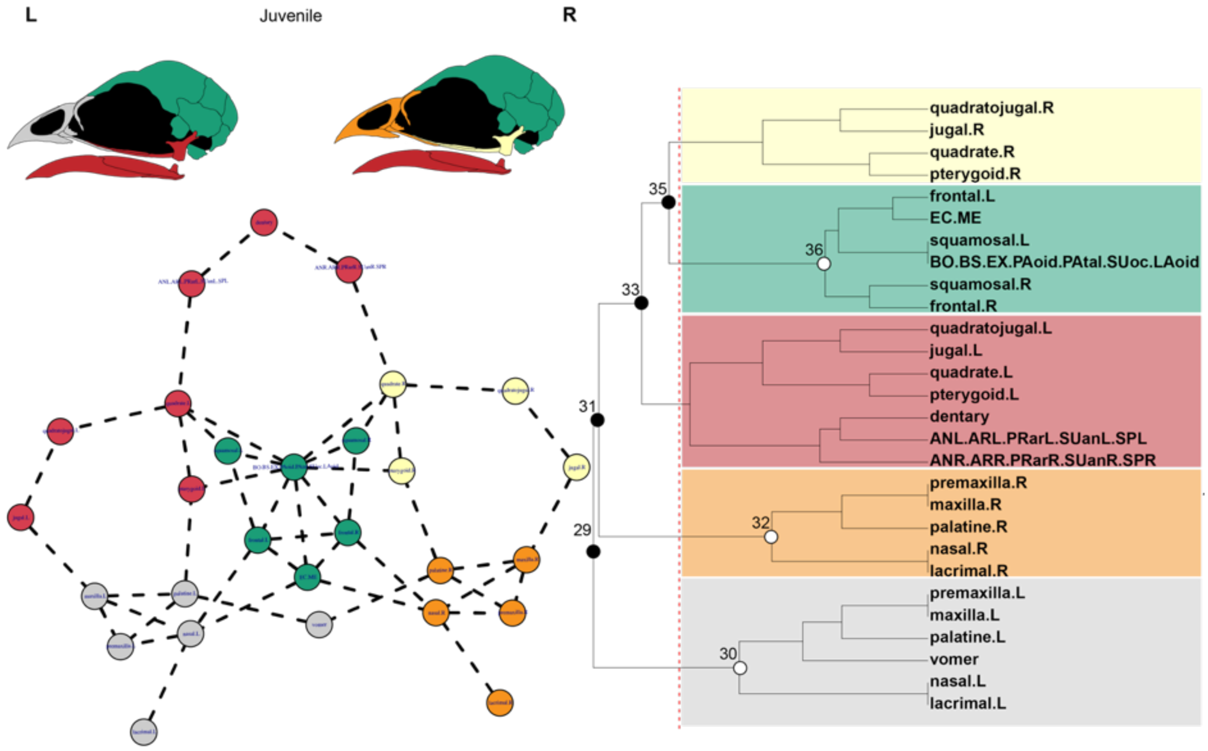

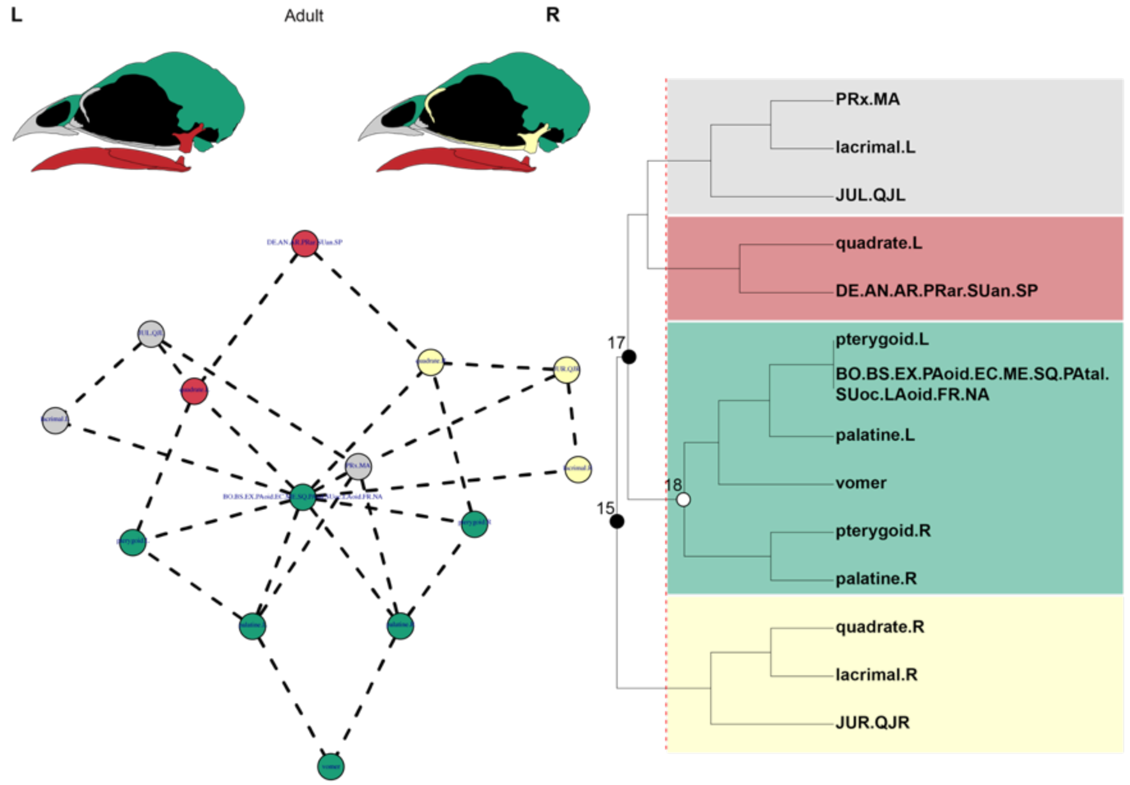

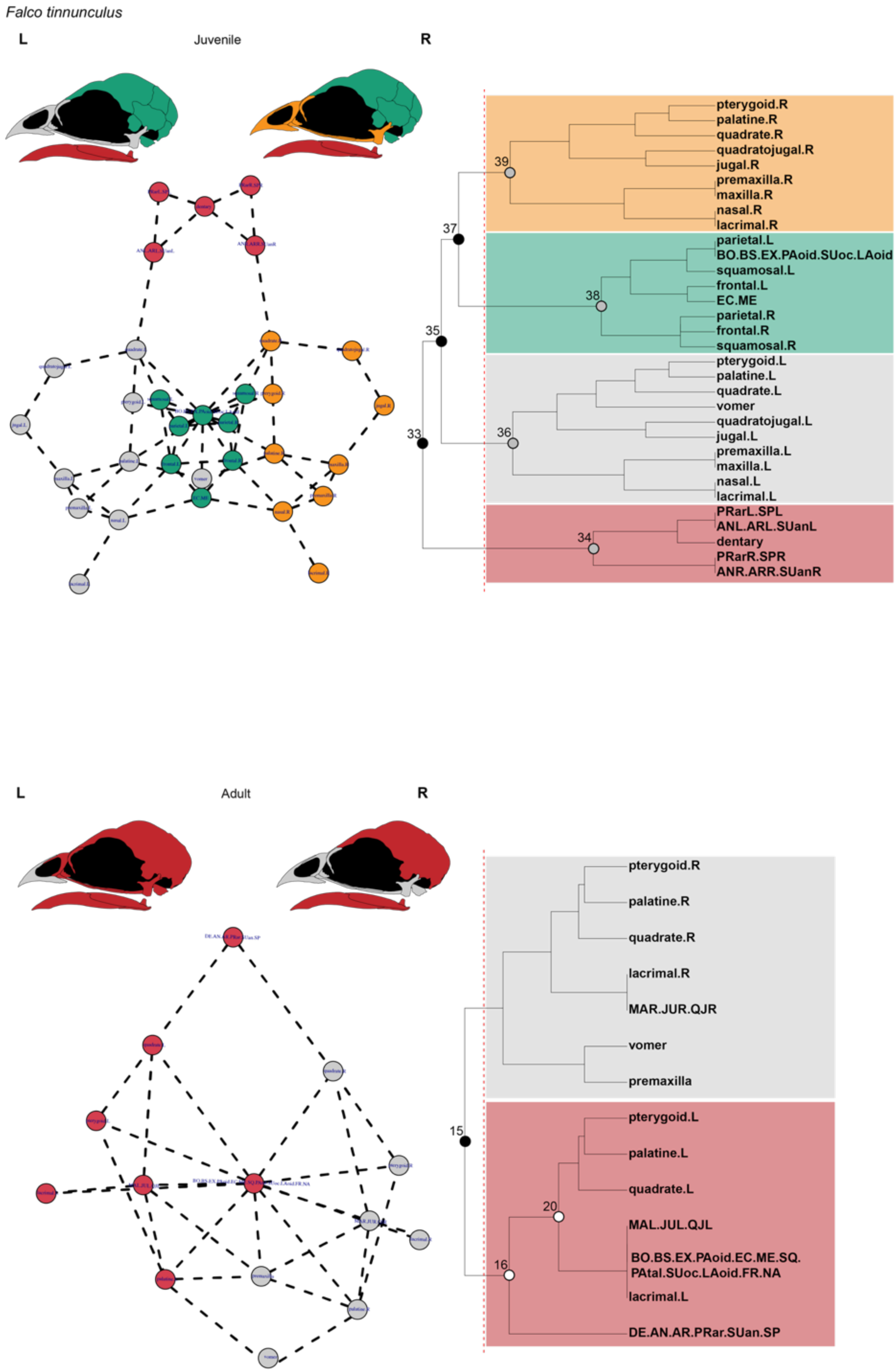

Falco tinnunculus
L
Juvenile
R
pterygoid.R
palatine.R
quadrate.R
quadratojugal.R
jugal.R
premaxilla.R
maxilla.R
nasal.R
lacrimal.R
parietal.L
BO.BS.EX.PAoid.SUoc.LAoid
squamosal.L
frontal.L
EC.ME
parietal.R
frontal.R
squamosal.R
pterygoid.L
palatine.L
quadrate.L
vomer
quadratojugal.L
jugal.L
premaxilla.L
maxilla.L
nasal.L
lacrimal.L
PRarL.SPL
ANL.ARL.SUanL
dentary
PRarR.SPR
ANR.ARR.SUanR
39
37
38
35
36
33
34
L
Adult
R
pterygoid.R
palatine.R
quadrate.R
lacrimal.R
MAR.JUR.QJR
vomer
premaxilla
pterygoid.L
palatine.L
quadrate.L
MAL.JUL.QJL
BO.BS.EX.PAoid.EC.ME.SQ.
PAtal.SUoc.LAoid.FR.NA
lacrimal.L
DE.AN.AR.PRar.SUan.SP
15
20
16

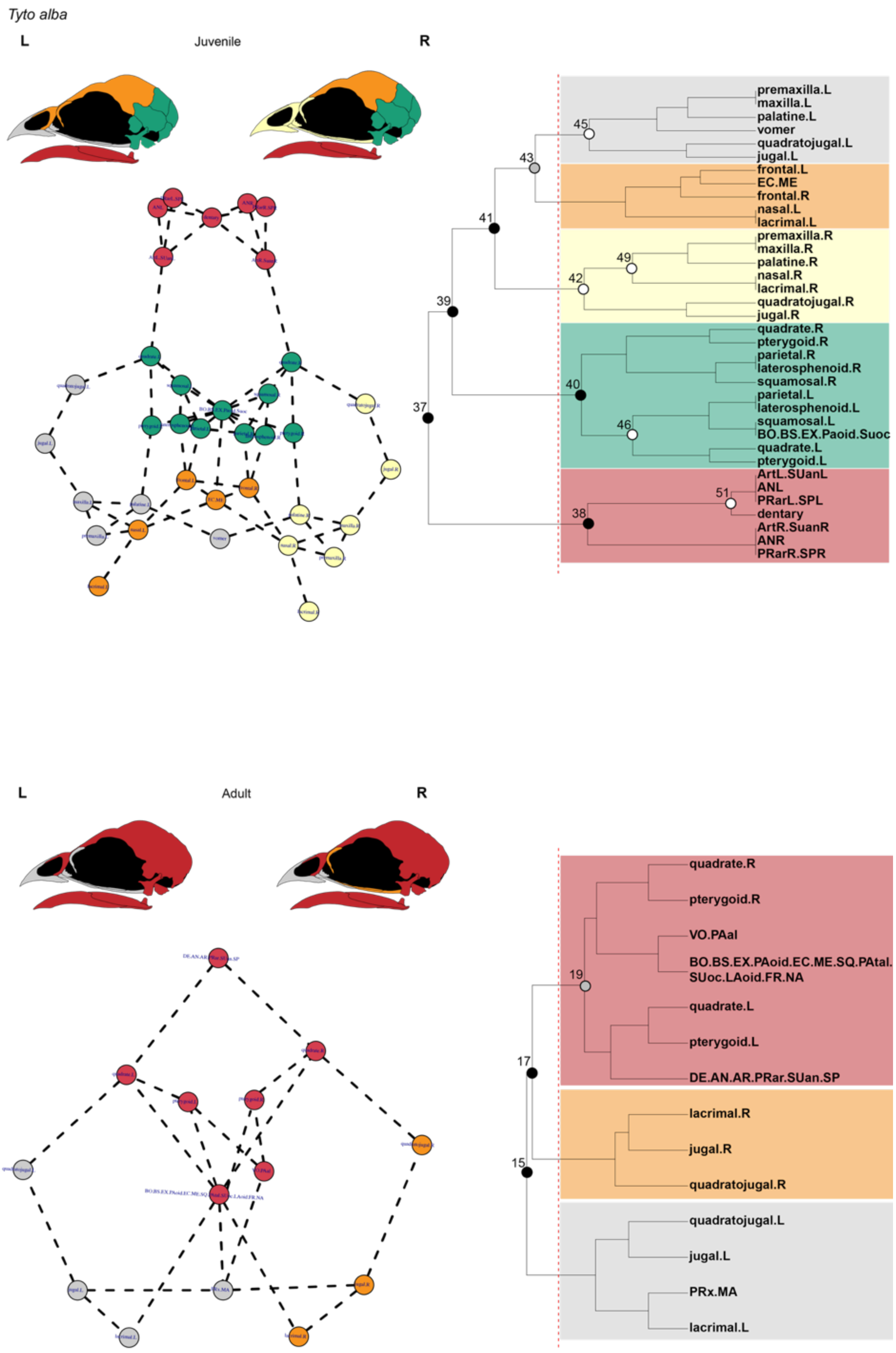

Tyto alba
L
Juvenile
R
premaxilla.L
maxilla.L
palatine.L
vomer
quadratojugal.L
jugal.L
frontal.L
EC.ME
frontal.R
nasal.L
lacrimal.L
premaxilla.R
maxilla.R
palatine.R
nasal.R
lacrimal.R
quadratojugal.R
jugal.R
quadrate.R
pterygoid.R
parietal.R
laterosphenoid.R
squamosal.R
parietal.L
laterosphenoid.L
squamosal.L
BO.BS.EX.Paoid.Suoc
quadrate.L
pterygoid.L
ArtL.SUanL
ANL
PRarL.SPL
dentary
ArtR.SuanR
ANR
PRarR.SPR
45
43
41
49
42
39
40
37
46
51
38
L
Adult
R
quadrate.R
pterygoid.R
VO.PAal
BO.BS.EX.PAoid.EC.ME.SQ.PAtal.
SUoc.LAoid.FR.NA
quadrate.L
pterygoid.L
DE.AN.AR.PRar.SUan.SP
lacrimal.R
jugal.R
quadratojugal.R
quadratojugal.L
jugal.L
PRx.MA
lacrimal.L
19
17
15

Anas crecca

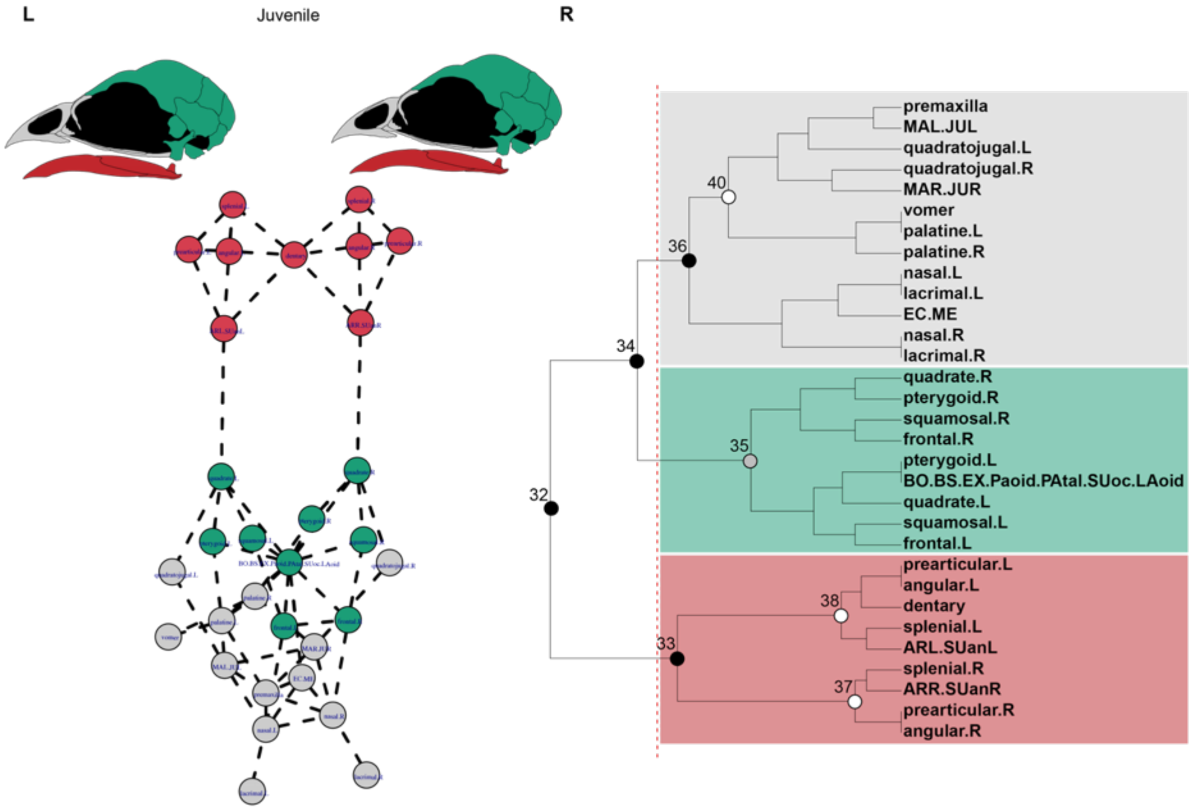
L
Juvenile
R
premaxilla
MAL.JUL
quadratojugal.L
quadratojugal.R
MAR.JUR
vomer
palatine.L
palatine.R
nasal.L
lacrimal.L
EC.ME
nasal.R
lacrimal.R
quadrate.R
pterygoid.R
squamosal.R
frontal.R
pterygoid.L
BO.BS.EX.Paoid.PAtal.SUoc.LAoid
quadrate.L
squamosal.L
frontal.L
prearticular.L
angular.L
dentary
splenial.L
ARL.SUanL
splenial.R
ARR.SUanR
prearticular.R
angular.R
32
33
34
35
36
37
38
40

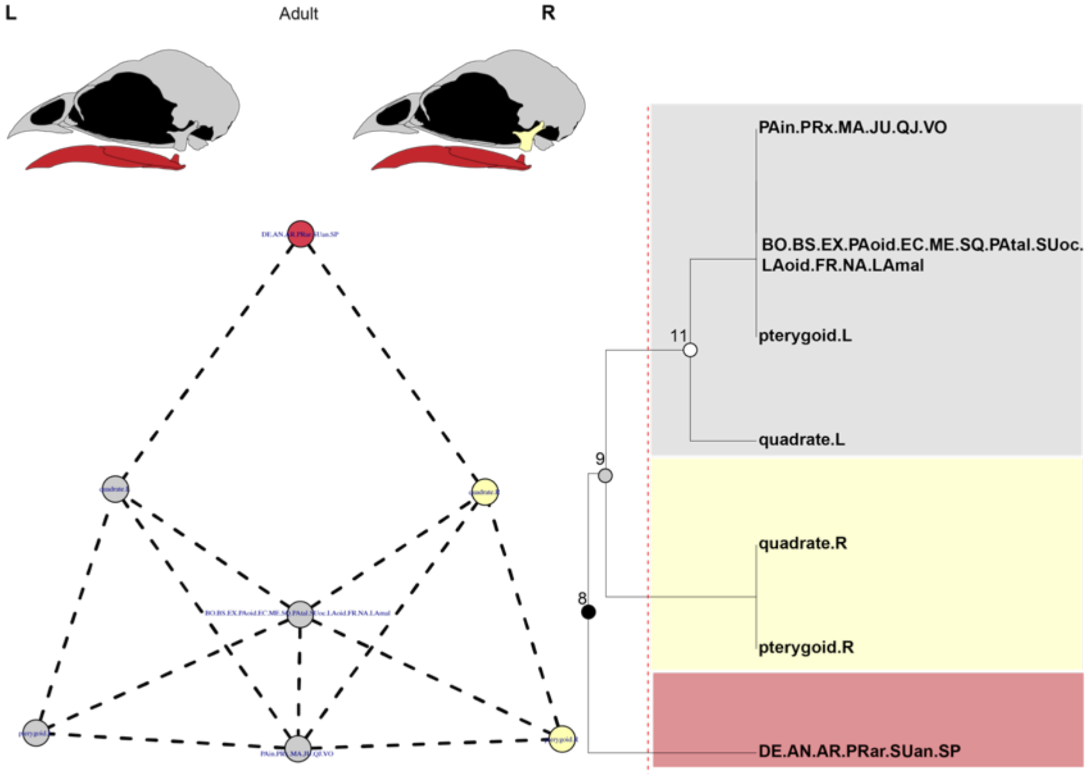
L
Adult
R
PAin.PRx.MA.JU.QJ.VO
BO.BS.EX.PAoid.EC.ME.SQ.PAtal.SUoc.
LAoid.FR.NA.LAmal
pterygoid.L
quadrate.L
quadrate.R
pterygoid.R
DE.AN.AR.PRar.SUan.SP
8
9
11

Anser anser

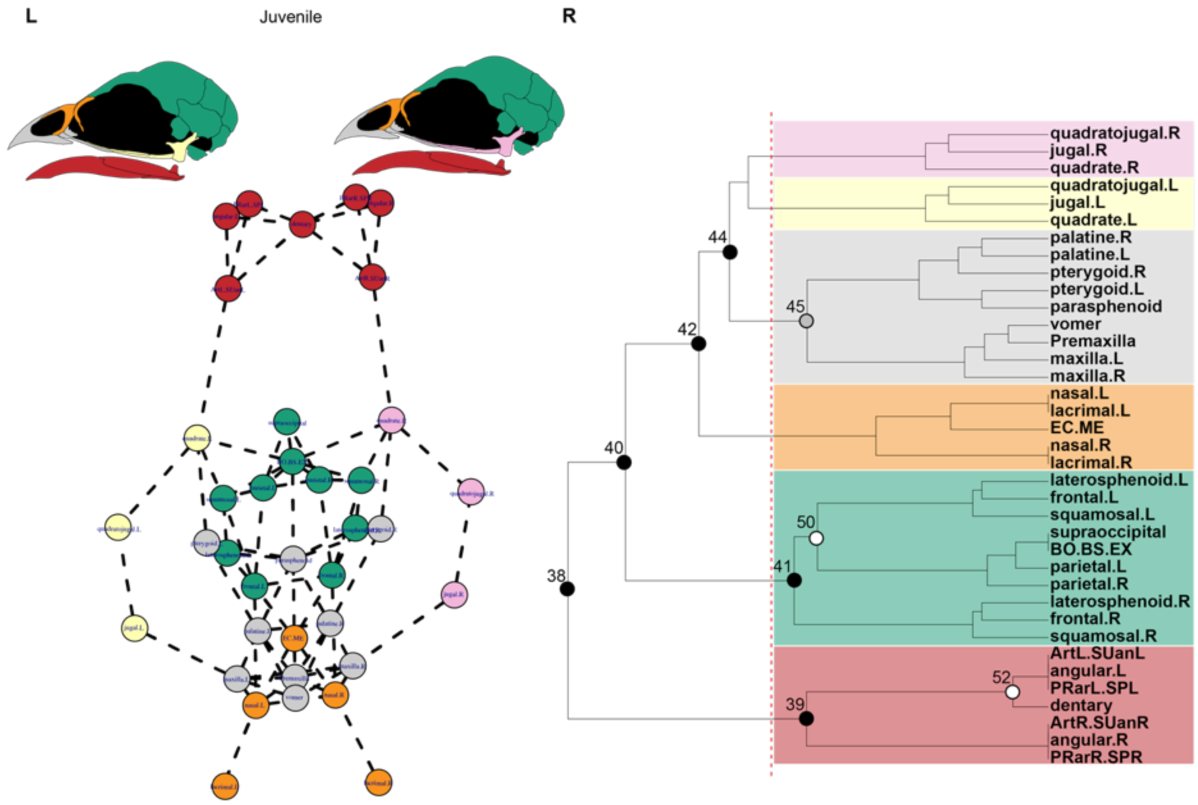
L
Juvenile
R
quadratojugal.R
jugal.R
quadrate.R
quadratojugal.L
jugal.L
quadrate.L
palatine.R
palatine.L
pterygoid.R
pterygoid.L
parasphenoid
vomer
Premaxilla
maxilla.L
maxilla.R
nasal.L
lacrimal.L
EC.ME
nasal.R
lacrimal.R
laterosphenoid.L
frontal.L
squamosal.L
supraoccipital
BO.BS.EX
parietal.L
parietal.R
laterosphenoid.R
frontal.R
squamosal.R
ArtL.SUanL
angular.L
PRarL.SPL
dentary
ArtR.SUanR
angular.R
PRarR.SPR
44
45
42
40
50
41
38
52
39

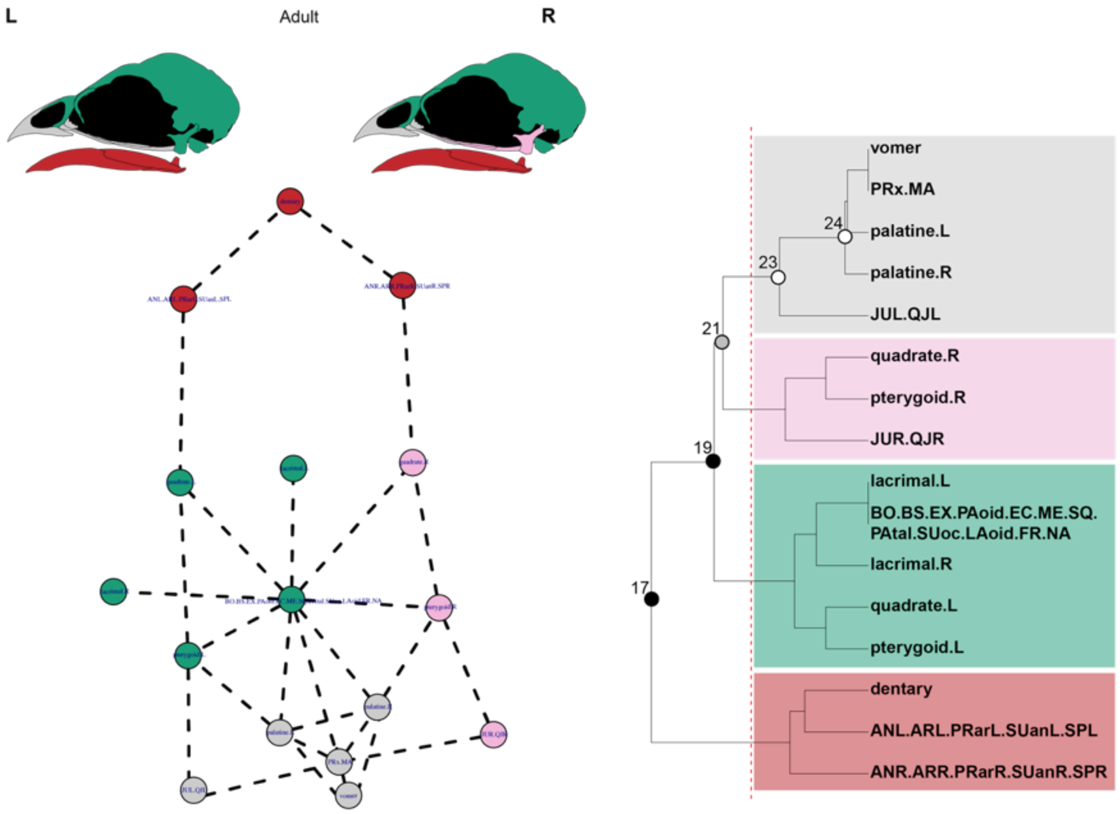
L
Adult
R
vomer
PRx.MA
palatine.L
palatine.R
JUL.QJL
quadrate.R
pterygoid.R
JUR.QJR
lacrimal.L
BO.BS.EX.PAoid.EC.ME.SQ.
PAtal.SUoc.LAoid.FR.NA
lacrimal.R
quadrate.L
pterygoid.L
dentary
ANL.ARL.PRarL.SUanL.SPL
ANR.ARR.PRarR.SUanR.SPR
24
23
21
19
17

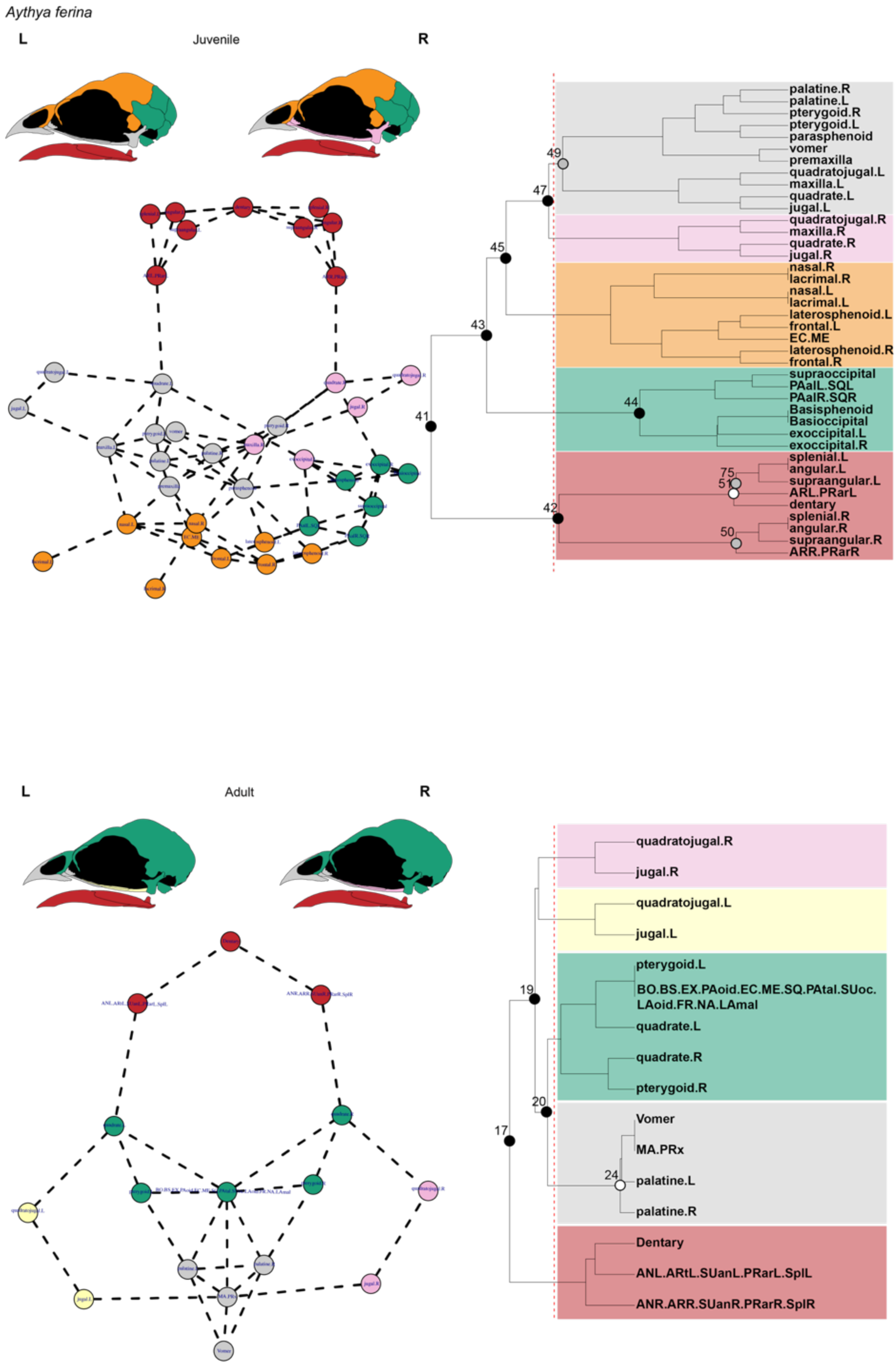

Aythya ferina
L
Juvenile
R
49
47
45
43
44
41
42
75
51
50
palatine.R
palatine.L
pterygoid.R
pterygoid.L
parasphenoid
vomer
premaxilla
quadratojugal.L
maxilla.L
quadrate.L
jugal.L
quadratojugal.R
maxilla.R
quadrate.R
jugal.R
nasal.R
lacrimal.R
nasal.L
lacrimal.L
laterosphenoid.L
frontal.L
EC.ME
laterosphenoid.R
frontal.R
supraoccipital
PAalL.SQL
PAalR.SQR
Basisphenoid
Basioccipital
exoccipital.L
exoccipital.R
splenial.L
angular.L
supraangular.L
ARL.PRarL
dentary
splenial.R
angular.R
supraangular.R
ARR.PRarR
L
Adult
R
19
20
17
24
quadratojugal.R
jugal.R
quadratojugal.L
jugal.L
pterygoid.L
BO.BS.EX.PAoid.EC.ME.SQ.PAtal.SUoc.
LAoid.FR.NA.LAmal
quadrate.L
quadrate.R
pterygoid.R
Vomer
MA.PRx
palatine.L
palatine.R
Dentary
ANL.ARtL.SUanL.PRarL.SplL
ANR.ARR.SUanR.PRarR.SplR

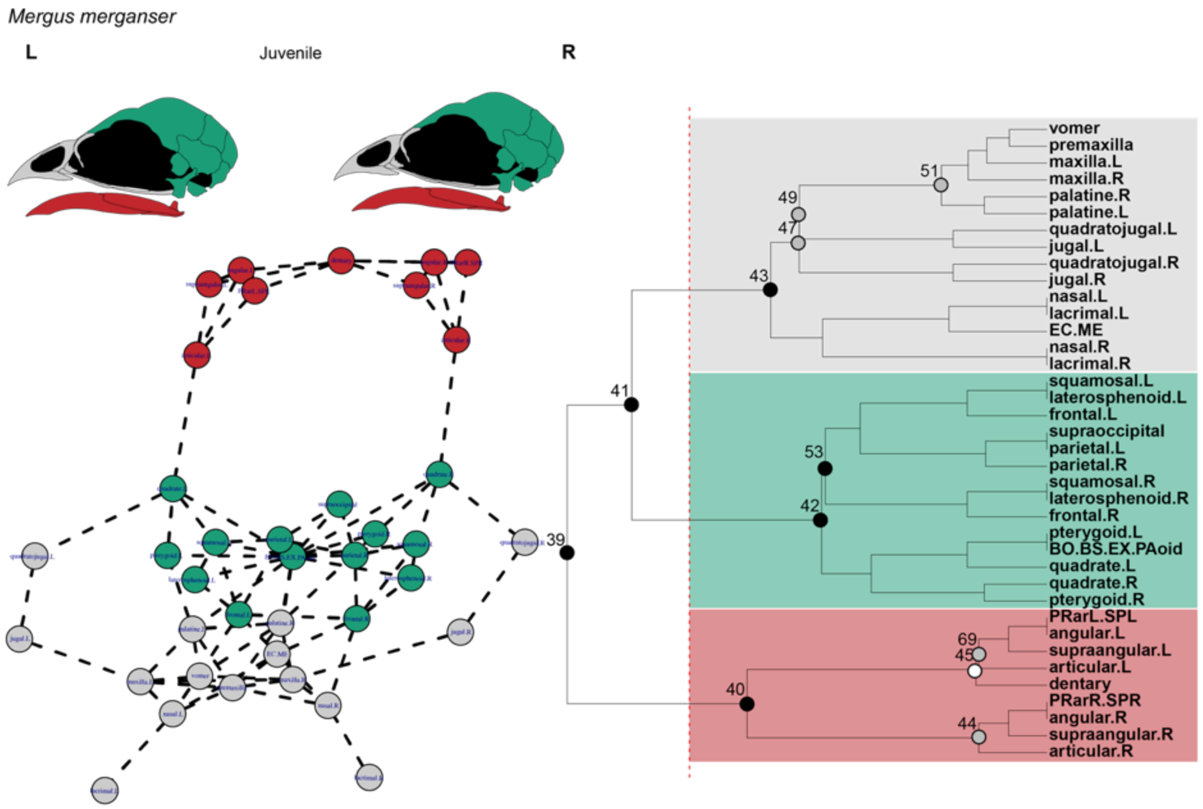
Mergus merganser
L
Juvenile
R
vomer
premaxilla
maxilla.L
maxilla.R
palatine.R
palatine.L
quadratojugal.L
jugal.L
quadratojugal.R
jugal.R
nasal.L
lacrimal.L
EC.ME
nasal.R
lacrimal.R
squamosal.L
laterosphenoid.L
frontal.L
supraoccipital
parietal.L
parietal.R
squamosal.R
laterosphenoid.R
frontal.R
pterygoid.L
BO.BS.EX.PAoid
quadrate.L
quadrate.R
pterygoid.R
PRarL.SPL
angular.L
supraangular.L
articular.L
dentary
PRarR.SPR
angular.R
supraangular.R
articular.R
51
49
47
43
41
53
42
39
69
45
40
44

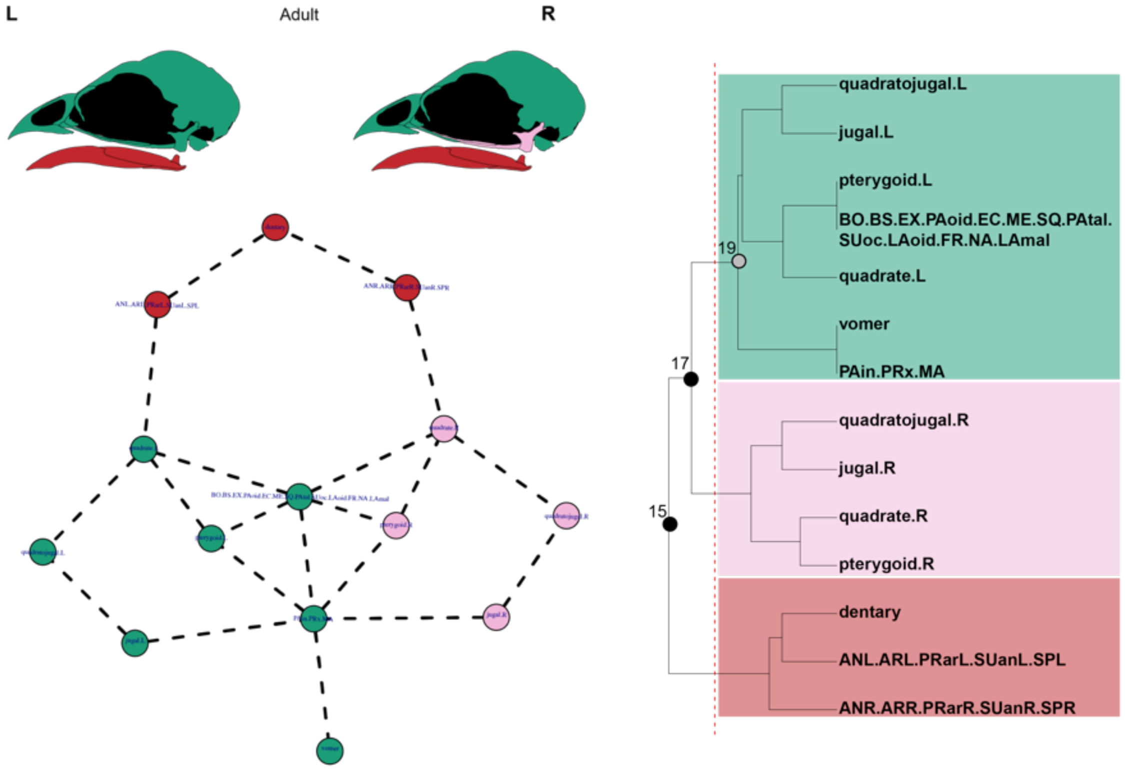
L
Adult
R
quadratojugal.L
jugal.L
pterygoid.L
BO.BS.EX.PAoid.EC.ME.SQ.PAtal.
SUoc.LAoid.FR.NA.LAmal
quadrate.L
vomer
PAin.PRx.MA
quadratojugal.R
jugal.R
quadrate.R
pterygoid.R
dentary
ANL.ARL.PRarL.SUanL.SPL
ANR.ARR.PRarR.SUanR.SPR
19
17
15

Cygnus olor

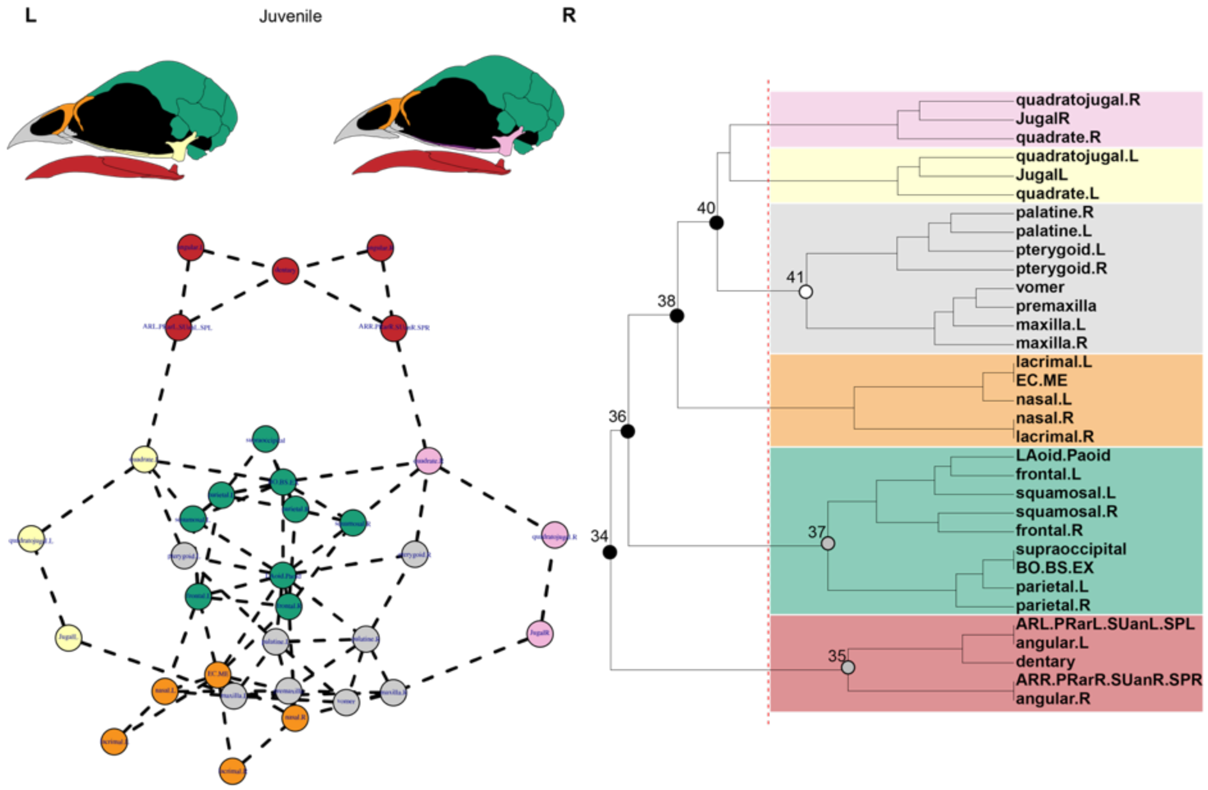
L
Juvenile
R
quadratojugal.R
JugalR
quadrate.R
quadratojugal.L
JugalL
quadrate.L
palatine.R
palatine.L
pterygoid.L
pterygoid.R
vomer
premaxilla
maxilla.L
maxilla.R
lacrimal.L
EC.ME
nasal.L
nasal.R
lacrimal.R
LAoid.Paoid
frontal.L
squamosal.L
squamosal.R
frontal.R
supraoccipital
BO.BS.EX
parietal.L
parietal.R
ARL.PRarL.SUanL.SPL
angular.L
dentary
ARR.PRarR.SUanR.SPR
angular.R
40
41
38
36
37
34
35

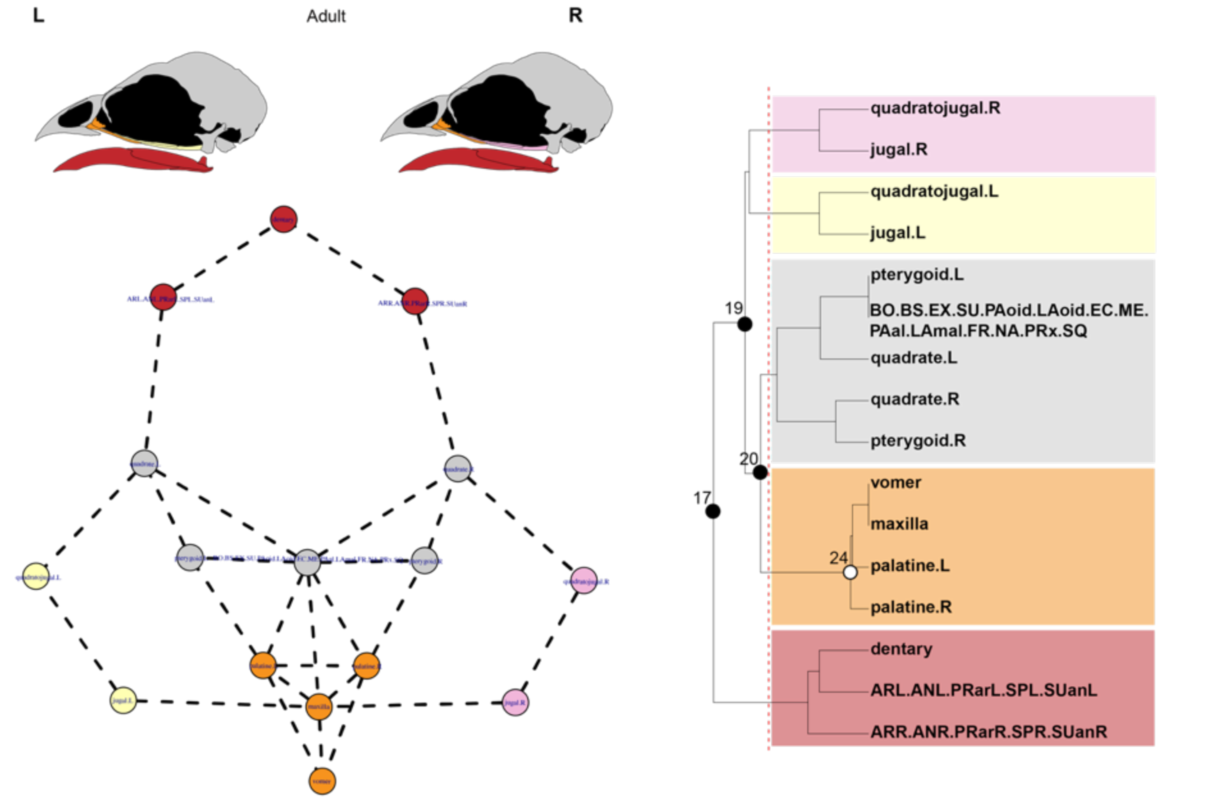
L
Adult
R
quadratojugal.R
jugal.R
quadratojugal.L
jugal.L
pterygoid.L
BO.BS.EX.SU.PAoid.LAoid.EC.ME.
PAal.LAmal.FR.NA.PRx.SQ
quadrate.L
quadrate.R
pterygoid.R
vomer
maxilla
palatine.L
palatine.R
dentary
ARL.ANL.PRarL.SPL.SUanL
ARR.ANR.PRarR.SPR.SUanR
19
20
17
24

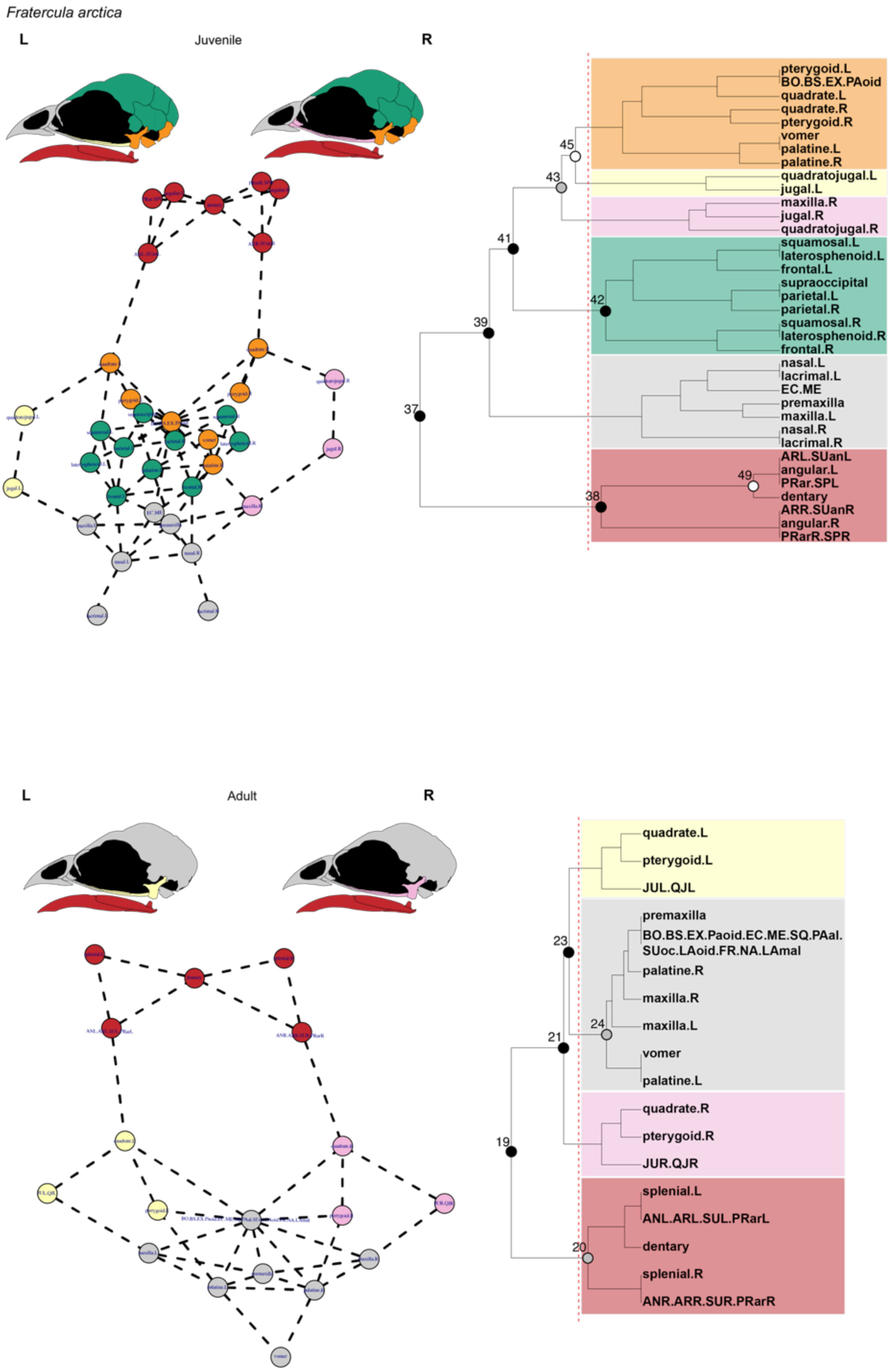

Fratercula arctica
L
Juvenile
R
45
43
41
39
37
42
49
38
pterygoid.L
BO.BS.EX.PAoid
quadrate.L
quadrate.R
pterygoid.R
vomer
palatine.L
palatine.R
quadratojugal.L
jugal.L
maxilla.R
jugal.R
quadratojugal.R
squamosal.L
laterosphenoid.L
frontal.L
supraoccipital
parietal.L
parietal.R
squamosal.R
laterosphenoid.R
frontal.R
nasal.L
lacrimal.L
EC.ME
premaxilla
maxilla.L
nasal.R
lacrimal.R
ARL.SUanL
angular.L
PRar.SPL
dentary
ARR.SUanR
angular.R
PRarR.SPR
L
Adult
R
23
24
21
19
20
quadrate.L
pterygoid.L
JUL.QJL
premaxilla
BO.BS.EX.Paoid.EC.ME.SQ.PAal.
SUoc.LAoid.FR.NA.LAmal
palatine.R
maxilla.R
maxilla.L
vomer
palatine.L
quadrate.R
pterygoid.R
JUR.QJR
splenial.L
ANL.ARL.SUL.PRarL
dentary
splenial.R
ANR.ARR.SUR.PRarR

Ptychoramphus aleuticus

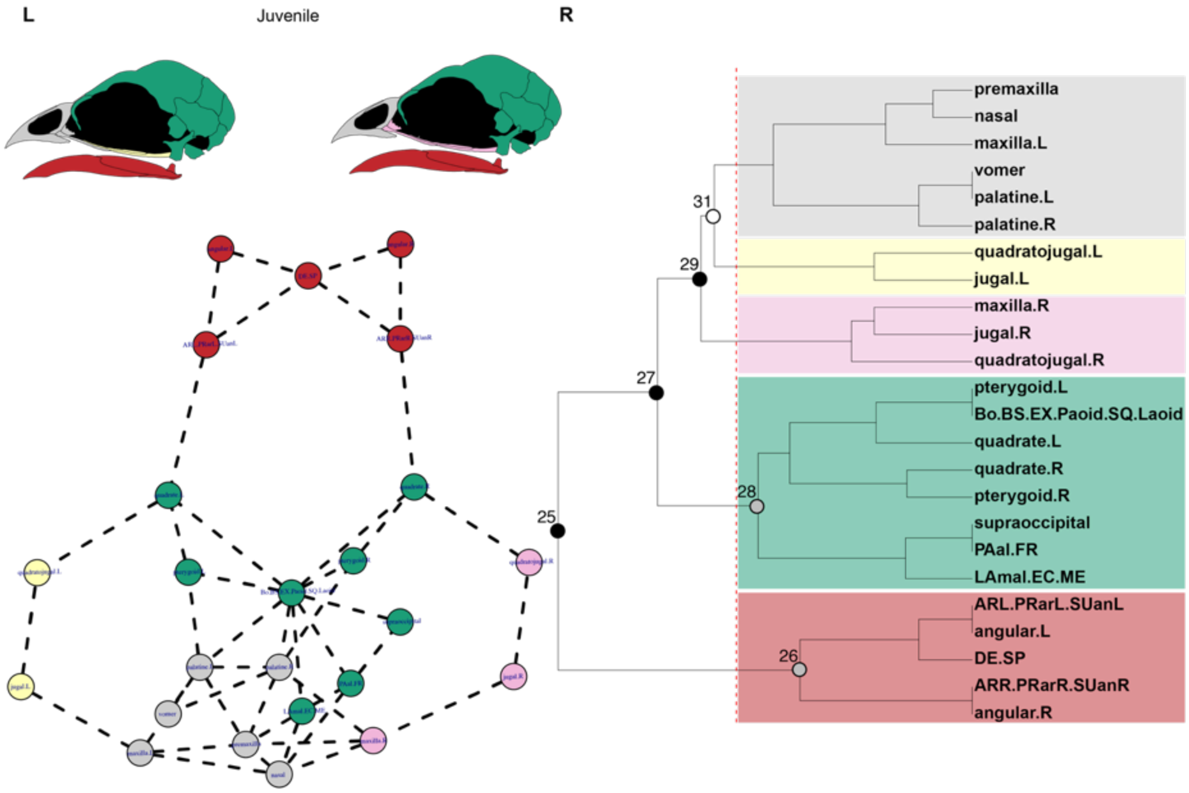

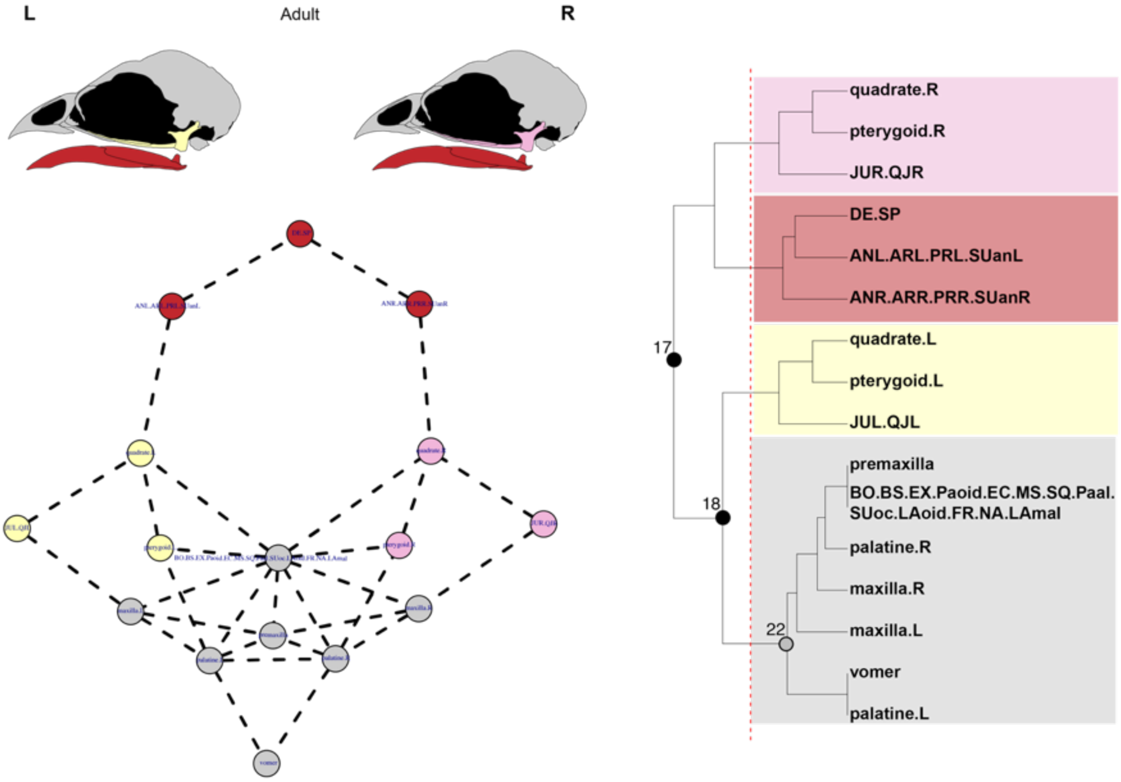

Recurvirostra avosetta

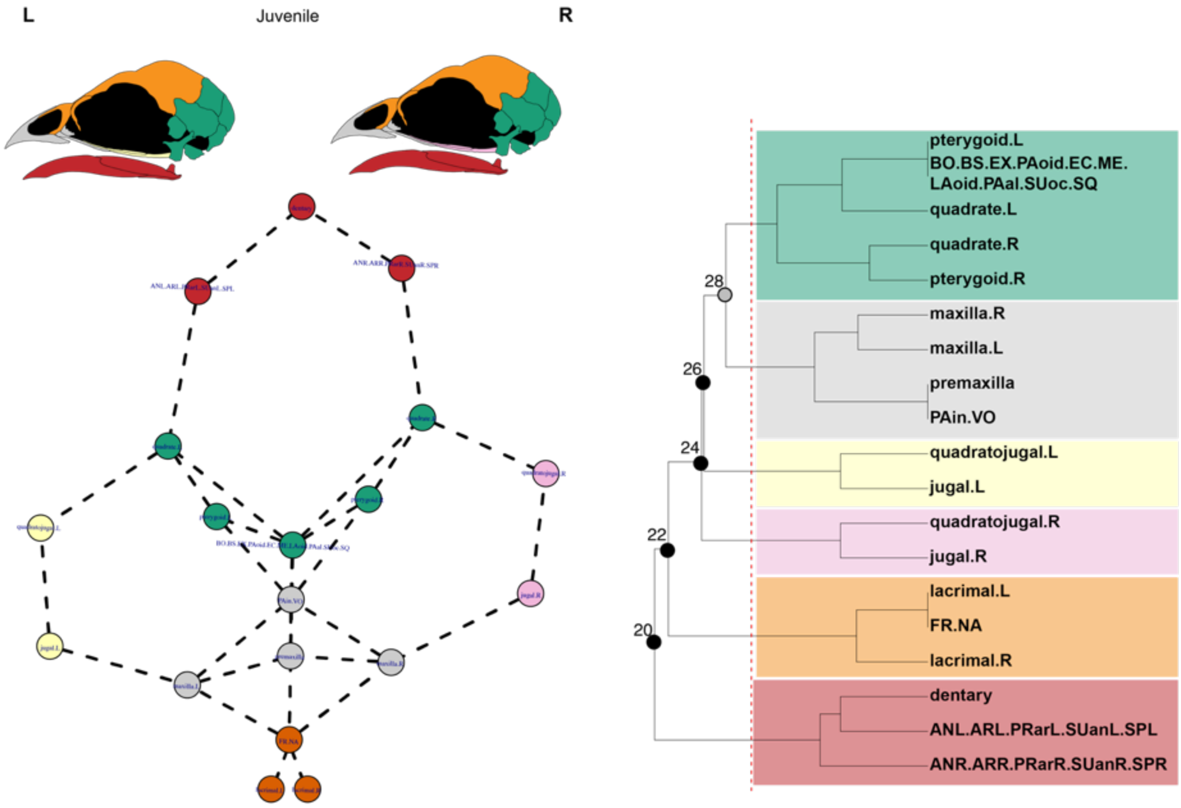

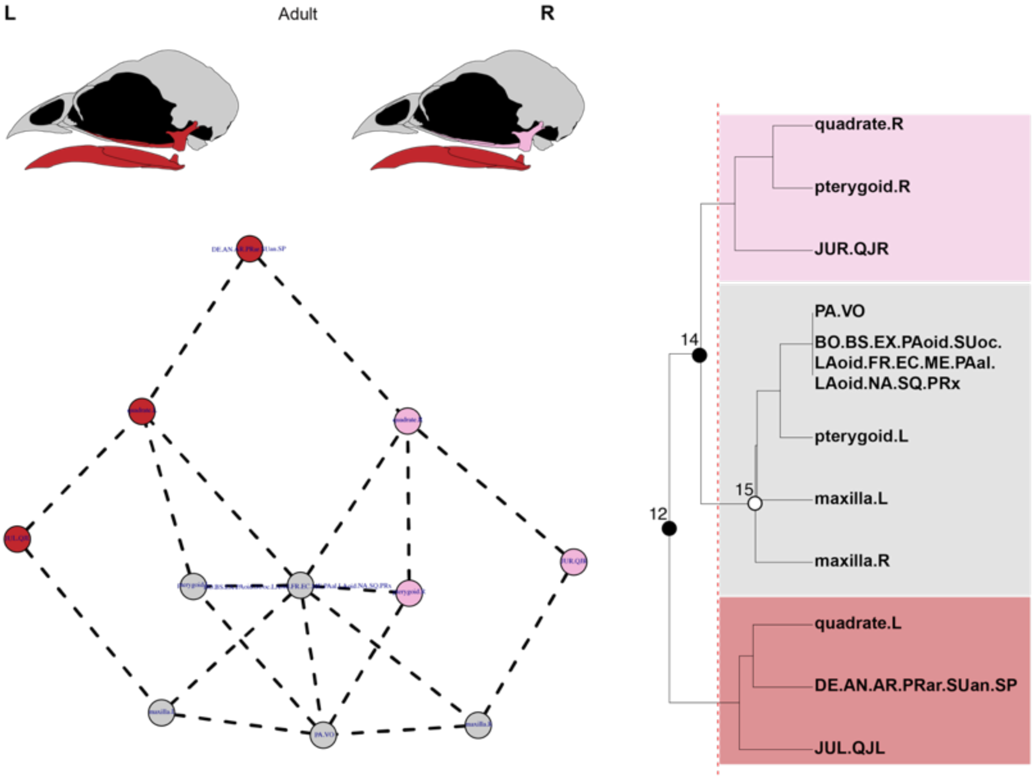

Larus ridubundus

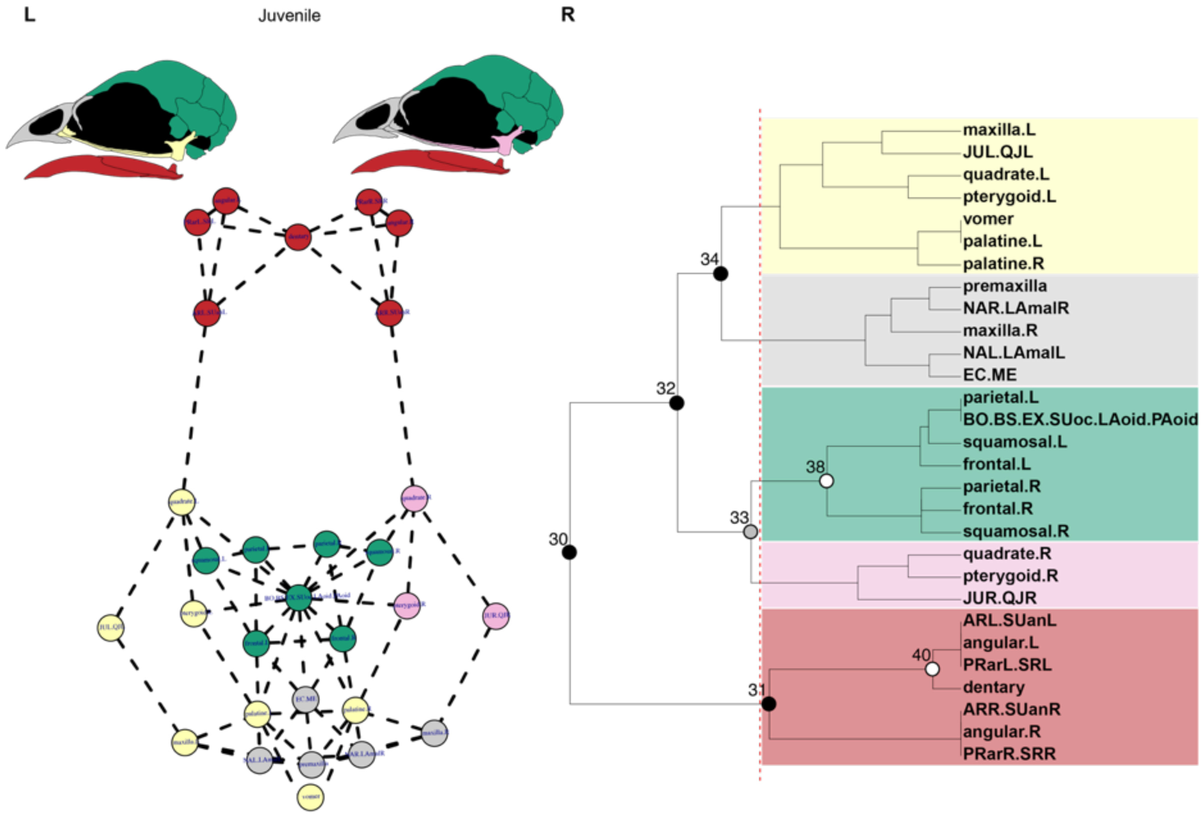

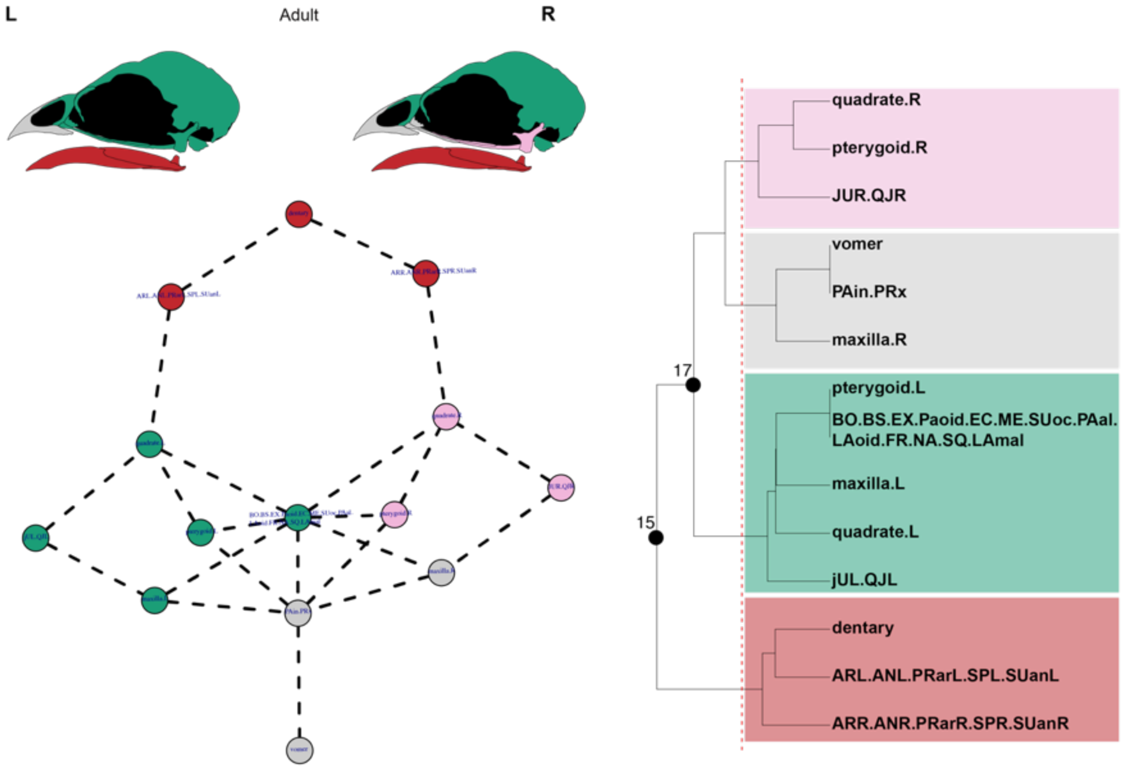

Ciconia ciconia

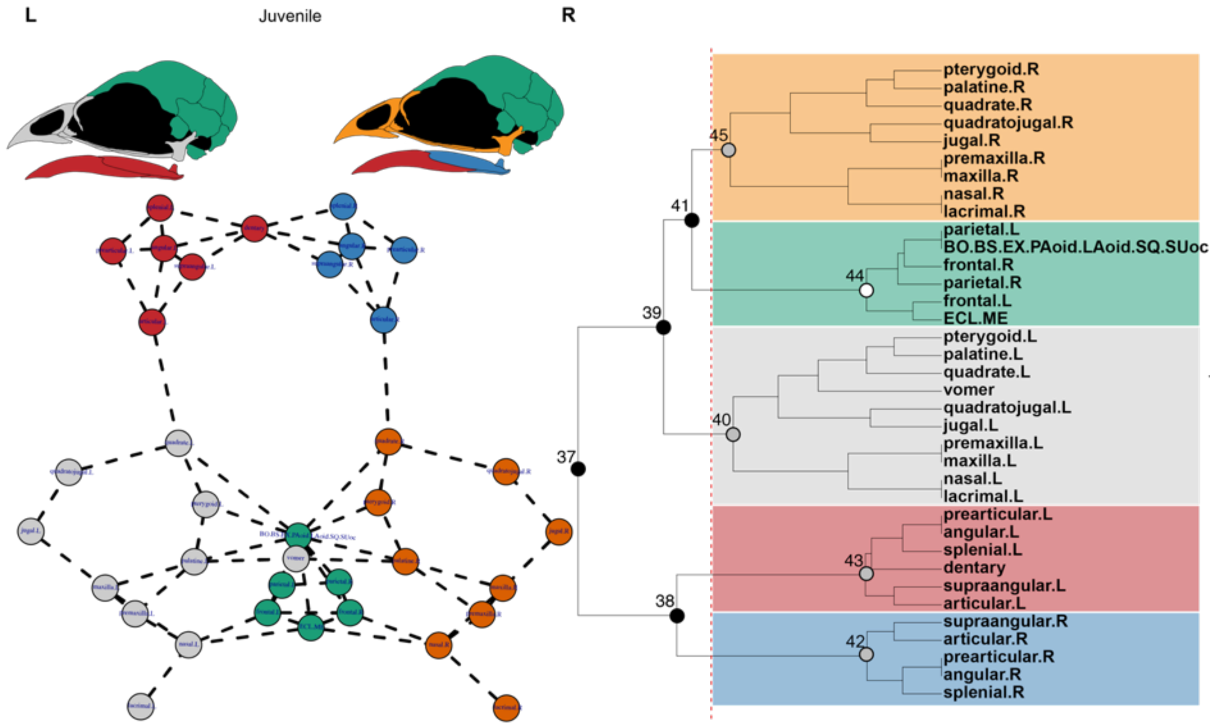

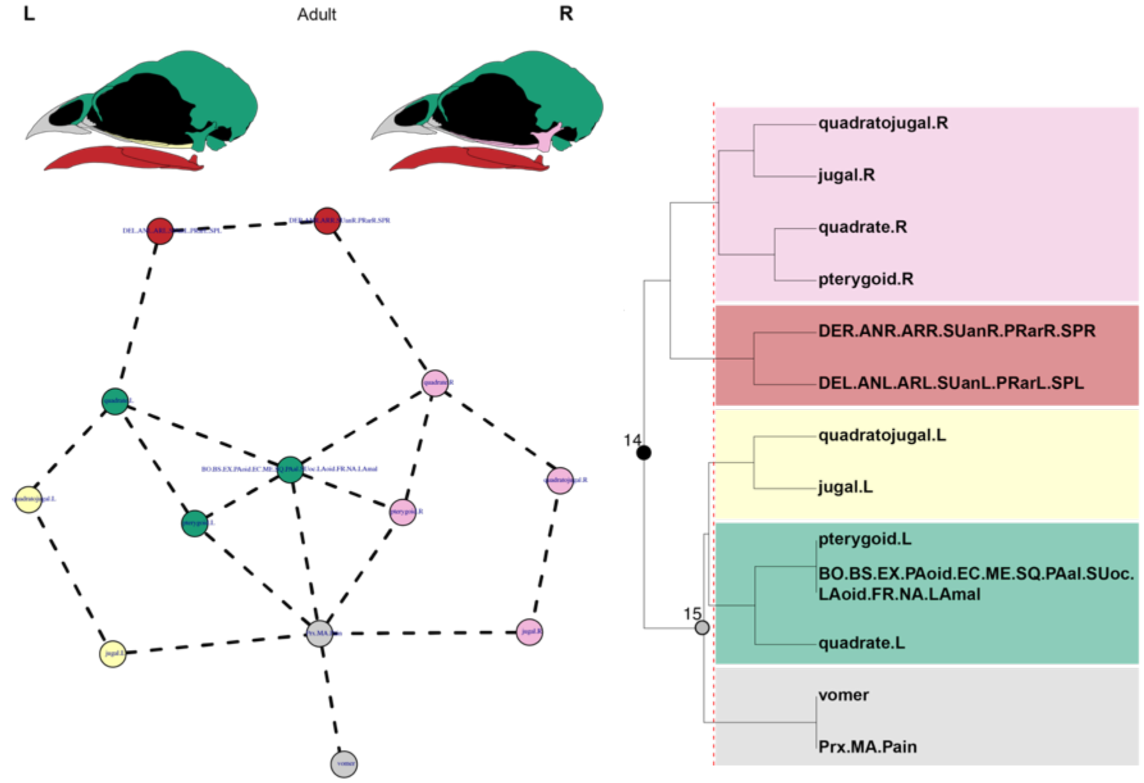

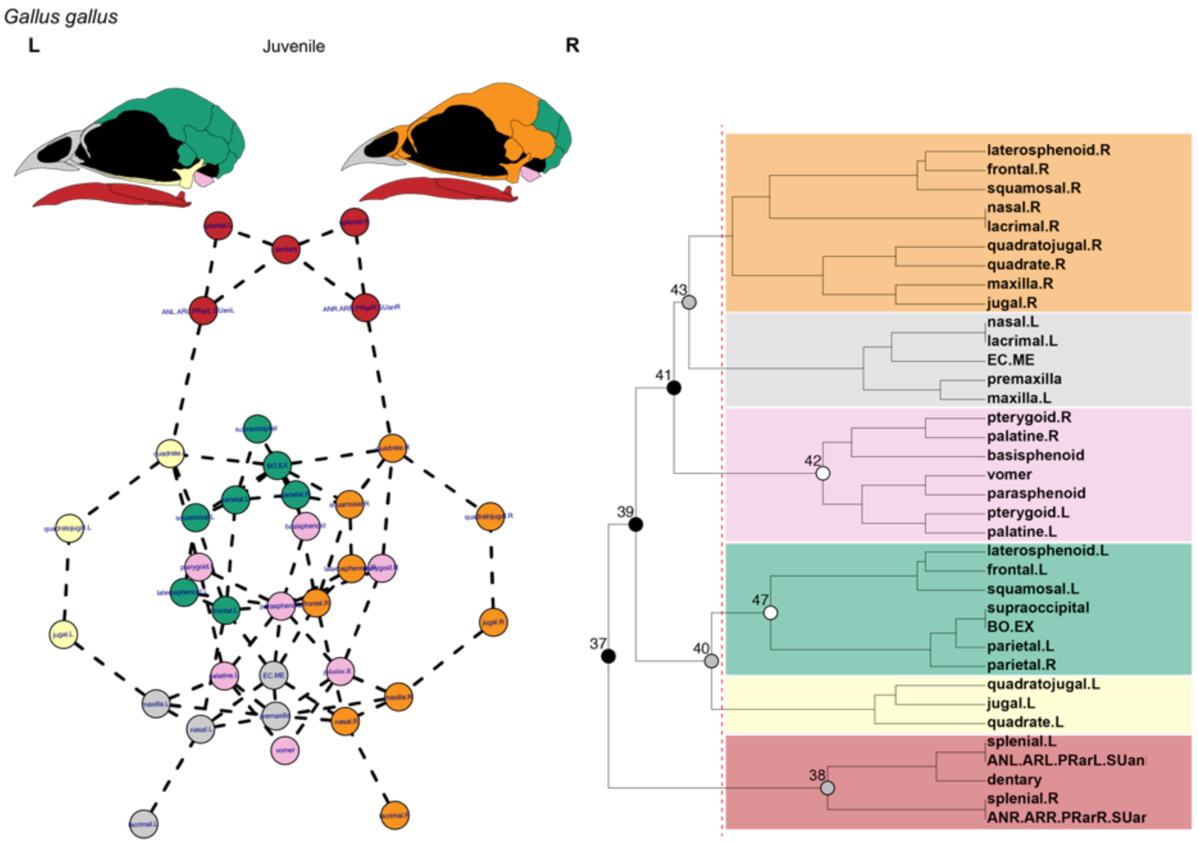
Gallus gallus
L
Juvenile
R
laterosphenoid.R
frontal.R
squamosal.R
nasal.R
lacrimal.R
quadratojugal.R
quadrate.R
maxilla.R
jugal.R
nasal.L
lacrimal.L
EC.ME
premaxilla
maxilla.L
pterygoid.R
palatine.R
basisphenoid
vomer
parasphenoid
pterygoid.L
palatine.L
laterosphenoid.L
frontal.L
squamosal.L
supraoccipital
BO.EX
parietal.L
parietal.R
quadratojugal.L
jugal.L
quadrate.L
splenial.L
ANL.ARL.PRarL.SUan
dentary
splenial.R
ANR.ARR.PRarR.SUar
43
41
42
39
47
37
40
38

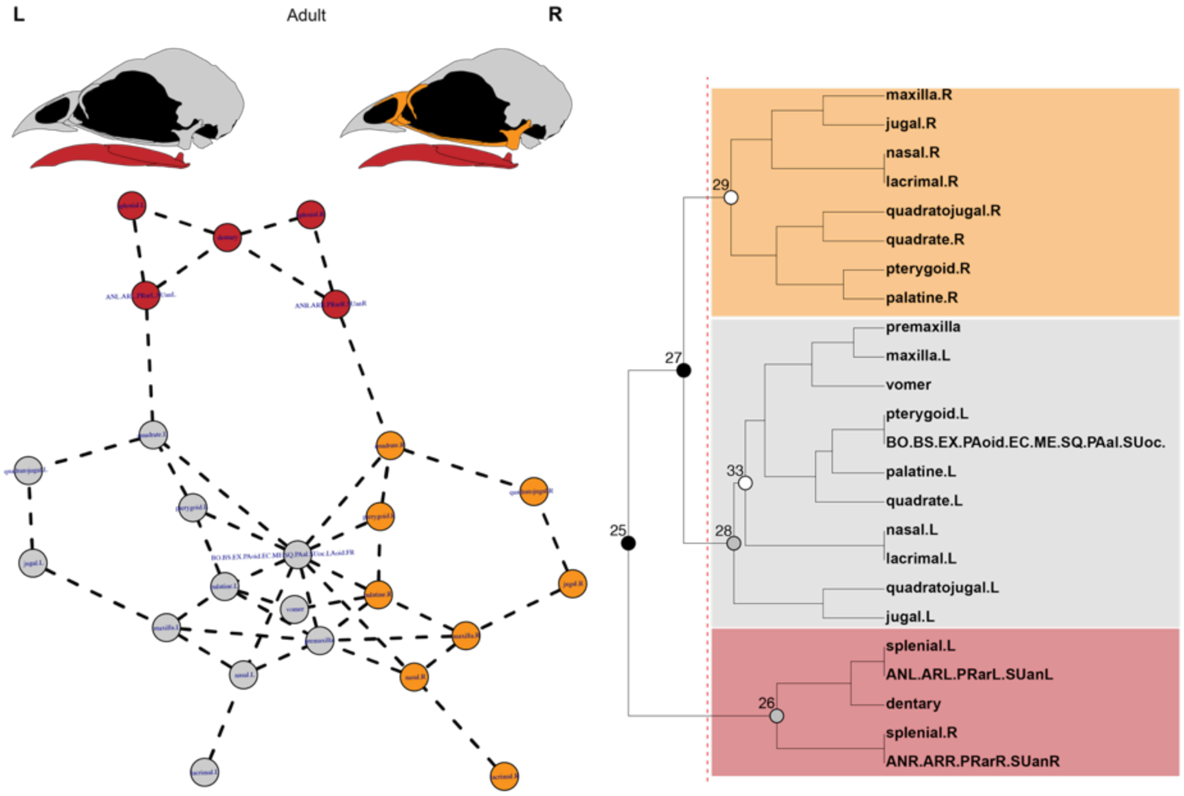
L
Adult
R
maxilla.R
jugal.R
nasal.R
lacrimal.R
quadratojugal.R
quadrate.R
pterygoid.R
palatine.R
premaxilla
maxilla.L
vomer
pterygoid.L
BO.BS.EX.PAoid.EC.ME.SQ.PAal.SUoc.
palatine.L
quadrate.L
nasal.L
lacrimal.L
quadratojugal.L
jugal.L
splenial.L
ANL.ARL.PRarL.SUanL
dentary
splenial.R
ANR.ARR.PRarR.SUanR
29
27
33
25
28
26

Grus paradisea

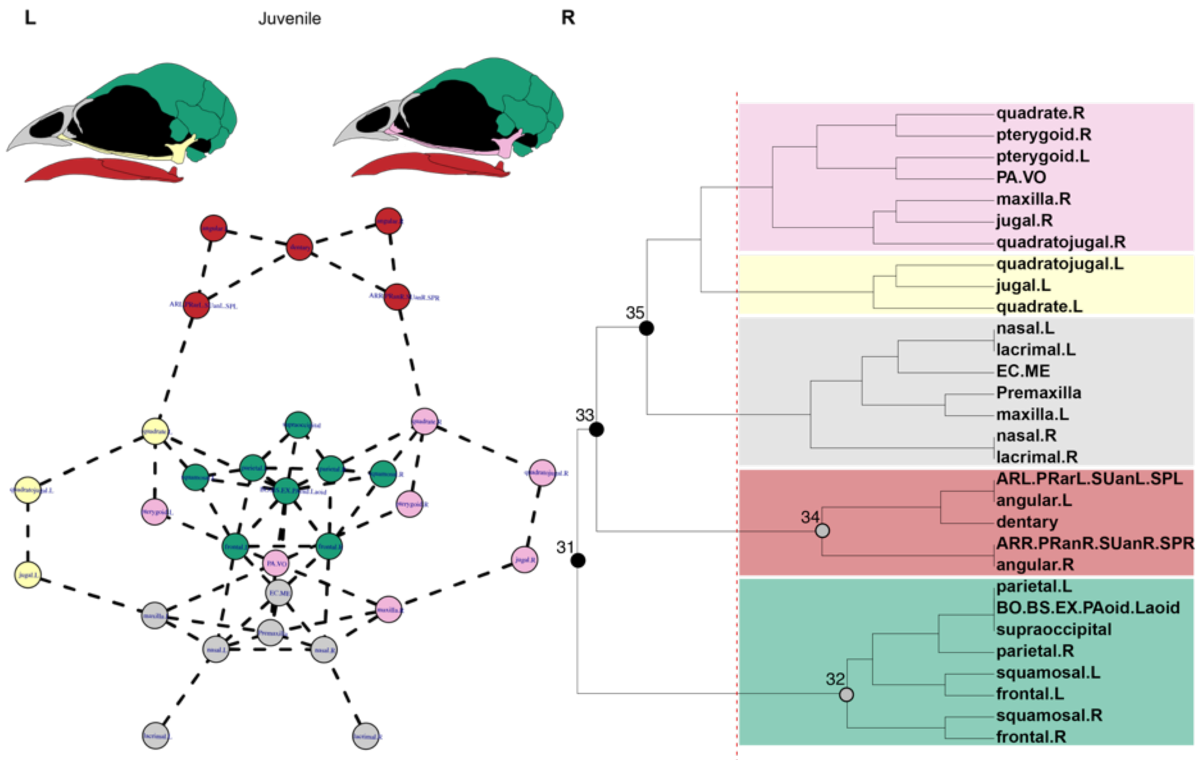

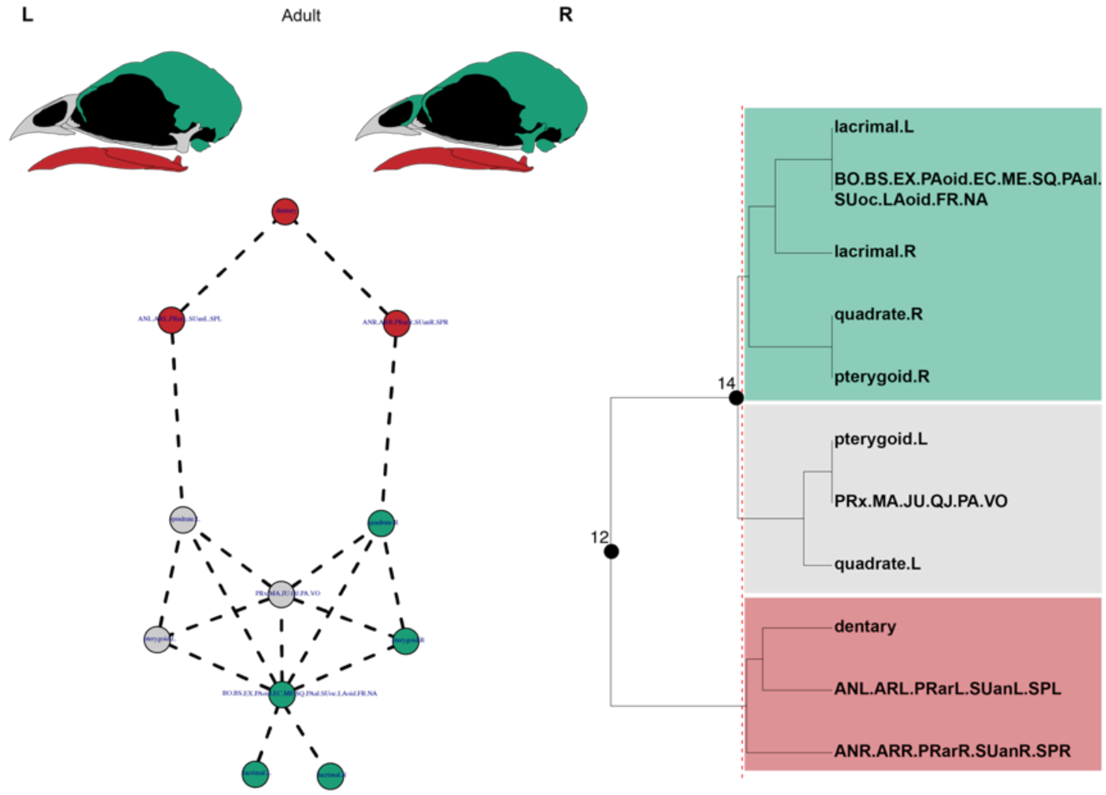

Grus japonensis

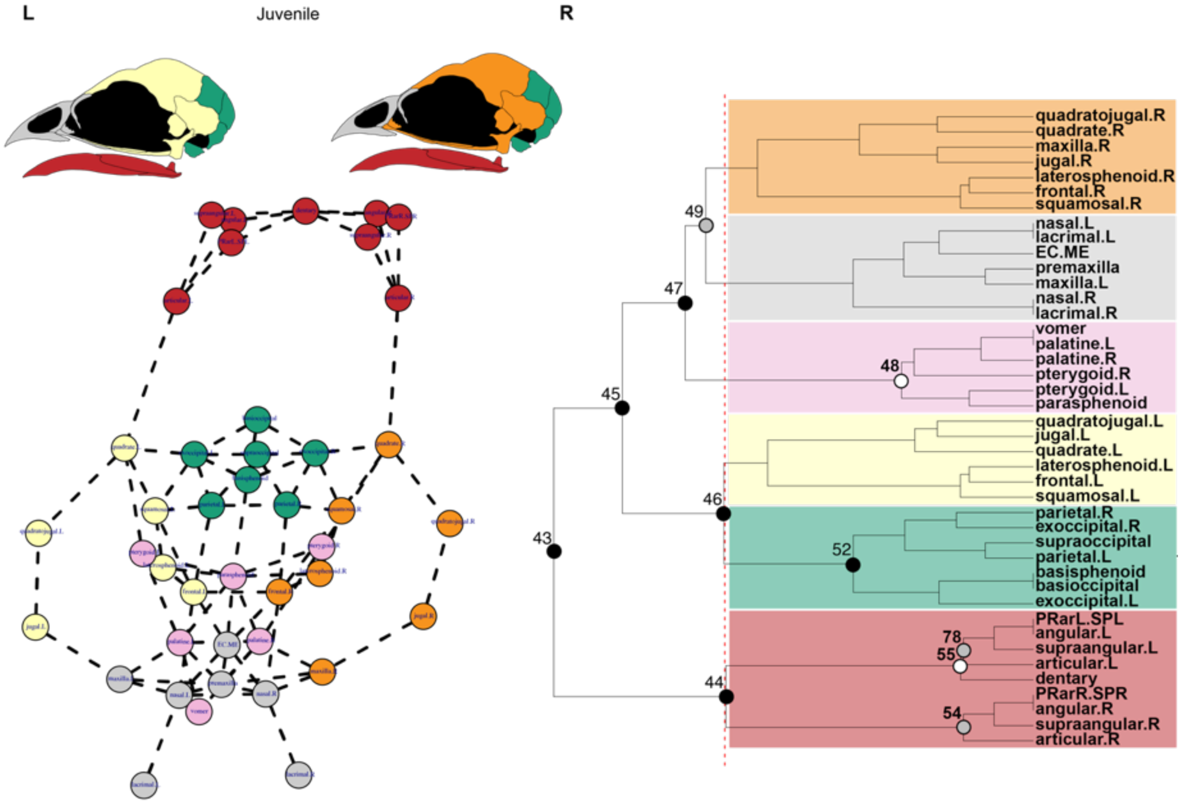

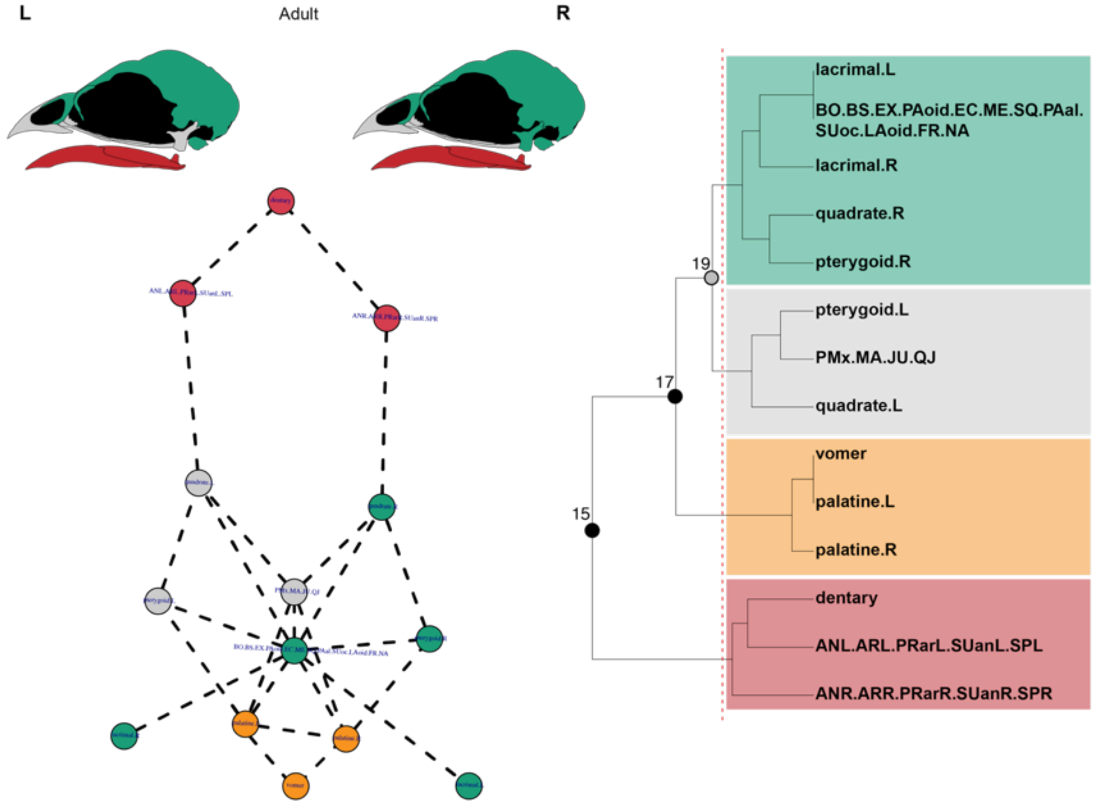

Otis tardas

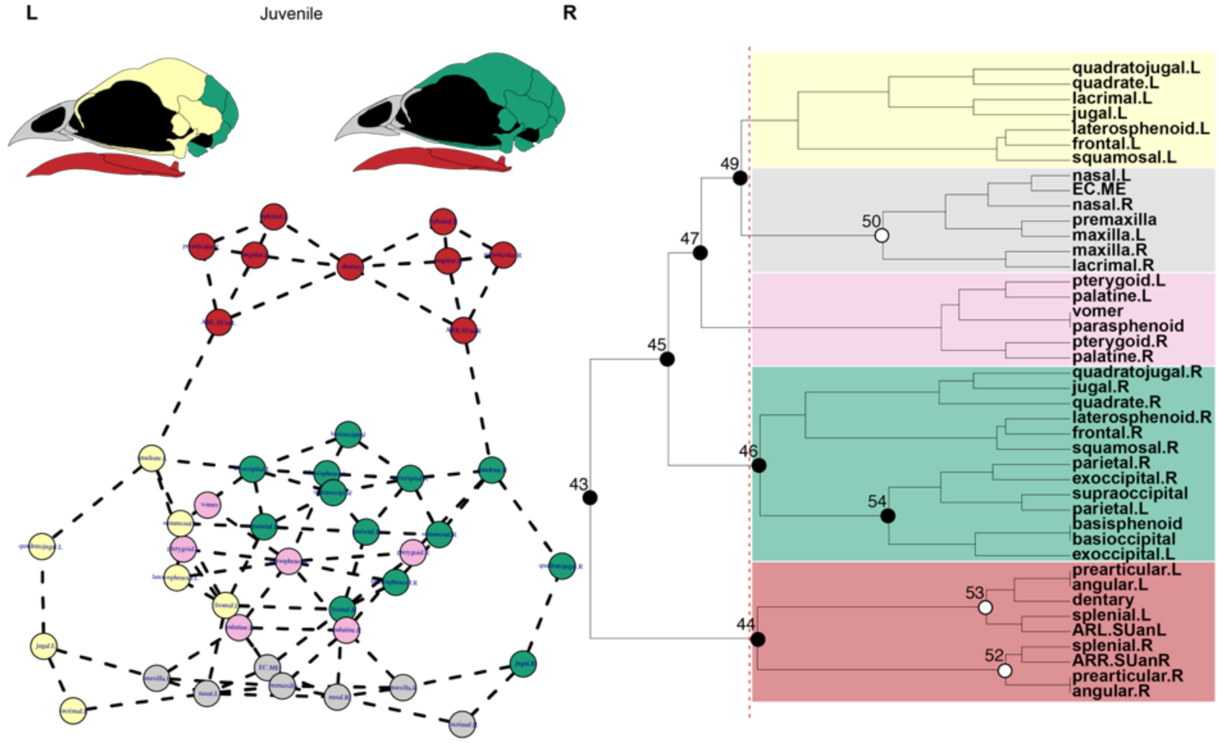

L
Juvenile
R
quadratojugal.L
quadrate.L
lacrimal.L
jugal.L
laterosphenoid.L
frontal.L
squamosal.L
nasal.L
EC.ME
nasal.R
premaxilla
maxilla.L
maxilla.R
lacrimal.R
pterygoid.L
palatine.L
vomer
parasphenoid
pterygoid.R
palatine.R
quadratojugal.R
jugal.R
quadrate.R
laterosphenoid.R
frontal.R
squamosal.R
parietal.R
exoccipital.R
supraoccipital
parietal.L
basisphenoid
basioccipital
exoccipital.L
prearticular.L
angular.L
dentary
splenial.L
ARL.SUanL
splenial.R
ARR.SUanR
prearticular.R
angular.R
43
44
45
46
47
49
50
52
53
54

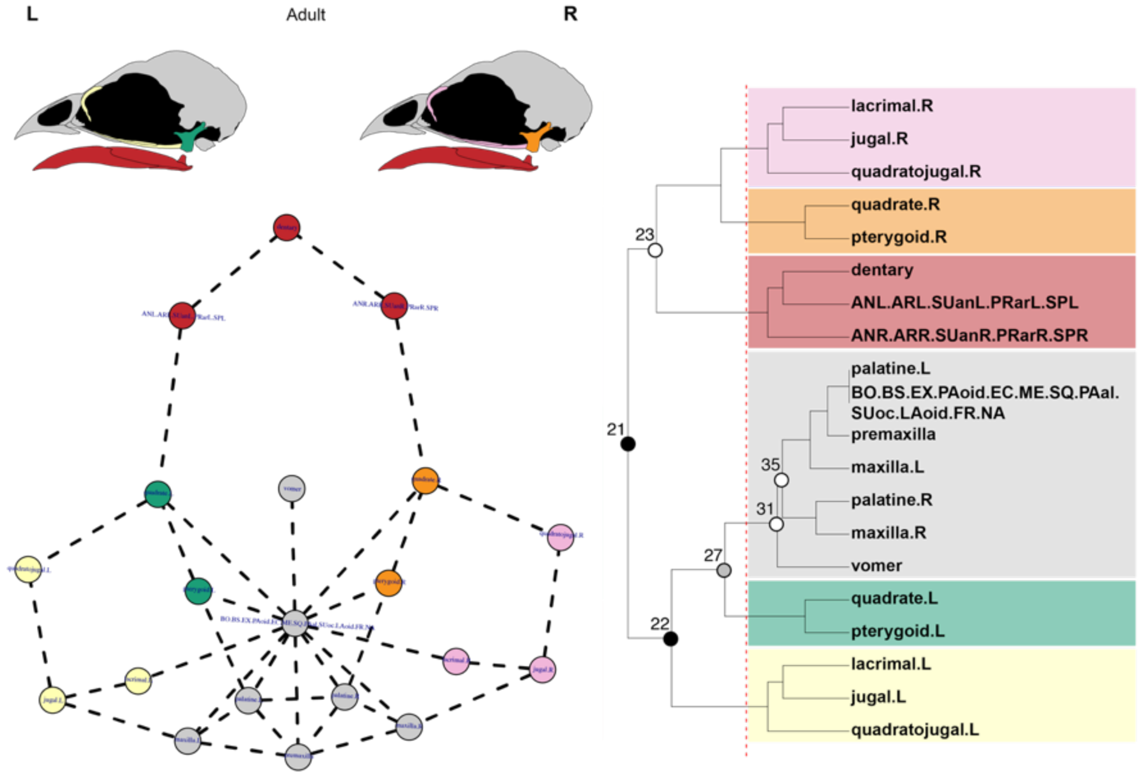

L
Adult
R
lacrimal.R
jugal.R
quadratojugal.R
quadrate.R
pterygoid.R
dentary
ANL.ARL.SUanL.PRarL.SPL
ANR.ARR.SUanR.PRarR.SPR
palatine.L
BO.BS.EX.PAoid.EC.ME.SQ.PAal.
SUoc.LAoid.FR.NA
premaxilla
maxilla.L
palatine.R
maxilla.R
vomer
quadrate.L
pterygoid.L
lacrimal.L
jugal.L
quadratojugal.L
21
22
23
27
31
35

Rhea americana

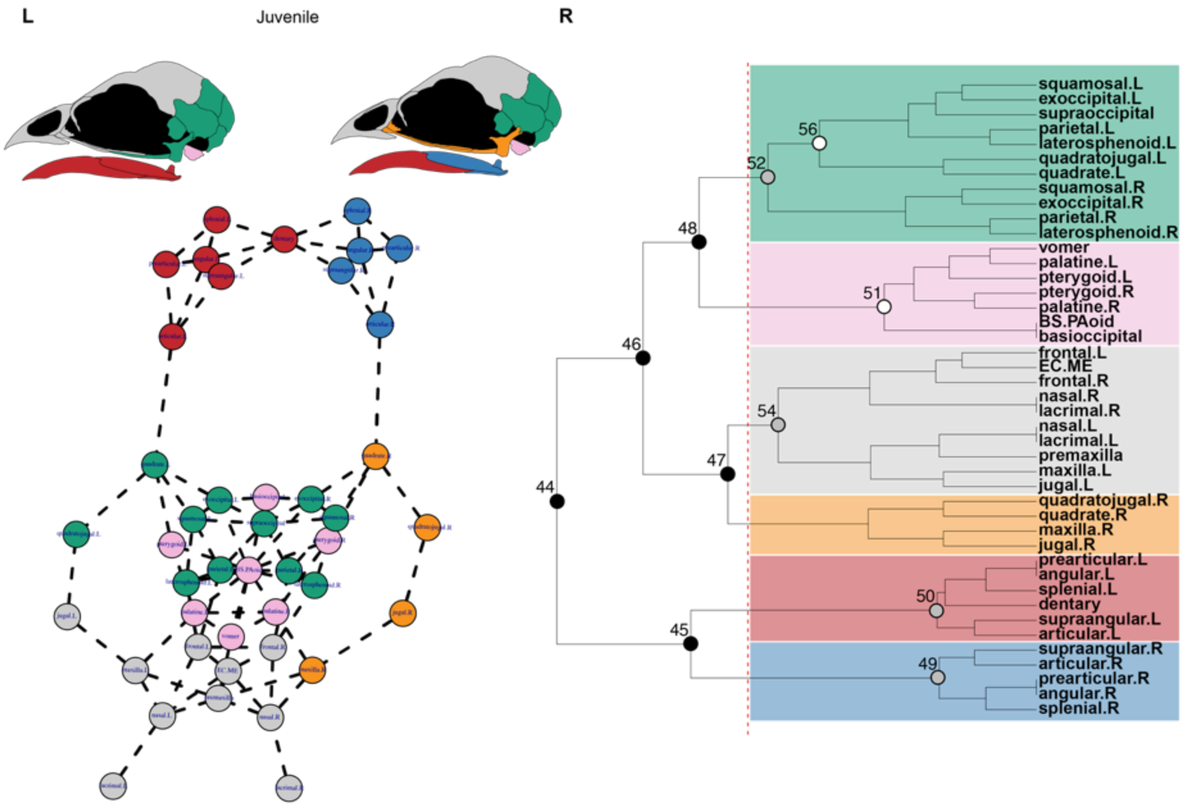

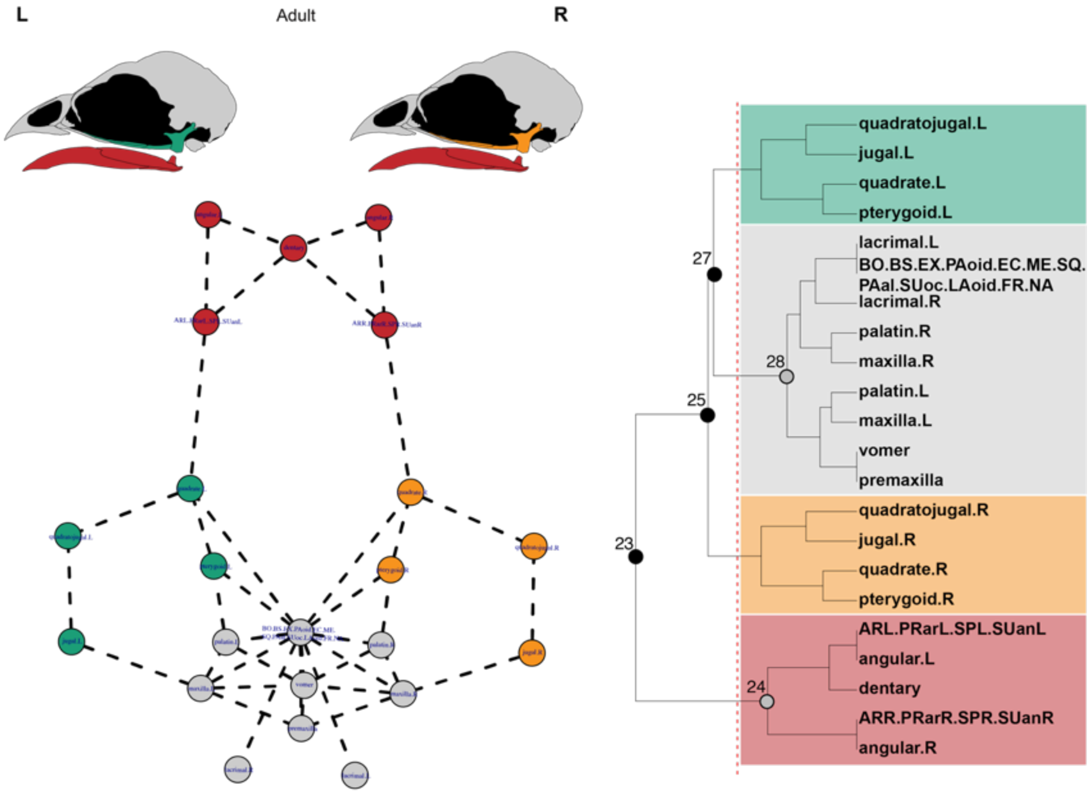

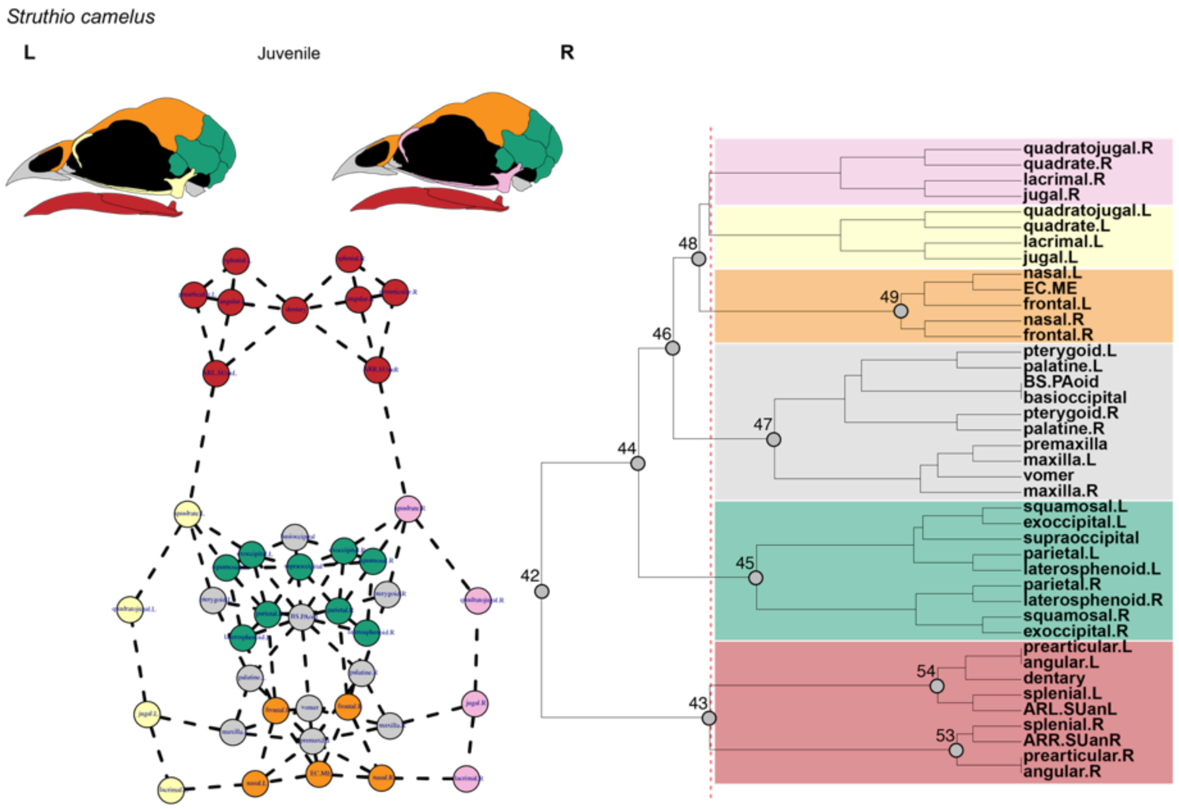

Struthio camelus
L
Juvenile
R
48
49
46
47
44
45
42
54
43
53
quadratojugal.R
quadrate.R
lacrimal.R
jugal.R
quadratojugal.L
quadrate.L
lacrimal.L
jugal.L
nasal.L
EC.ME
frontal.L
nasal.R
frontal.R
pterygoid.L
palatine.L
BS.PAoid
basioccipital
pterygoid.R
palatine.R
premaxilla
maxilla.L
vomer
maxilla.R
squamosal.L
exoccipital.L
supraoccipital
parietal.L
laterosphenoid.L
parietal.R
laterosphenoid.R
squamosal.R
exoccipital.R
prearticular.L
angular.L
dentary
splenial.L
ARL.SUanL
splenial.R
ARR.SUanR
prearticular.R
angular.R

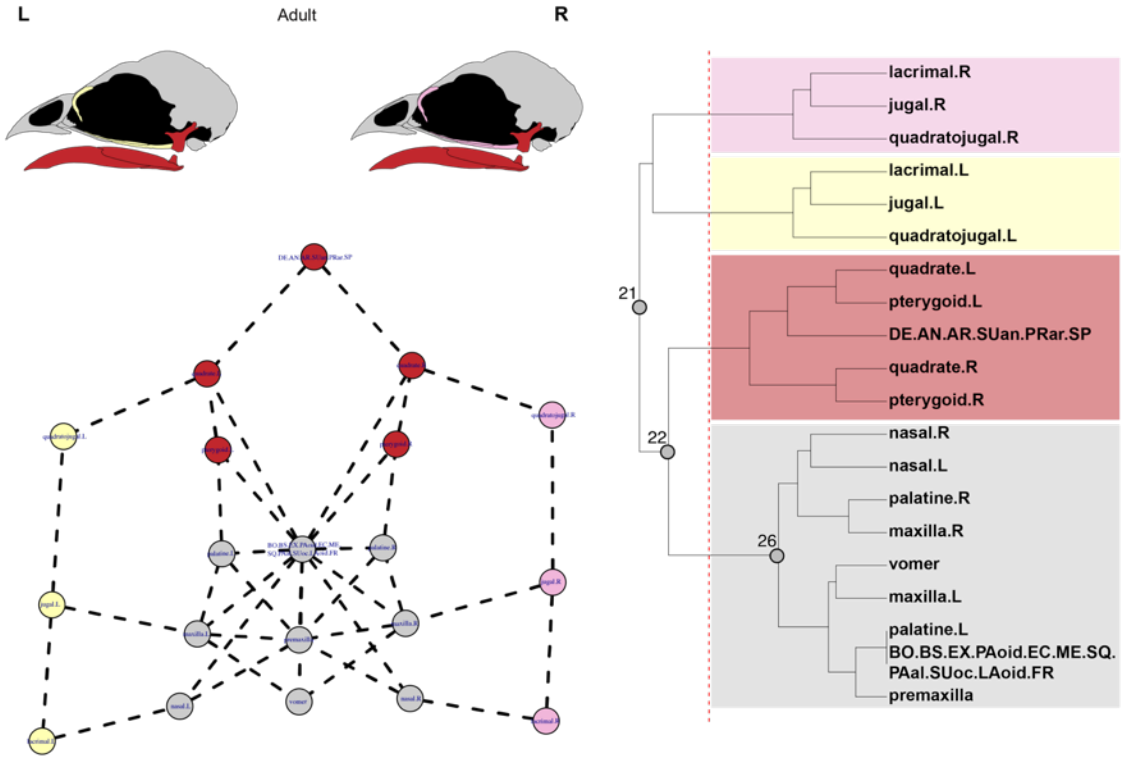

L
Adult
R
21
22
26
lacrimal.R
jugal.R
quadratojugal.R
lacrimal.L
jugal.L
quadratojugal.L
quadrate.L
pterygoid.L
DE.AN.AR.SUan.PRar.SP
quadrate.R
pterygoid.R
nasal.R
nasal.L
palatine.R
maxilla.R
vomer
maxilla.L
palatine.L
BO.BS.EX.PAoid.EC.ME.SQ.
PAal.SUoc.LAoid.FR
premaxilla

Dromaius novaehollandiae

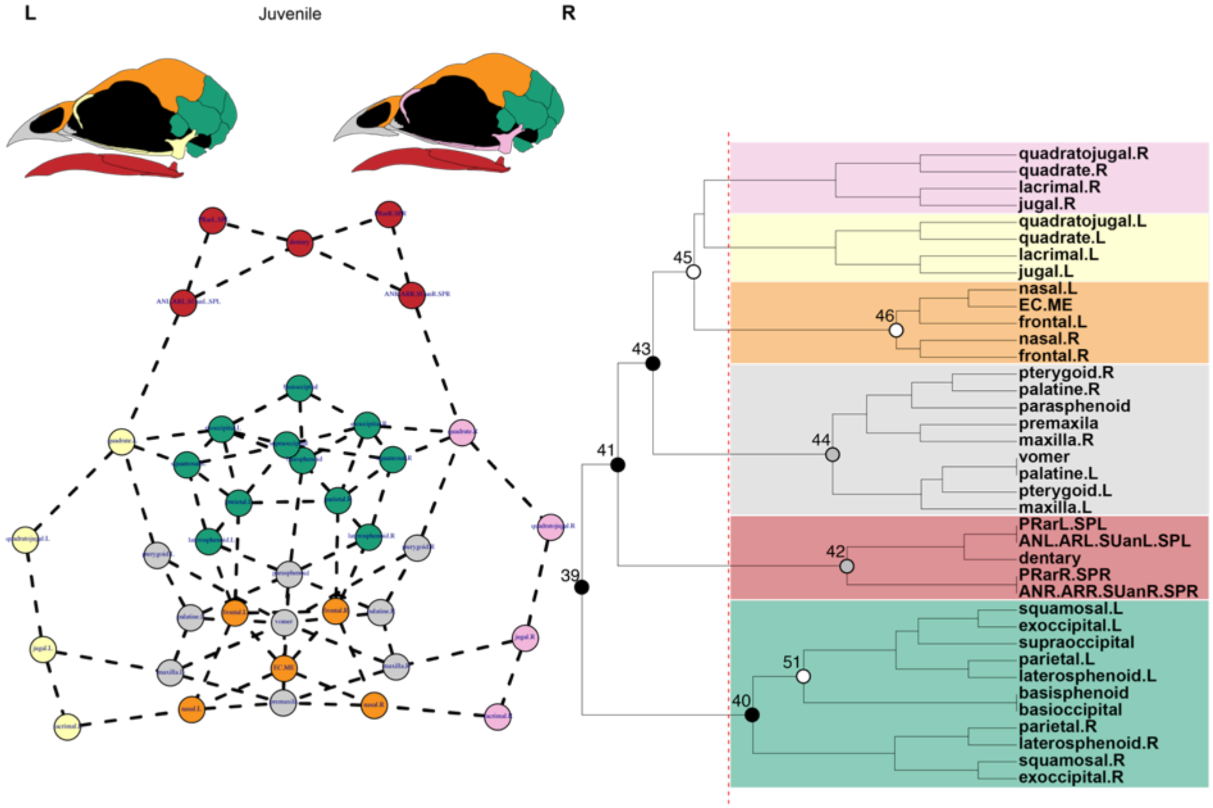

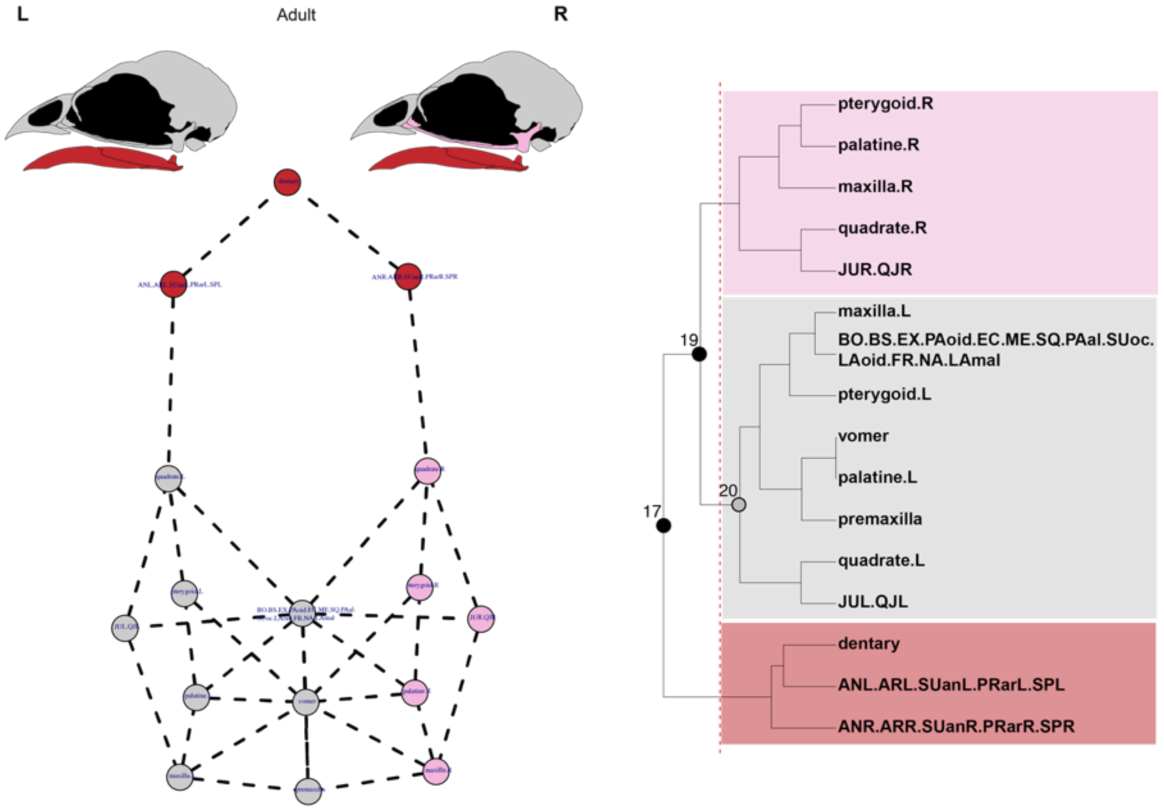

Pelecanus occidentalis

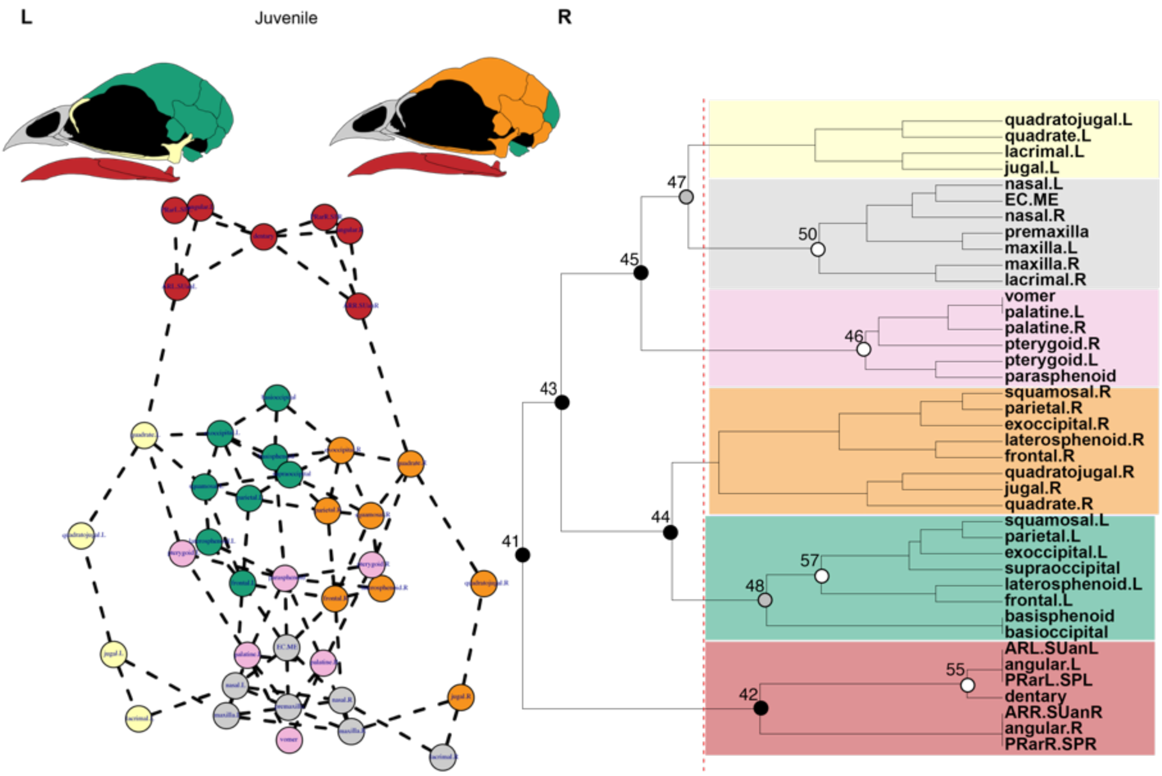

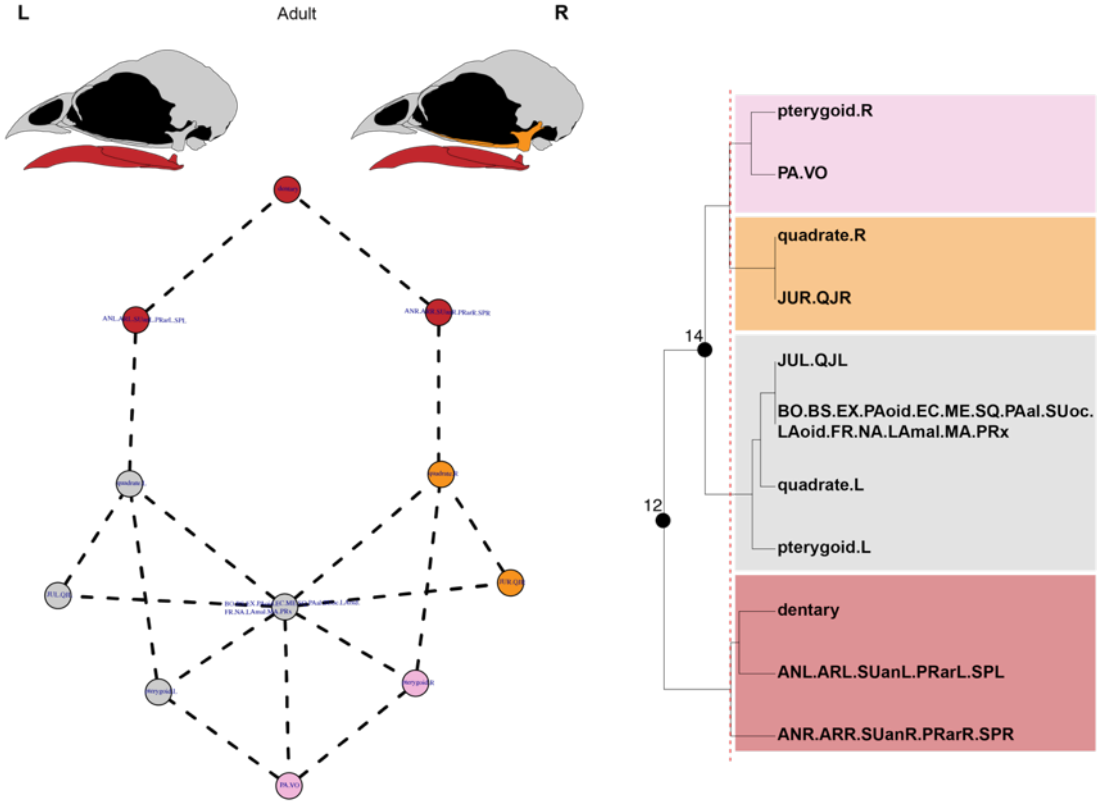

Theristicus caudatus

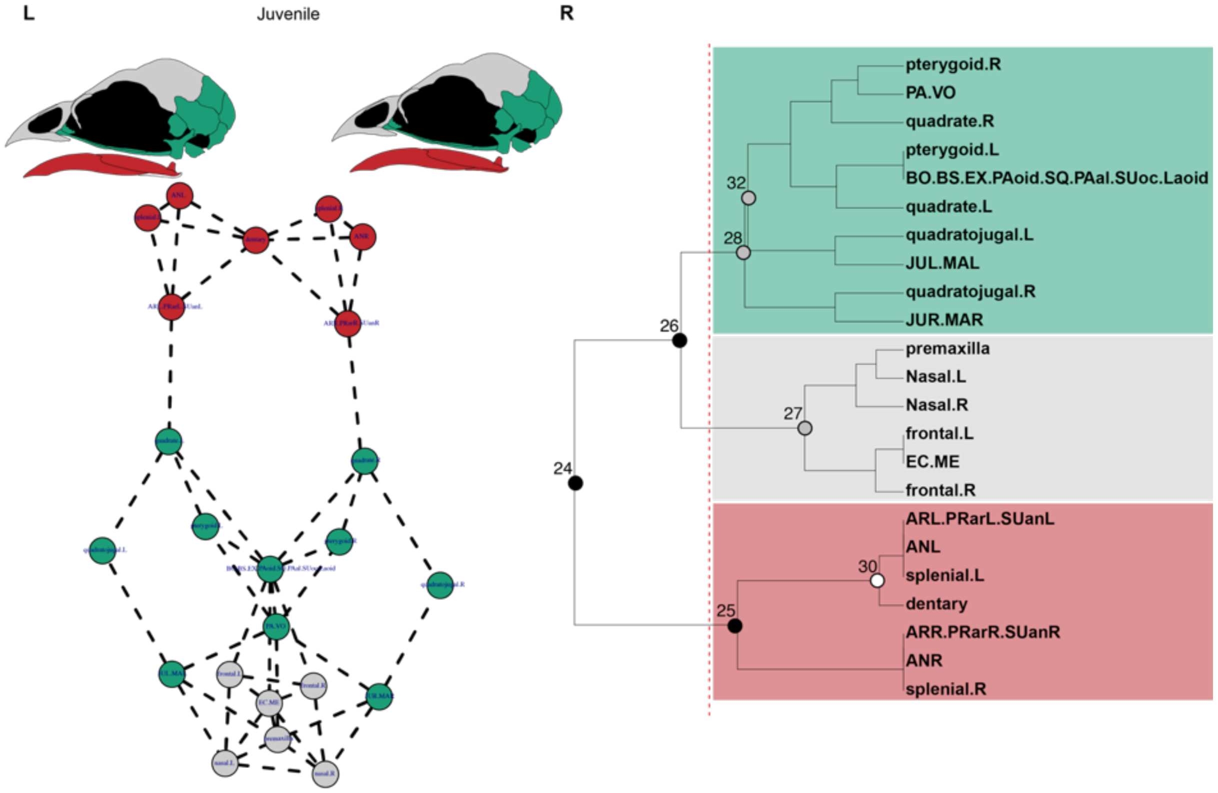

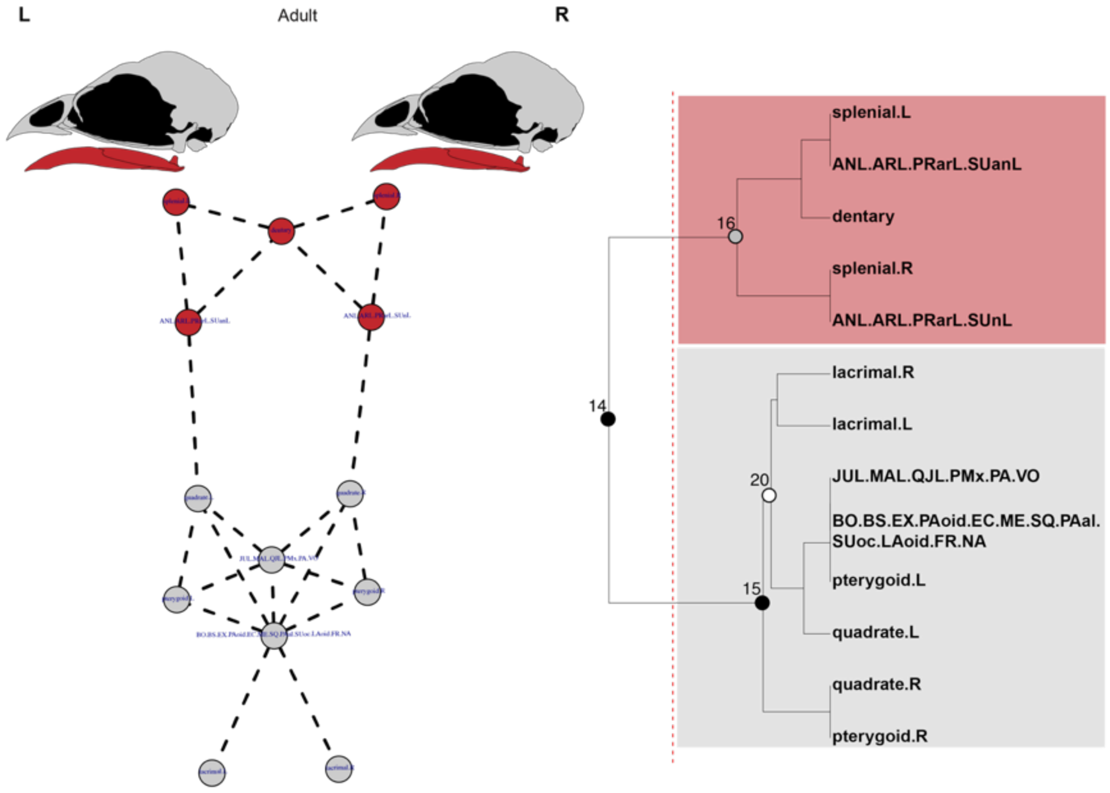

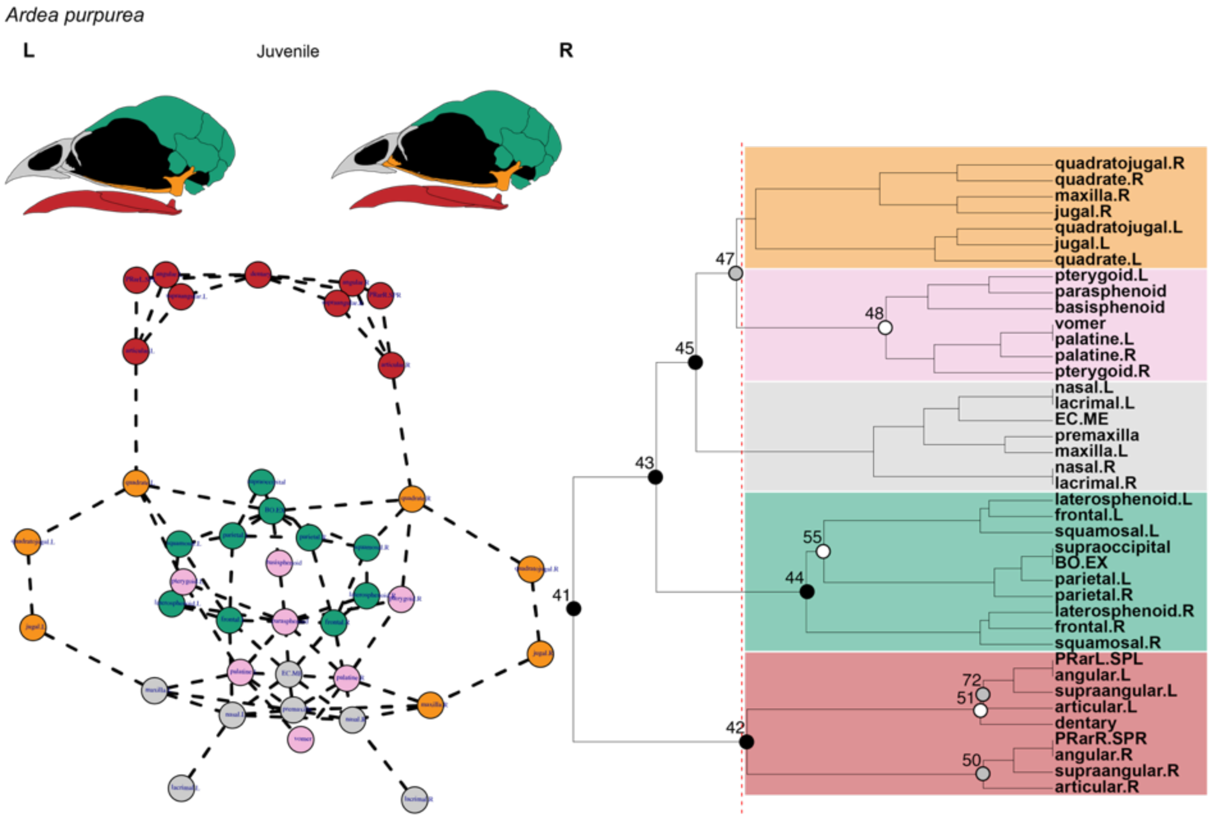

Ardea purpurea
L
Juvenile
R
47
48
45
43
55
44
41
72
51
42
50
quadratojugal.R
quadrate.R
maxilla.R
jugal.R
quadratojugal.L
jugal.L
quadrate.L
pterygoid.L
parasphenoid
basisphenoid
vomer
palatine.L
palatine.R
pterygoid.R
nasal.L
lacrimal.L
EC.ME
premaxilla
maxilla.L
nasal.R
lacrimal.R
laterosphenoid.L
frontal.L
squamosal.L
supraoccipital
BO.EX
parietal.L
parietal.R
laterosphenoid.R
frontal.R
squamosal.R
PRarL.SPL
angular.L
supraangular.L
articular.L
dentary
PRarR.SPR
angular.R
supraangular.R
articular.R

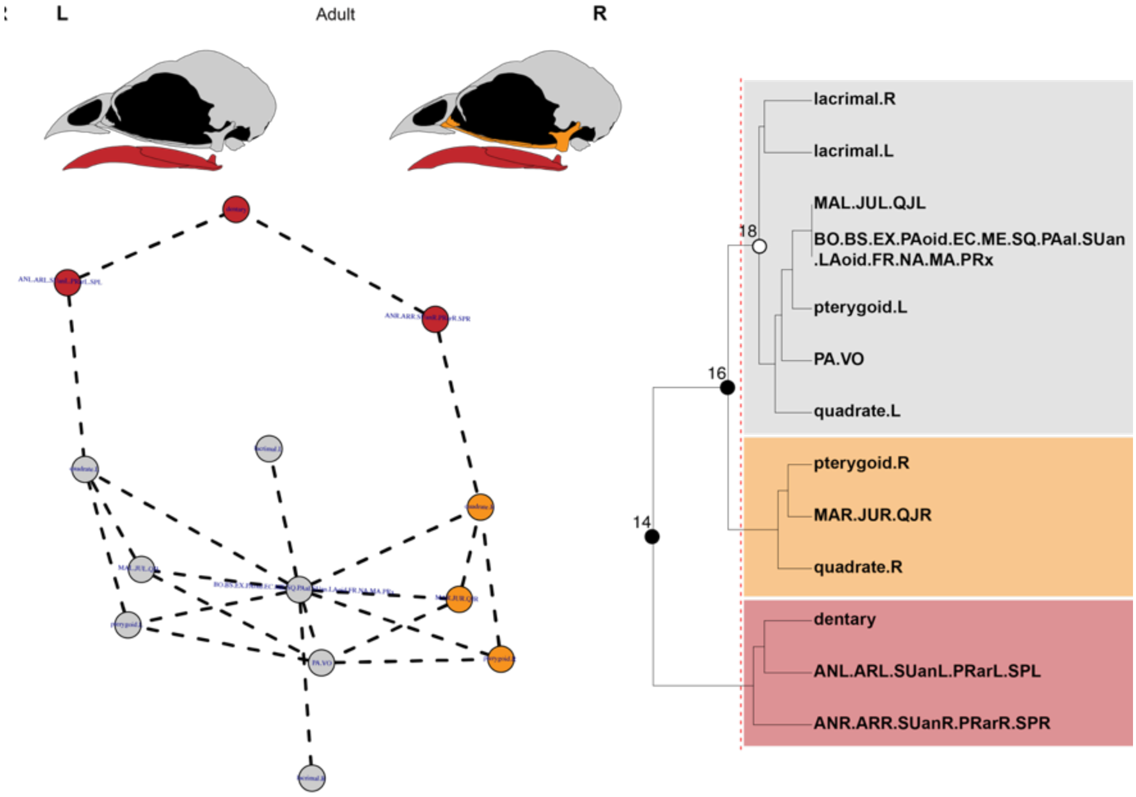

L
Adult
R
18
16
14
lacrimal.R
lacrimal.L
MAL.JUL.QJL
BO.BS.EX.PAoid.EC.ME.SQ.PAal.SUan
.LAoid.FR.NA.MA.PRx
pterygoid.L
PA.VO
quadrate.L
pterygoid.R
MAR.JUR.QJR
quadrate.R
dentary
ANL.ARL.SUanL.PRarL.SPL
ANR.ARR.SUanR.PRarR.SPR

Platalea leucorodia

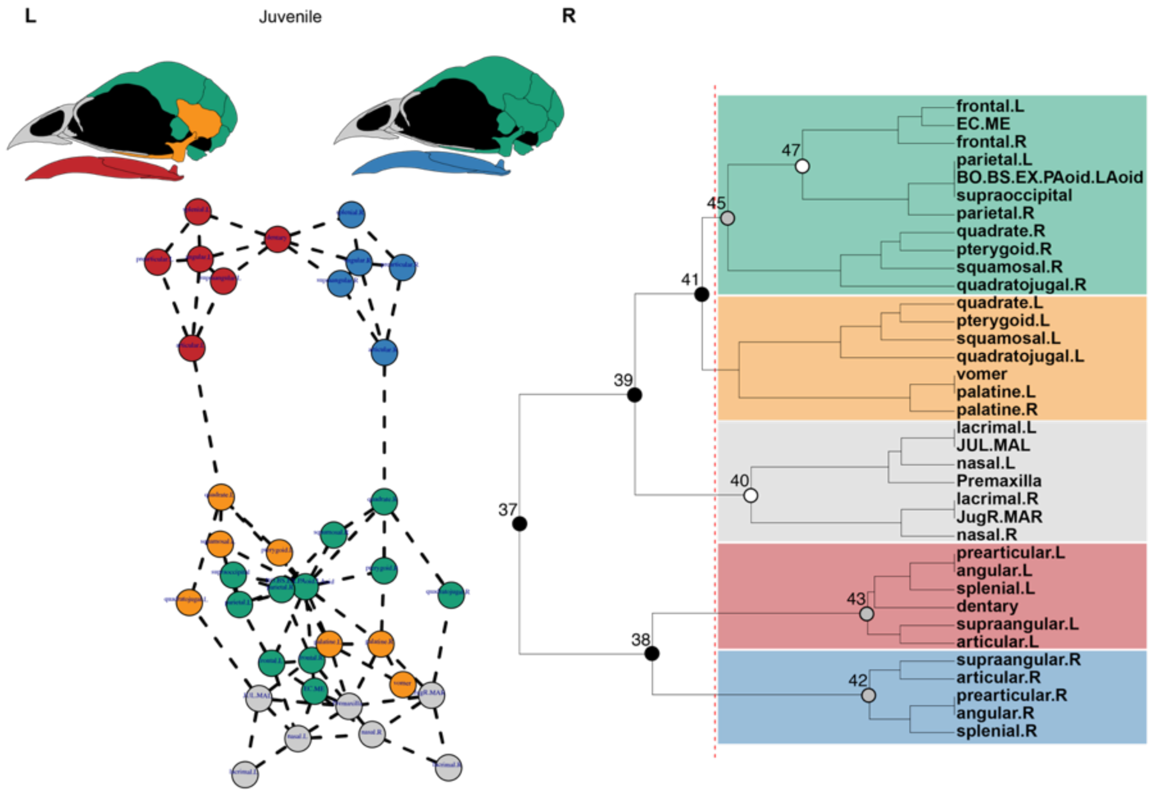

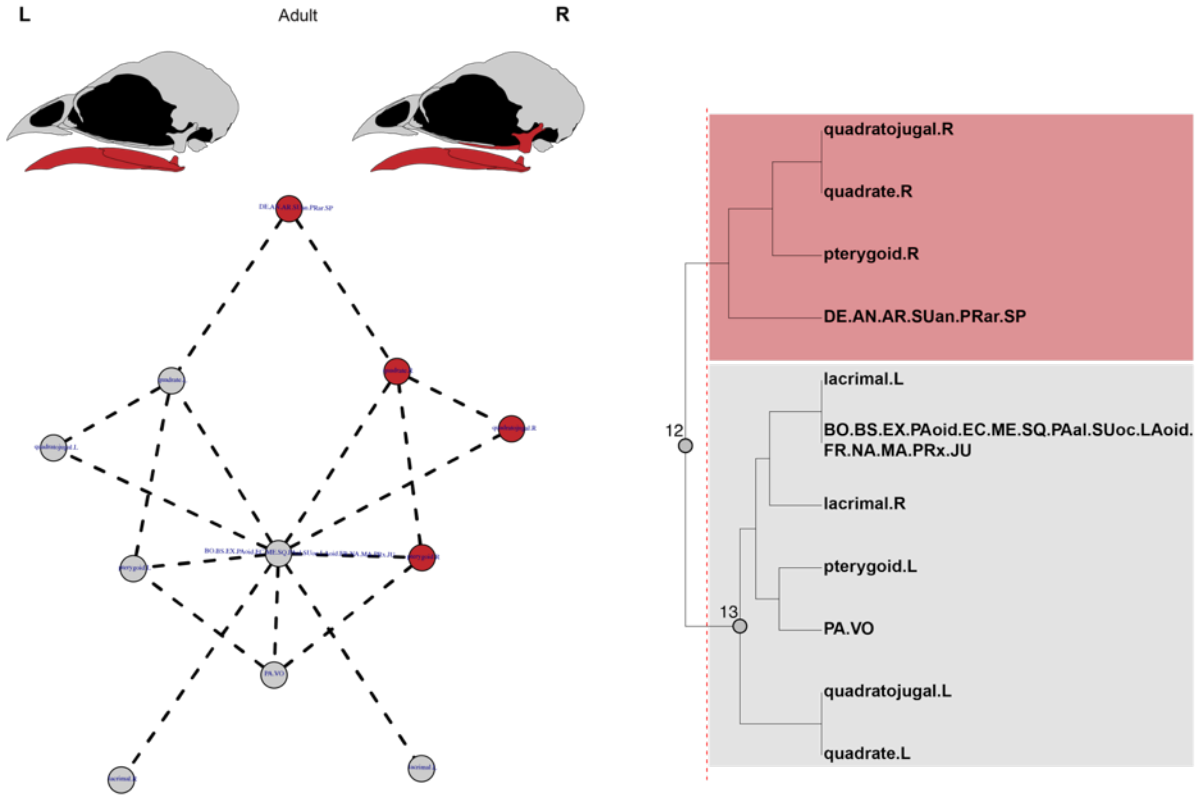

Platycercus elegans

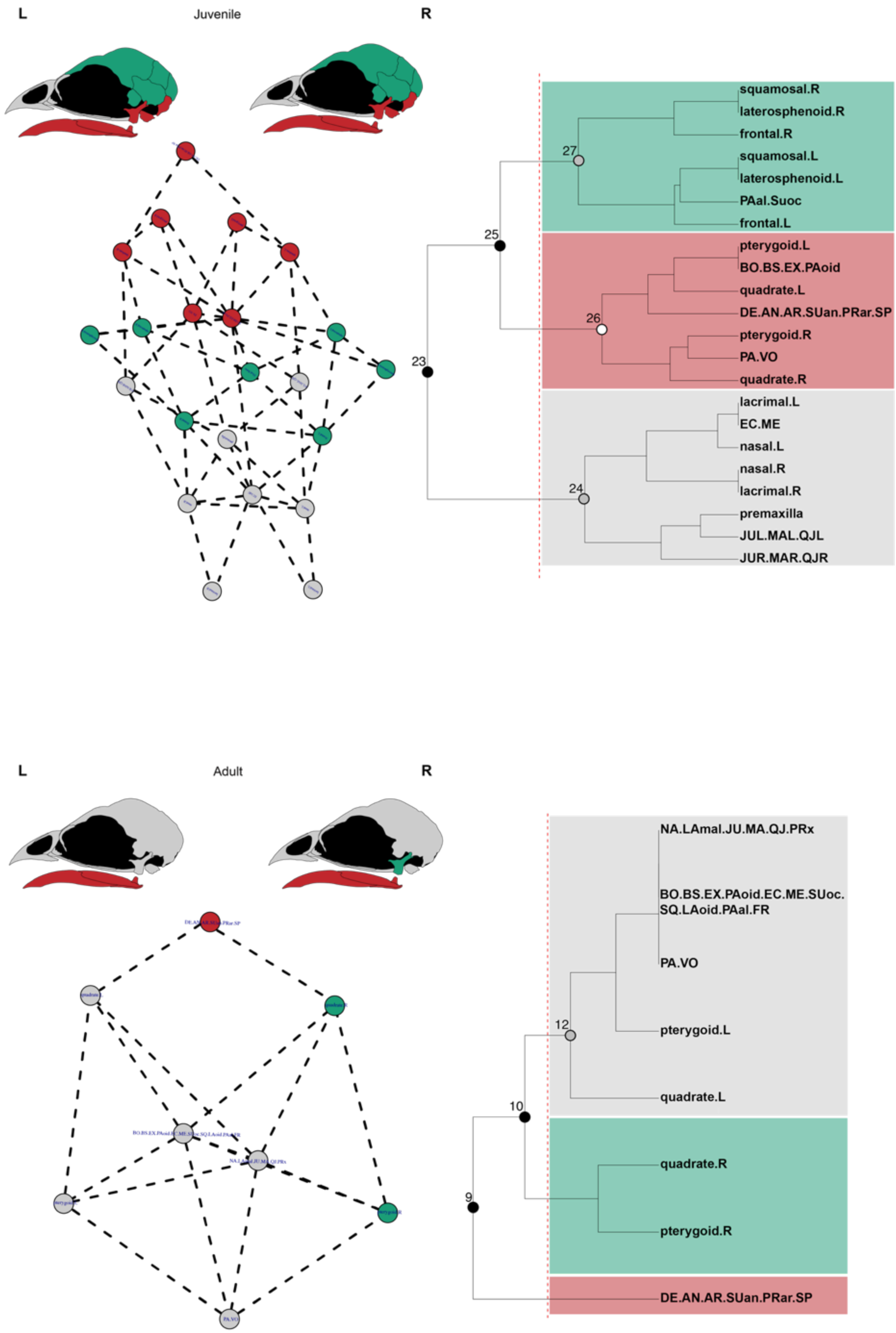

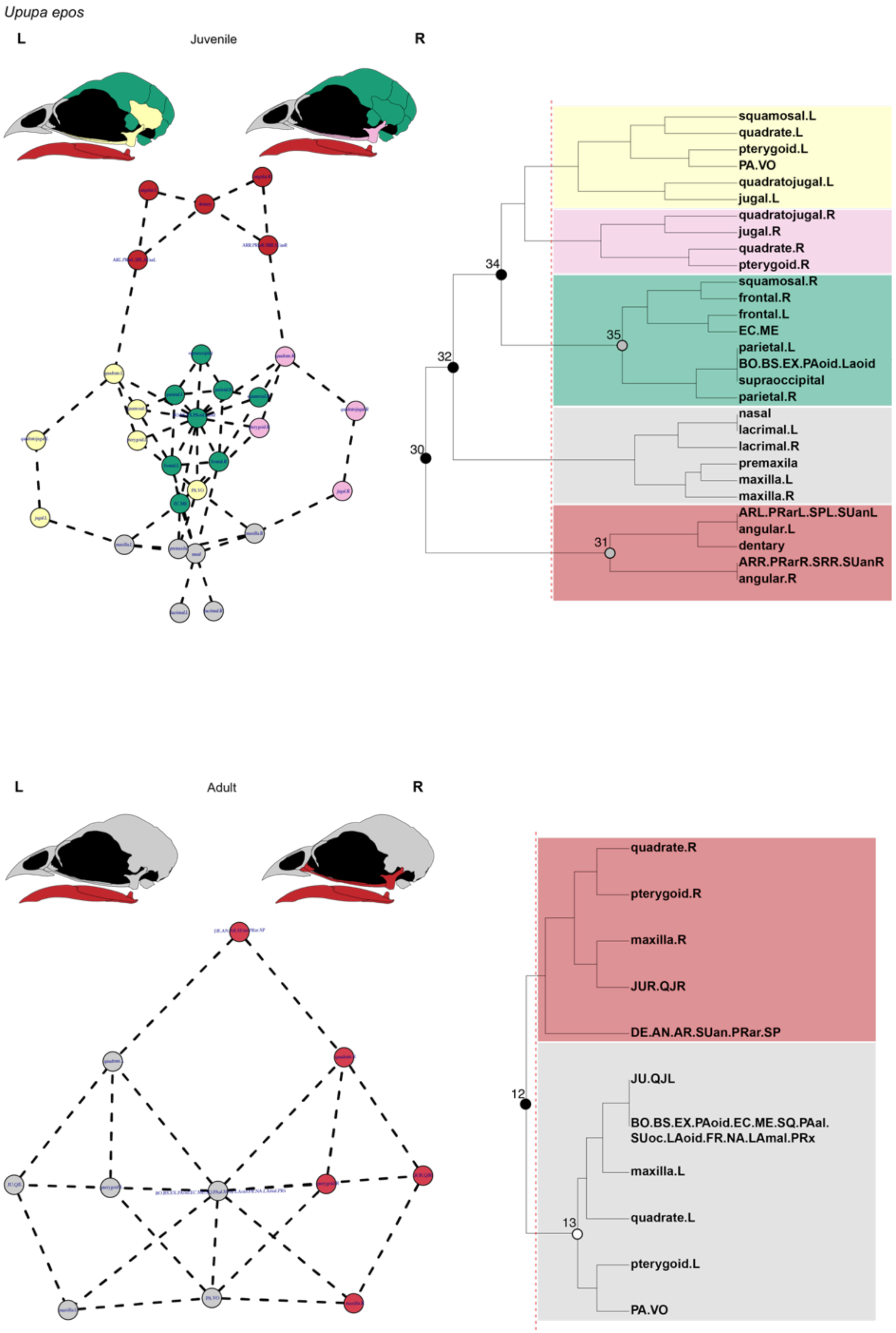
Upupa epos
L
Juvenile
R
squamosal.L
quadrate.L
pterygoid.L
PA.VO
quadratojugal.L
jugal.L
quadratojugal.R
jugal.R
quadrate.R
pterygoid.R
squamosal.R
frontal.R
frontal.L
EC.ME
parietal.L
BO.BS.EX.PAoid.Laoid
supraoccipital
parietal.R
nasal
lacrimal.L
lacrimal.R
premaxila
maxilla.L
maxilla.R
ARL.PRarL.SPL.SUanL
angular.L
dentary
ARR.PRarR.SRR.SUanR
angular.R
34
35
32
30
31
L
Adult
R
quadrate.R
pterygoid.R
maxilla.R
JUR.QJR
DE.AN.AR.SUan.PRar.SP
JU.QJL
BO.BS.EX.PAoid.EC.ME.SQ.PAal.
SUoc.LAoid.FR.NA.LAmal.PRx
maxilla.L
quadrate.L
pterygoid.L
PA.VO
12
13

Pygoscelis papua

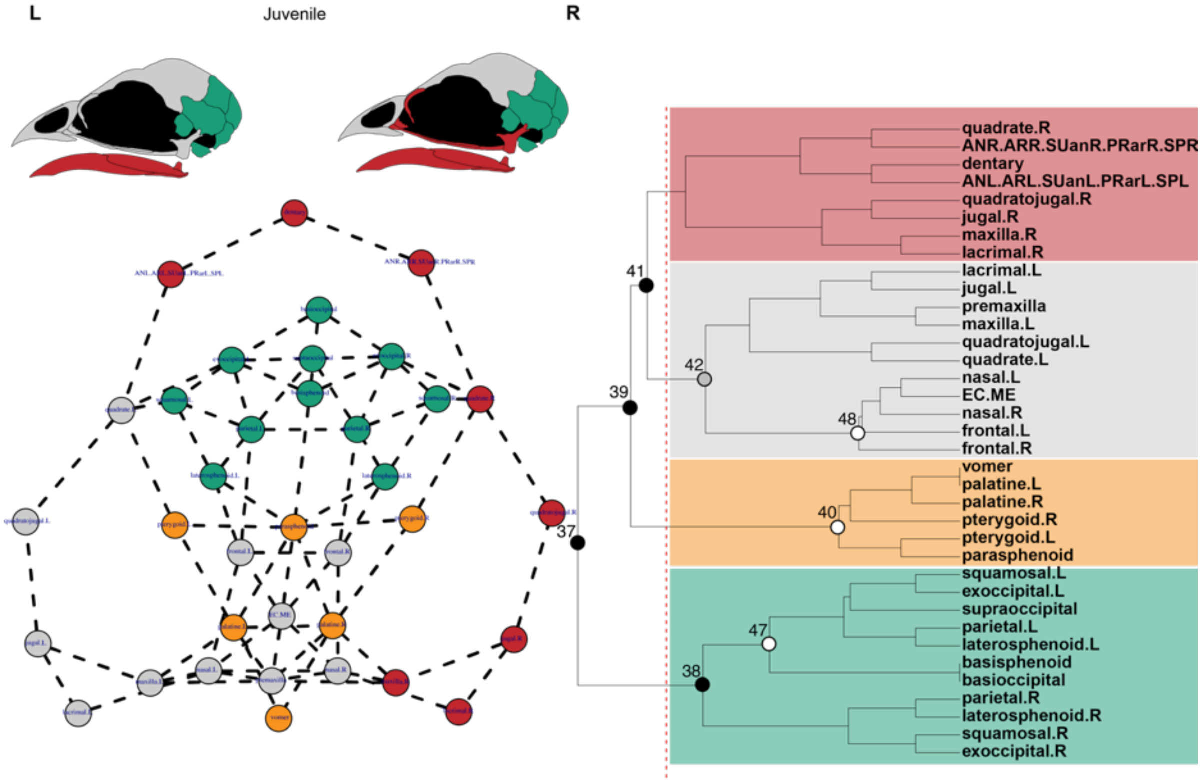

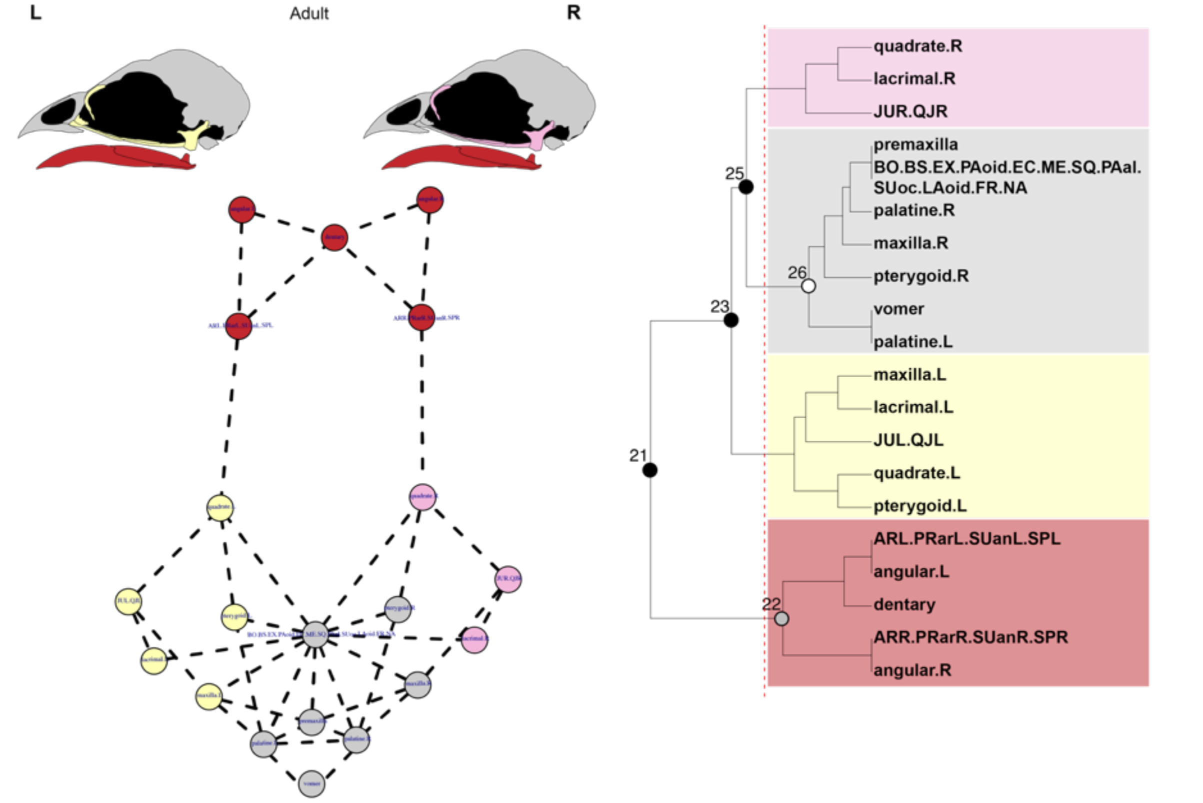

Spheniscus humboldti

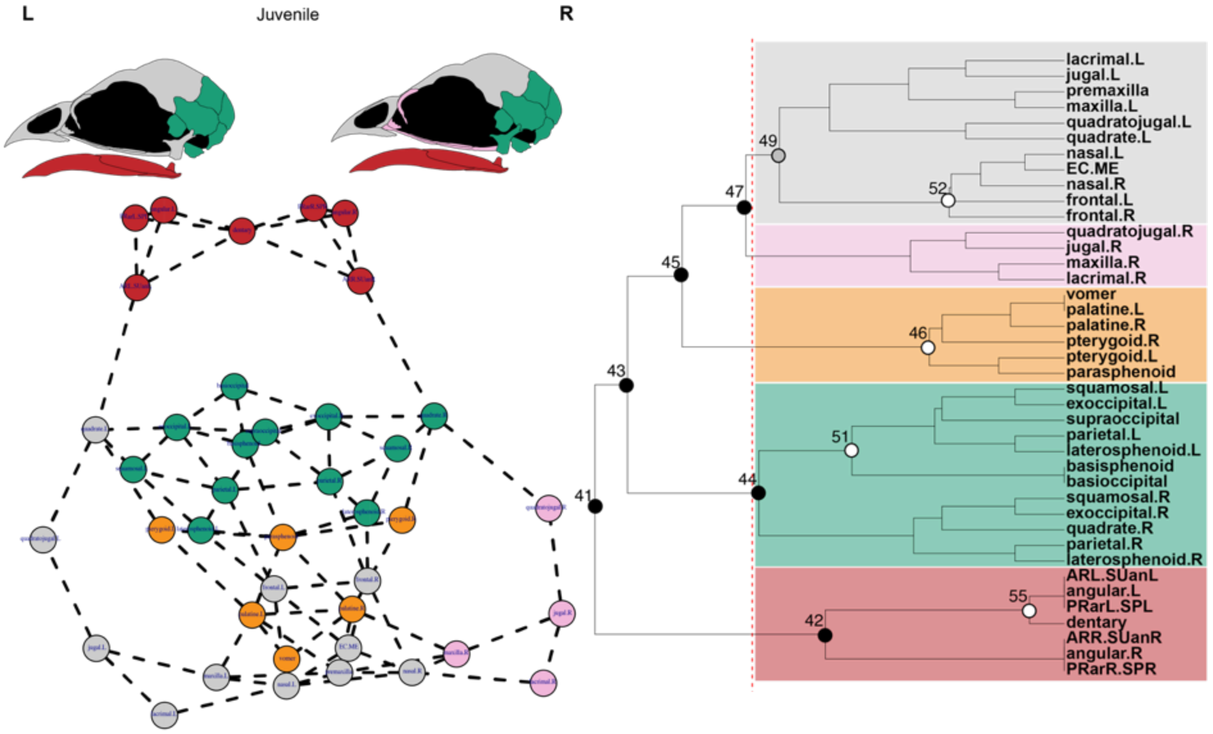

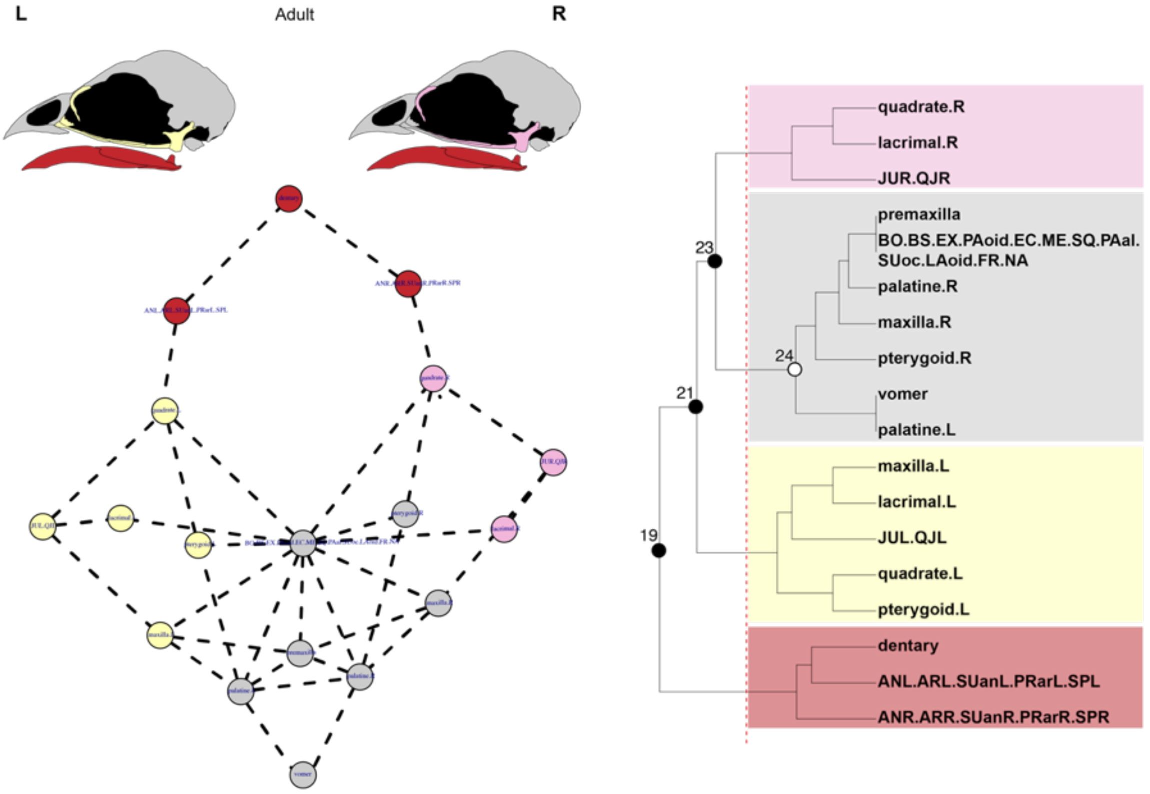

Spheniscus magellanicus

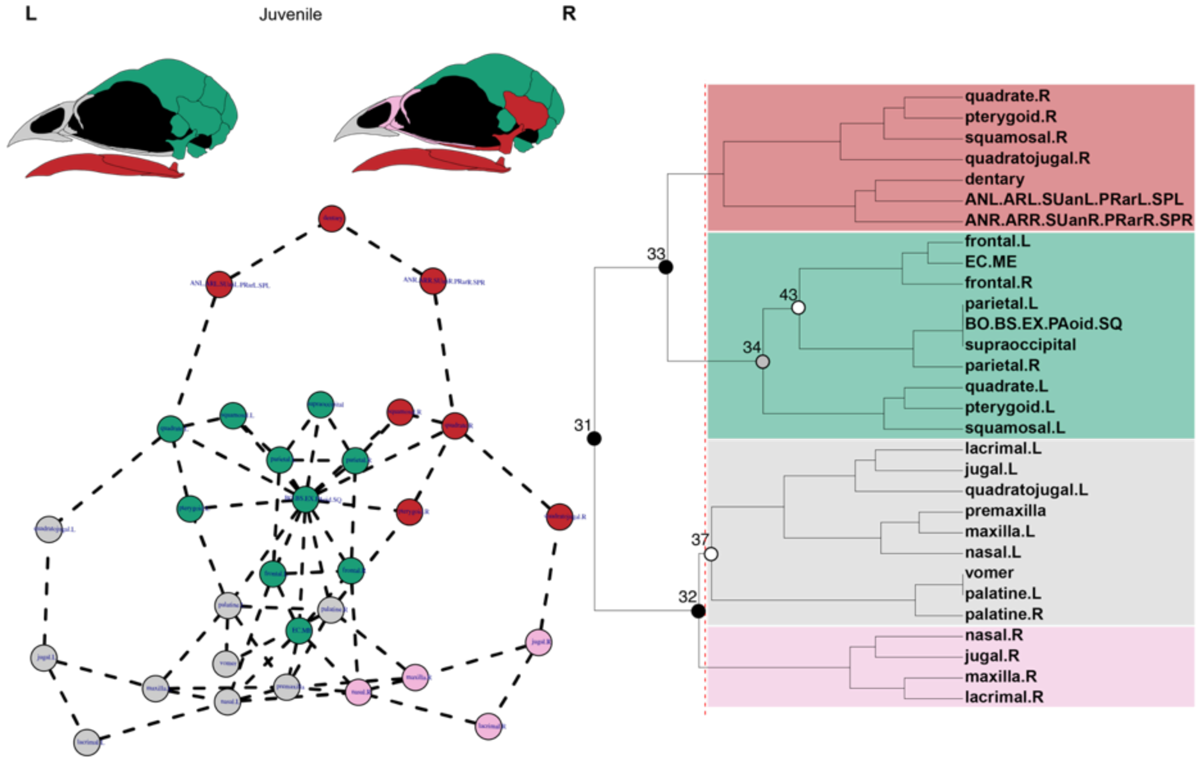

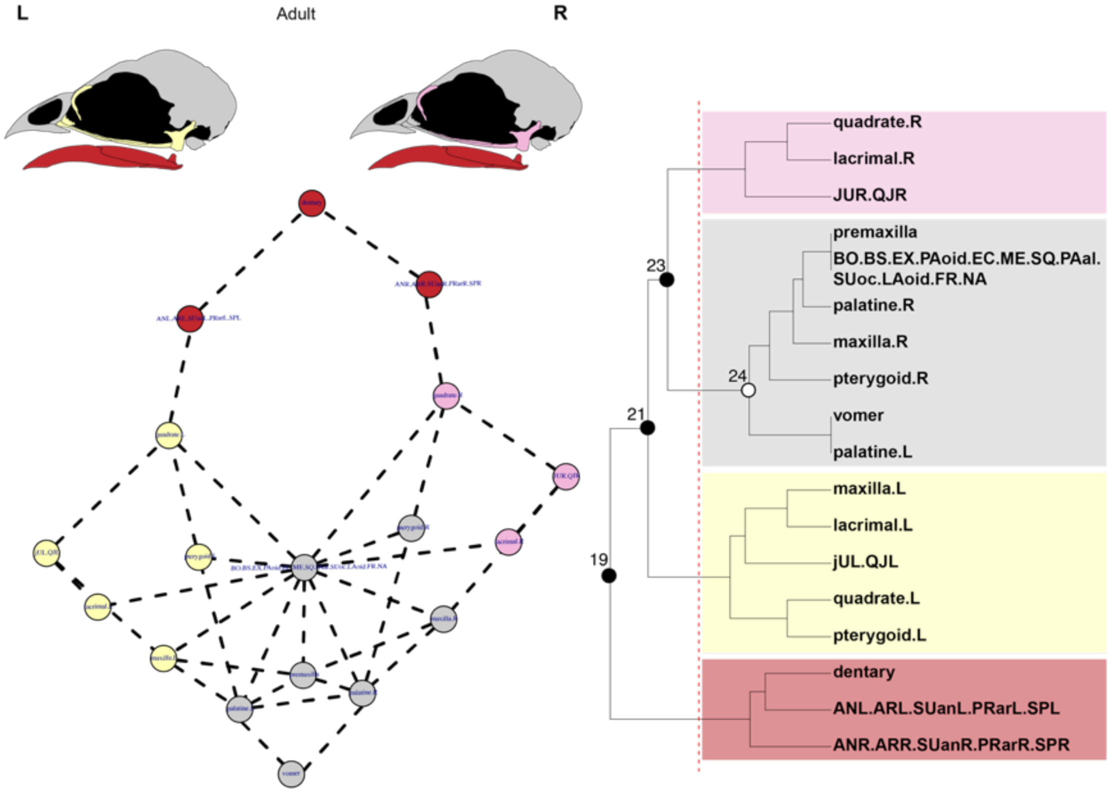

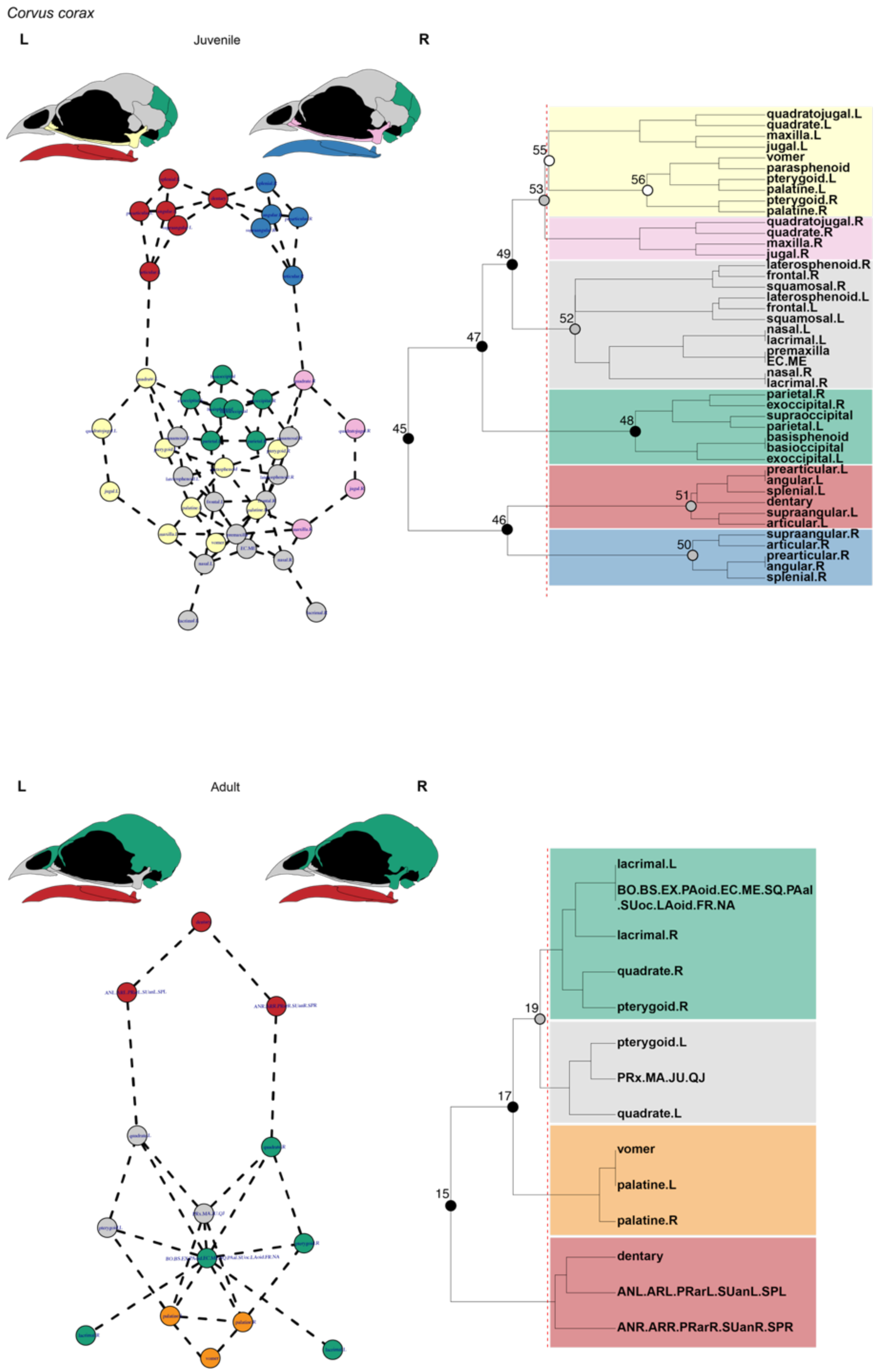
Corvus corax
L
Juvenile
R
55
53
56
49
52
47
45
48
51
46
50
quadratojugal.L
quadrate.L
maxilla.L
jugal.L
vomer
parasphenoid
pterygoid.L
palatine.L
pterygoid.R
palatine.R
quadratojugal.R
quadrate.R
maxilla.R
jugal.R
laterosphenoid.R
frontal.R
squamosal.R
laterosphenoid.L
frontal.L
squamosal.L
nasal.L
lacrimal.L
premaxilla
EC.ME
nasal.R
lacrimal.R
parietal.R
exoccipital.R
supraoccipital
parietal.L
basisphenoid
basioccipital
exoccipital.L
prearticular.L
angular.L
splenial.L
dentary
supraangular.L
articular.L
supraangular.R
articular.R
prearticular.R
angular.R
splenial.R
L
Adult
R
19
17
15
lacrimal.L
BO.BS.EX.PAoid.EC.ME.SQ.PAal
.SUoc.LAoid.FR.NA
lacrimal.R
quadrate.R
pterygoid.R
pterygoid.L
PRx.MA.JU.QJ
quadrate.L
vomer
palatine.L
palatine.R
dentary
ANL.ARL.PRarL.SUanL.SPL
ANR.ARR.PRarR.SUanR.SPR

Corvus coronne

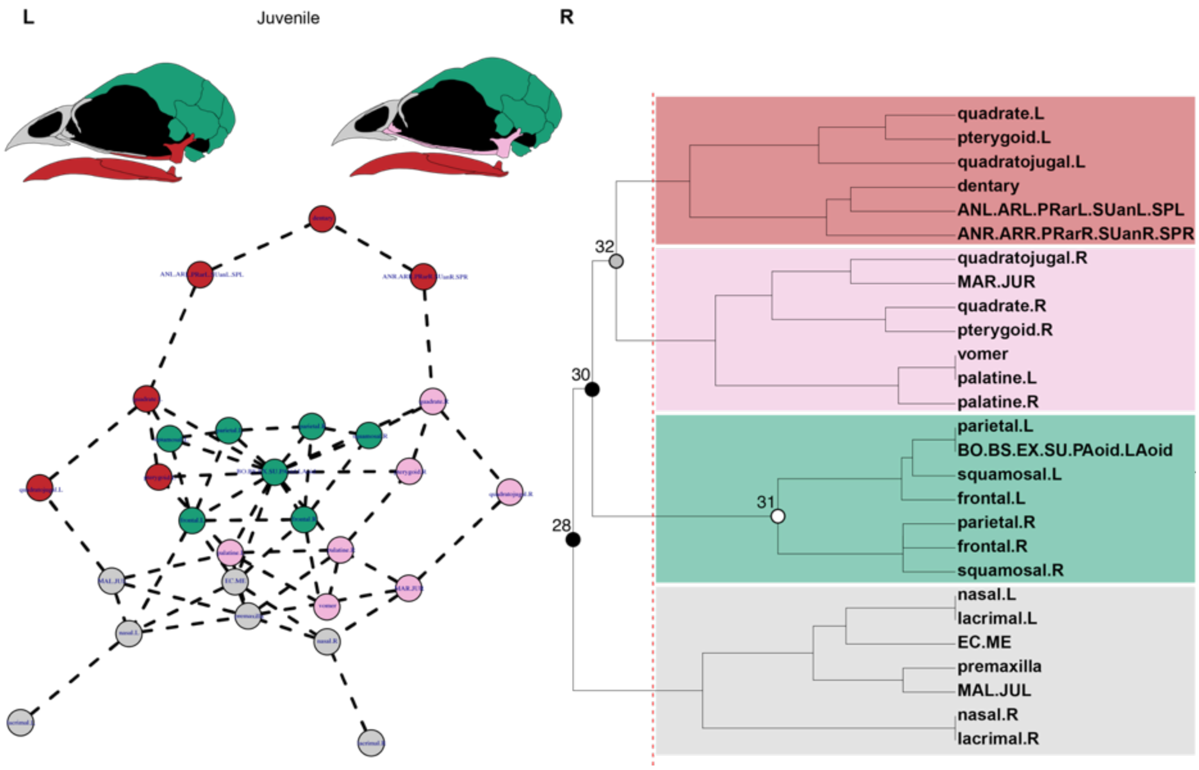

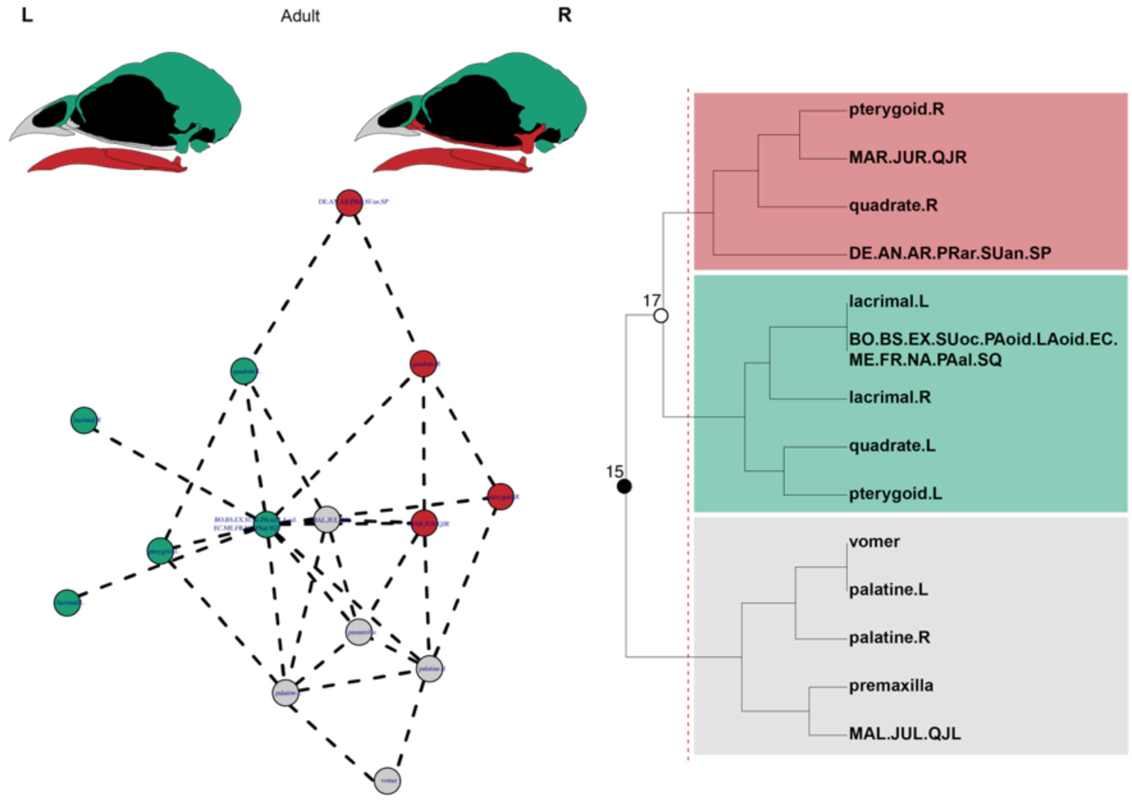

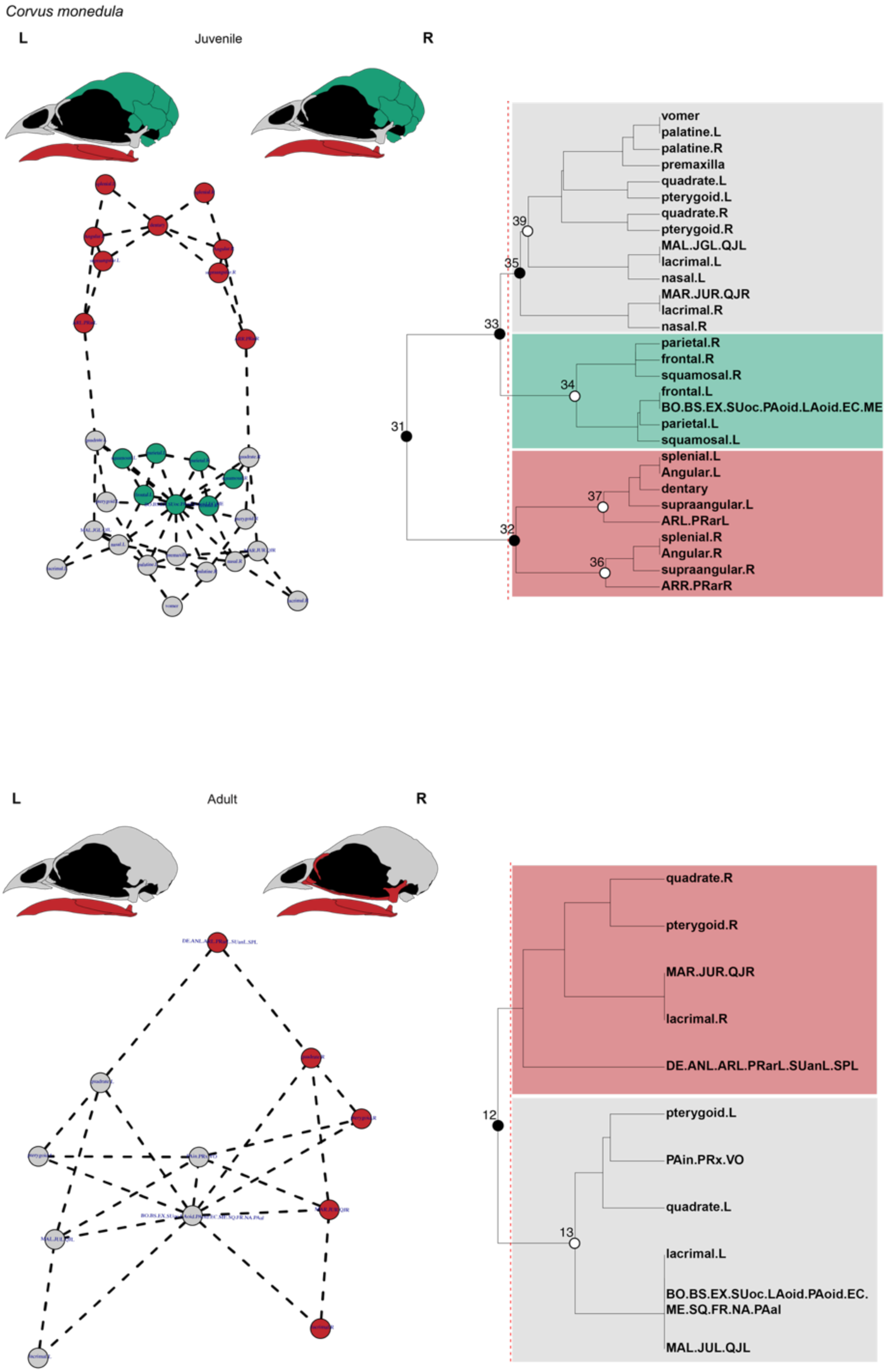
Corvus monedula
L
Juvenile
R
vomer
palatine.L
palatine.R
premaxilla
quadrate.L
pterygoid.L
quadrate.R
pterygoid.R
MAL.JGL.QJL
lacrimal.L
nasal.L
MAR.JUR.QJR
lacrimal.R
nasal.R
parietal.R
frontal.R
squamosal.R
frontal.L
BO.BS.EX.SUoc.PAoid.LAoid.EC.ME
parietal.L
squamosal.L
splenial.L
Angular.L
dentary
supraangular.L
ARL.PRarL
splenial.R
Angular.R
supraangular.R
ARR.PRarR
31
32
33
34
35
36
37
39
L
Adult
R
quadrate.R
pterygoid.R
MAR.JUR.QJR
lacrimal.R
DE.ANL.ARL.PRarL.SUanL.SPL
pterygoid.L
PAin.PRx.VO
quadrate.L
lacrimal.L
BO.BS.EX.SUoc.LAoid.PAoid.EC.
ME.SQ.FR.NA.PAal
MAL.JUL.QJL
12
13

Pica pica

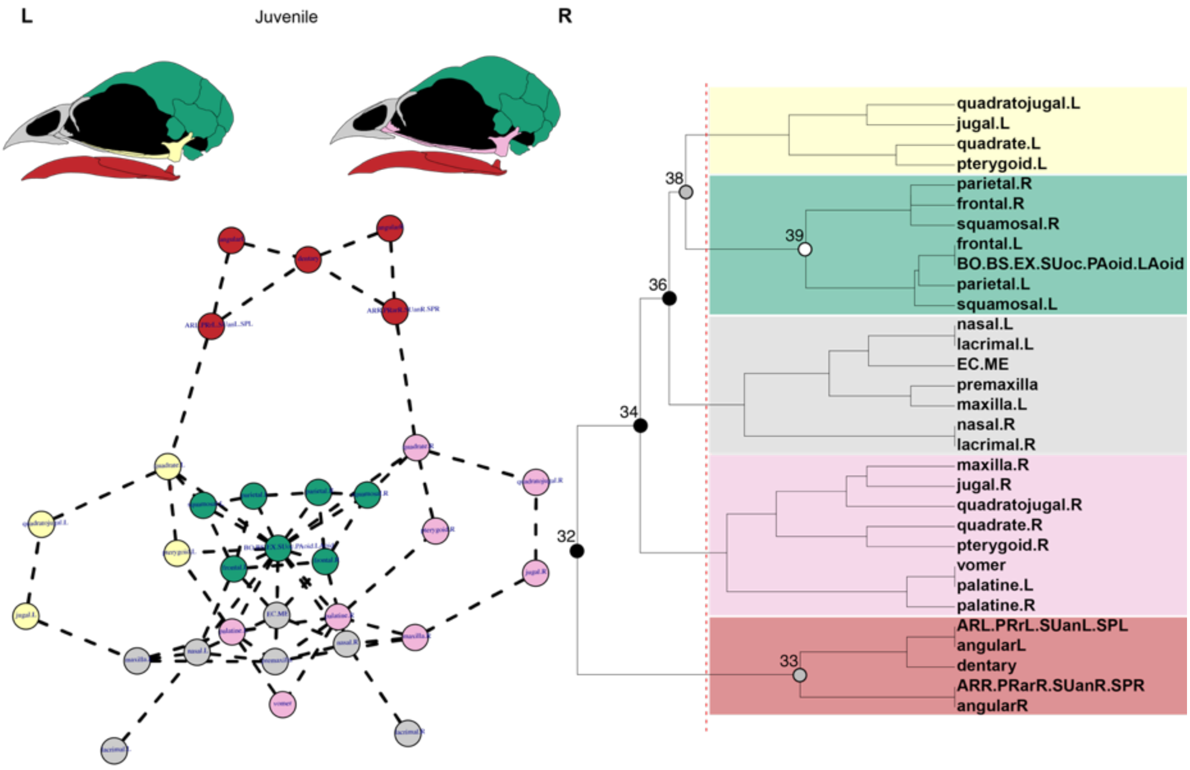
L
Juvenile
R
quadratojugal.L
jugal.L
quadrate.L
pterygoid.L
38
parietal.R
frontal.R
squamosal.R
39
frontal.L
BO.BS.EX.SUoc.PAoid.LAoid
parietal.L
squamosal.L
36
nasal.L
lacrimal.L
EC.ME
premaxilla
maxilla.L
34
nasal.R
lacrimal.R
maxilla.R
jugal.R
quadratojugal.R
quadrate.R
pterygoid.R
32
vomer
palatine.L
palatine.R
ARL.PRrL.SUanL.SPL
angularL
33
dentary
ARR.PRarR.SUanR.SPR
angularR

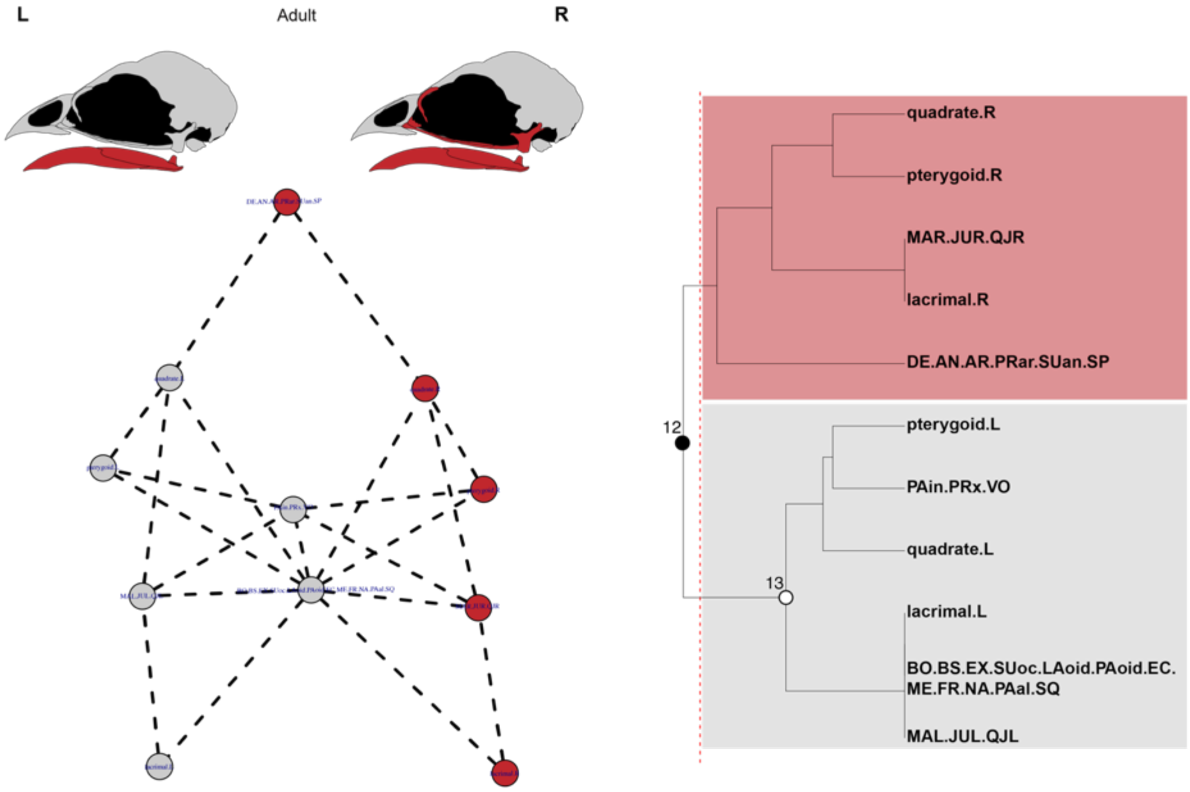
L
Adult
R
quadrate.R
pterygoid.R
MAR.JUR.QJR
lacrimal.R
DE.AN.AR.PRar.SUan.SP
12
pterygoid.L
PAin.PRx.VO
quadrate.L
13
lacrimal.L
BO.BS.EX.SUoc.LAoid.PAoid.EC.
ME.FR.NA.PAal.SQ
MAL.JUL.QJL

Phoenicopterus ruber

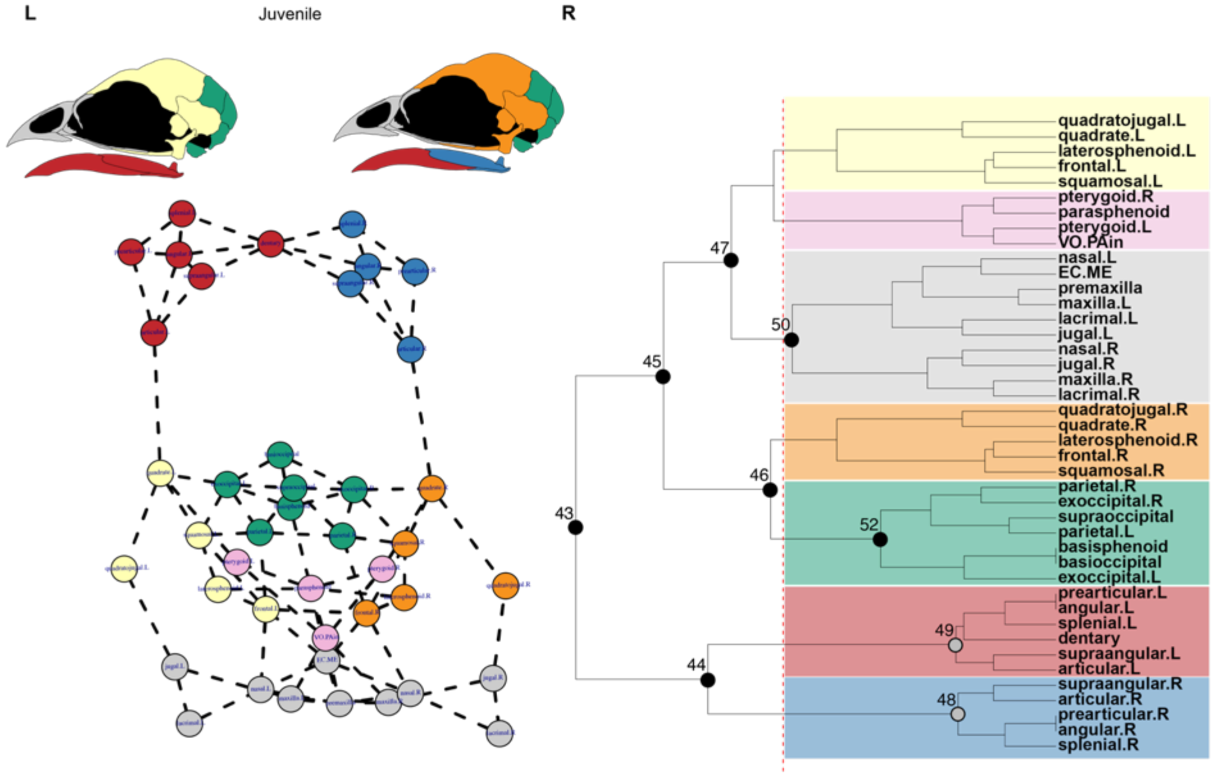

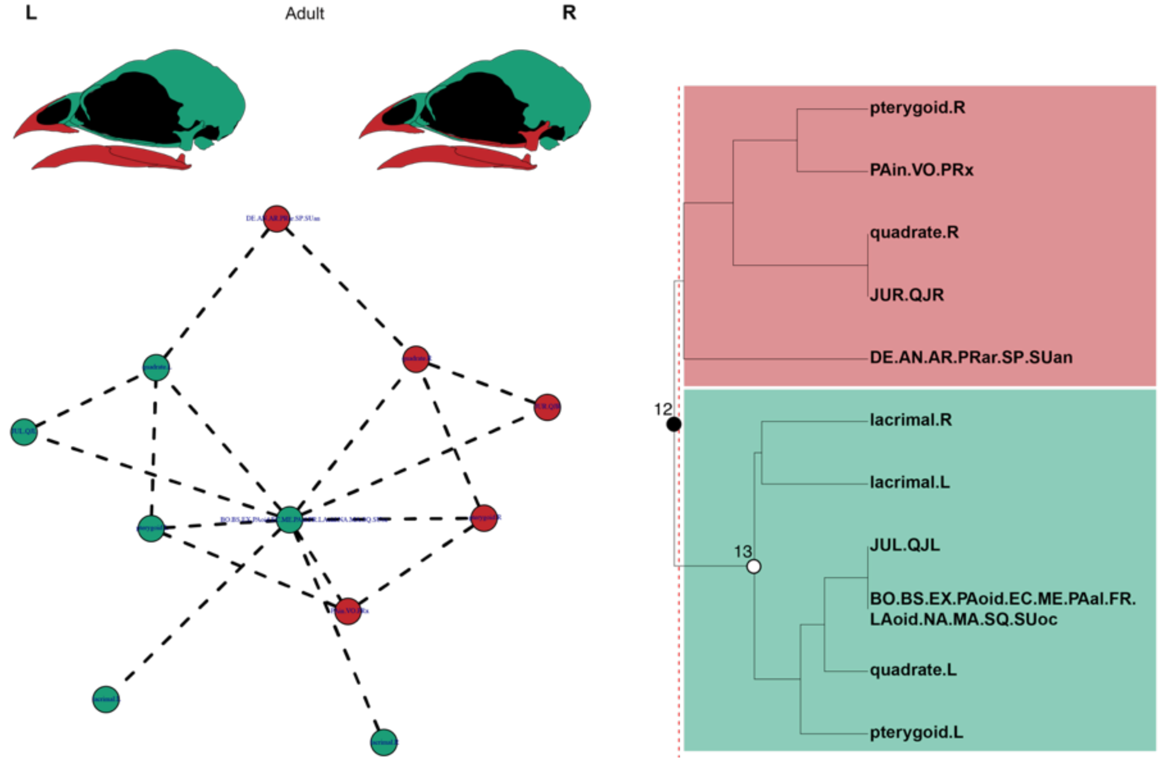

Podiceps cristatus

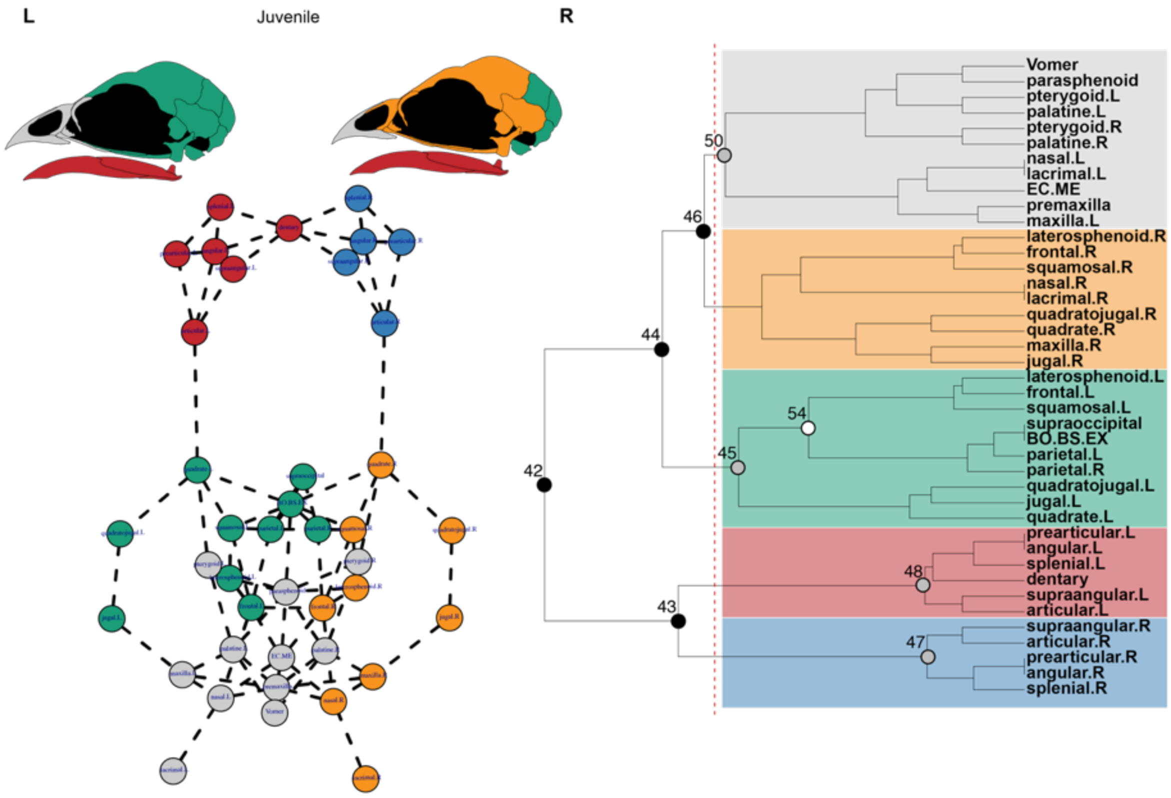

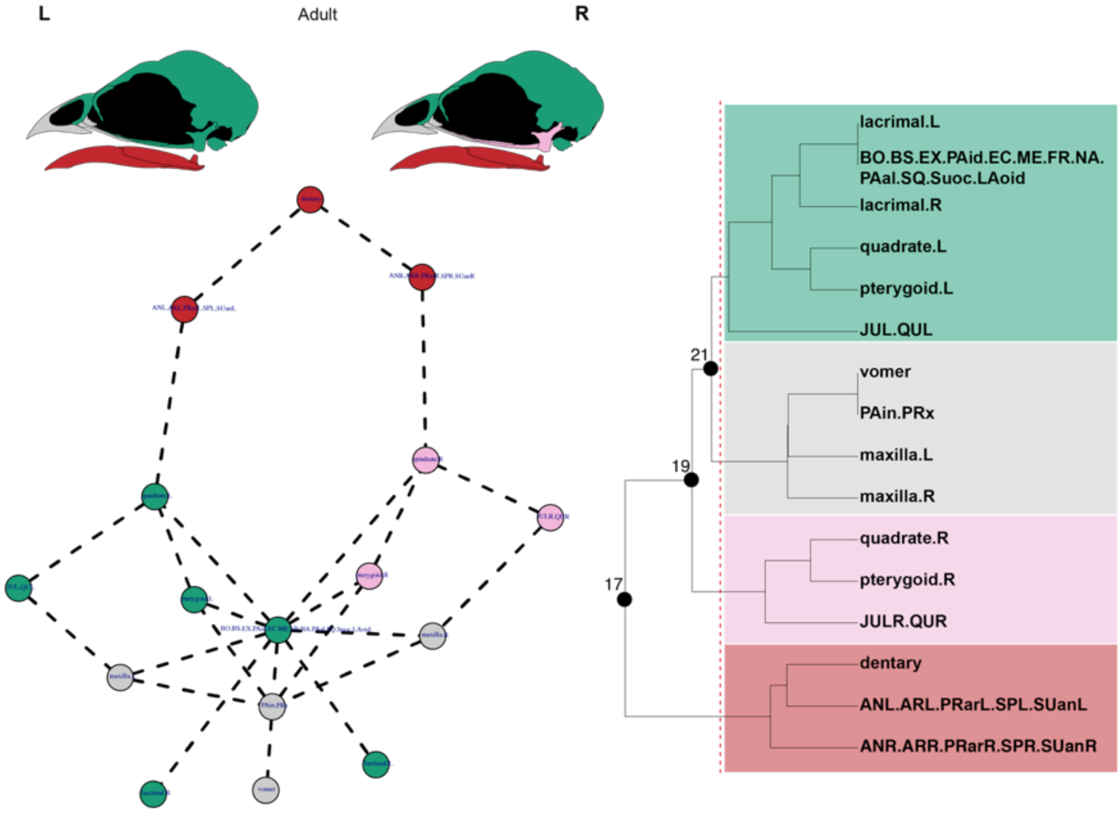

Apus apus

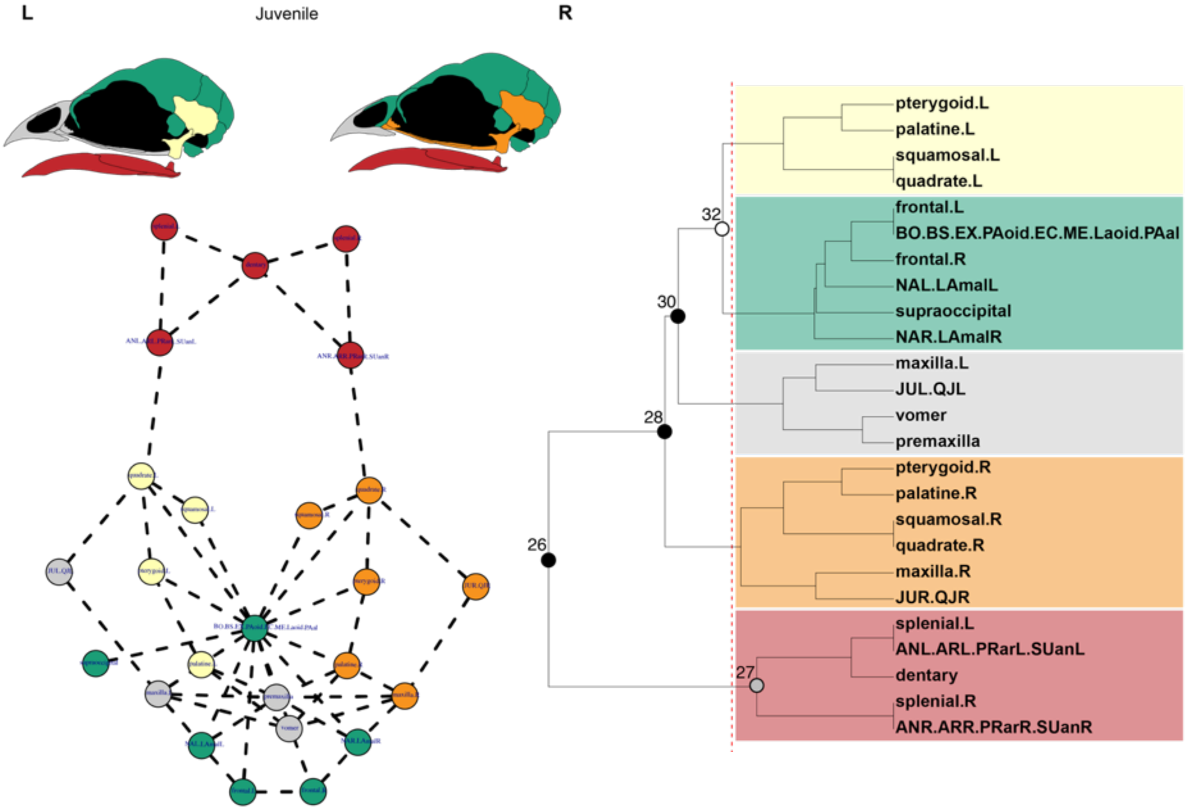
L
Juvenile
R
32
30
28
26
27
pterygoid.L
palatine.L
squamosal.L
quadrate.L
frontal.L
BO.BS.EX.PAoid.EC.ME.Laoid.PAal
frontal.R
NAL.LAmalL
supraoccipital
NAR.LAmalR
maxilla.L
JUL.QJL
vomer
premaxilla
pterygoid.R
palatine.R
squamosal.R
quadrate.R
maxilla.R
JUR.QJR
splenial.L
ANL.ARL.PRarL.SUanL
dentary
splenial.R
ANR.ARR.PRarR.SUanR

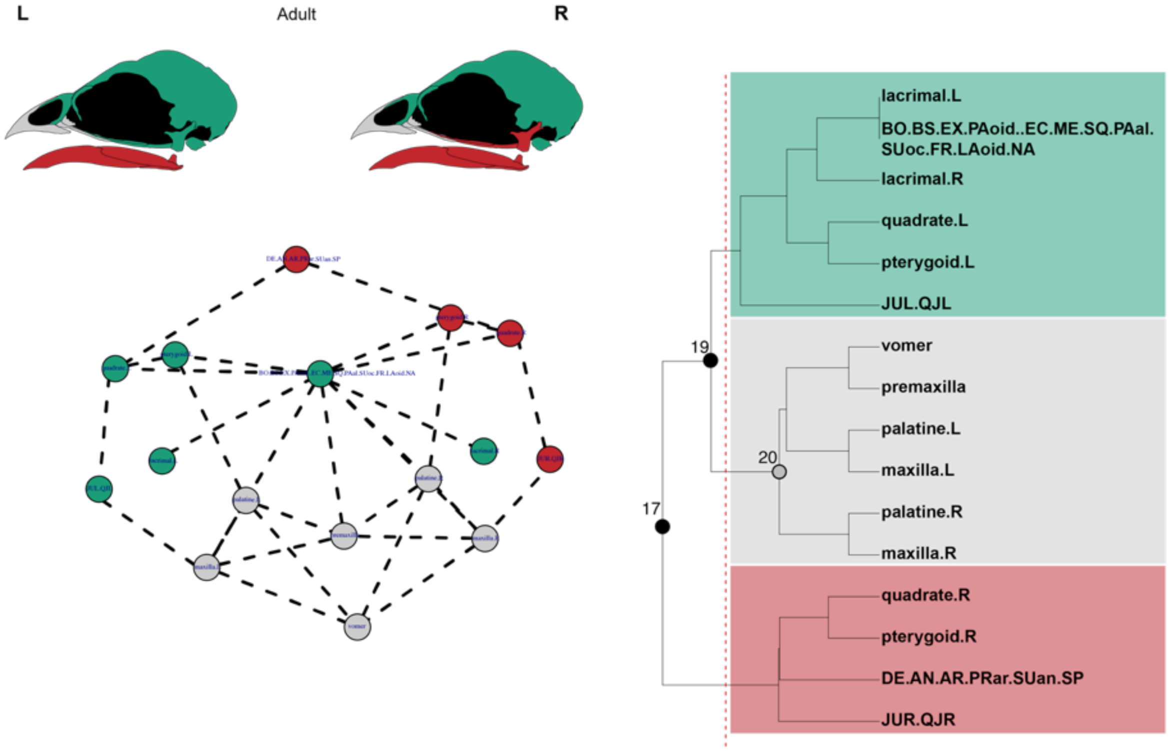
L
Adult
R
19
20
17
lacrimal.L
BO.BS.EX.PAoid..EC.ME.SQ.PAal.
SUoc.FR.LAoid.NA
lacrimal.R
quadrate.L
pterygoid.L
JUL.QJL
vomer
premaxilla
palatine.L
maxilla.L
palatine.R
maxilla.R
quadrate.R
pterygoid.R
DE.AN.AR.PRar.SUan.SP
JUR.QJR

Egretta garzetta

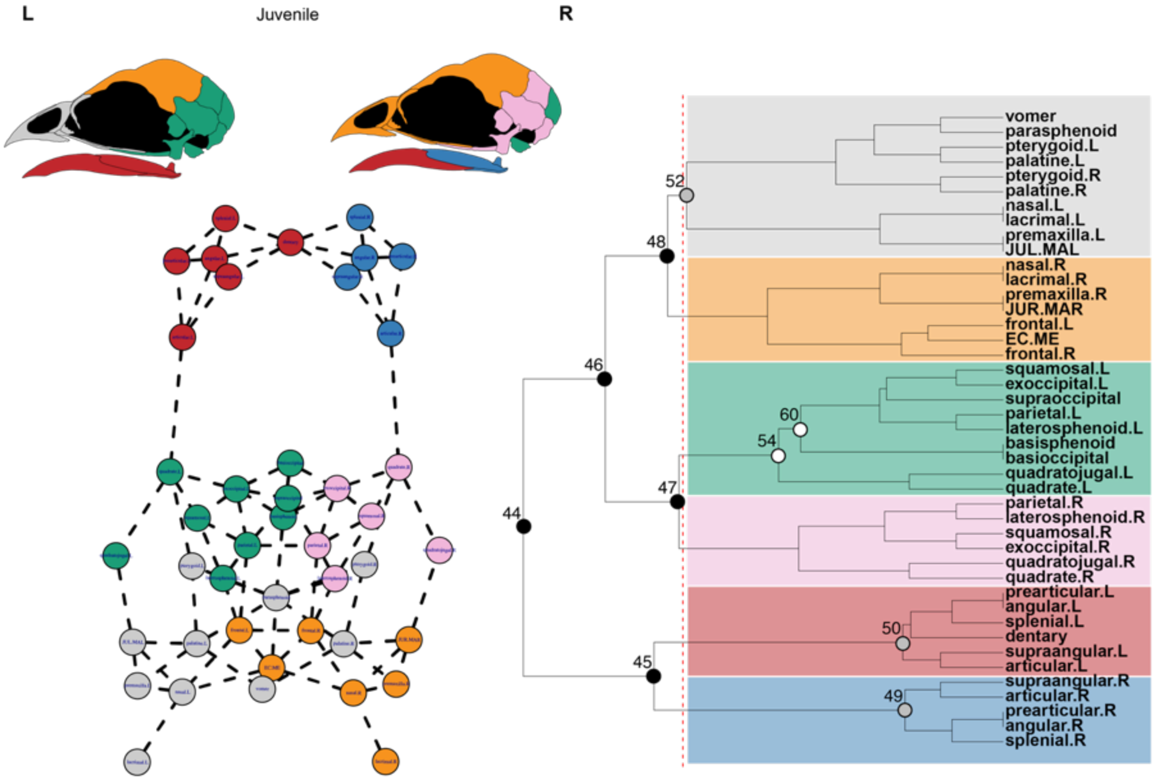

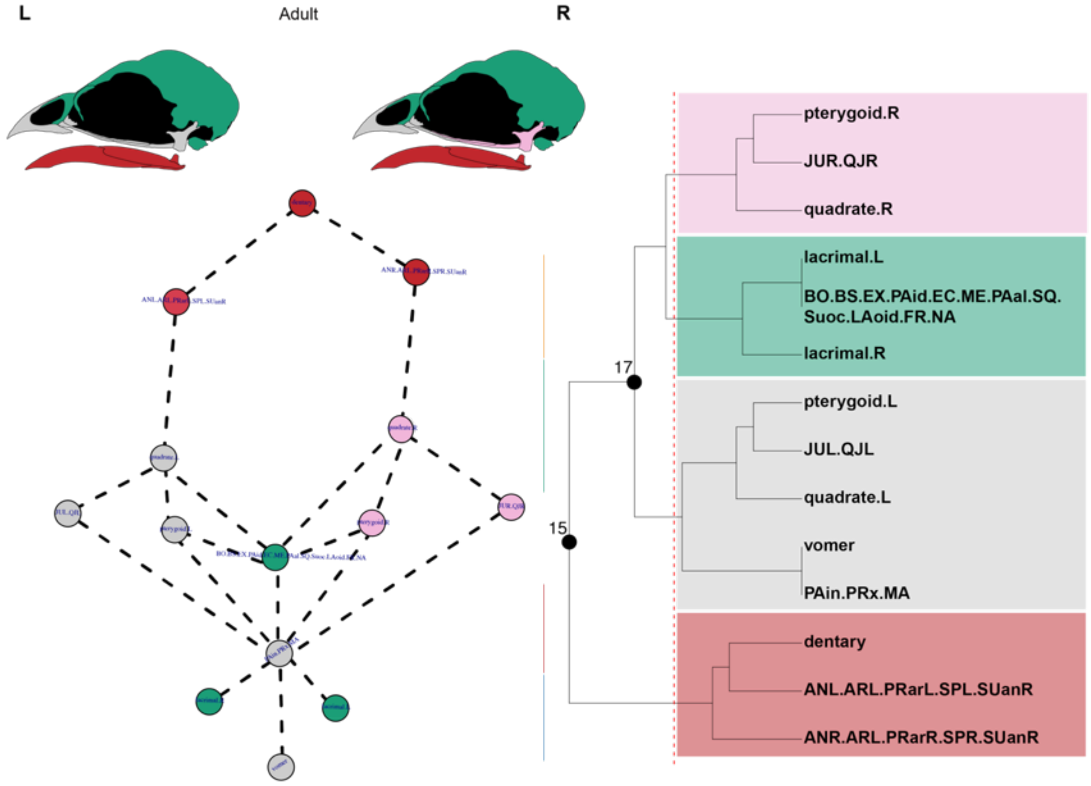

Bubulcus ibis

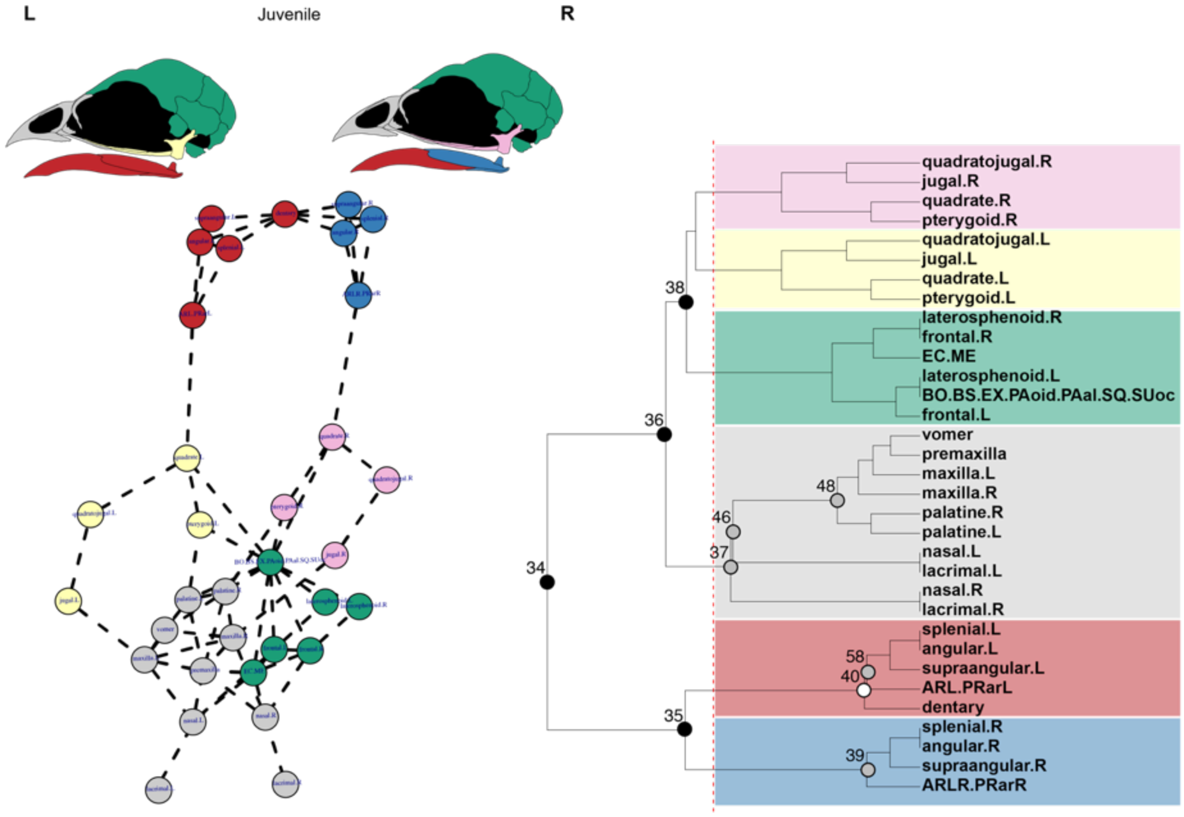

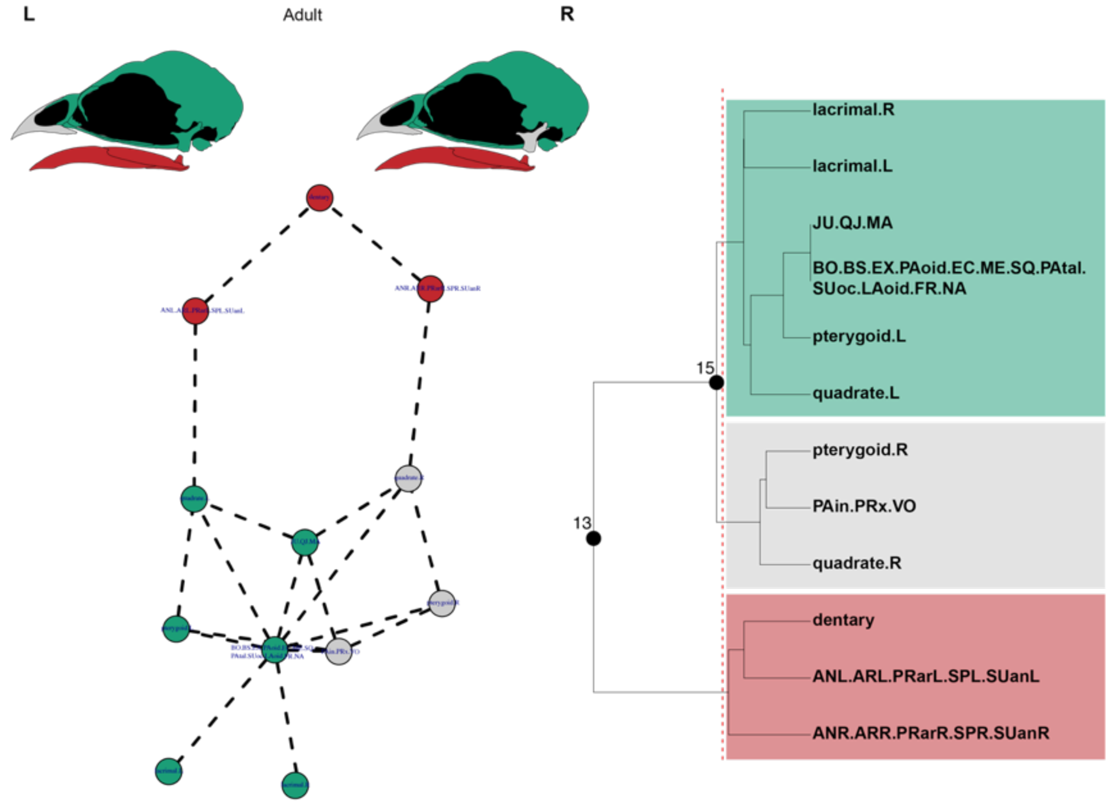

Nycticorax nycticorax

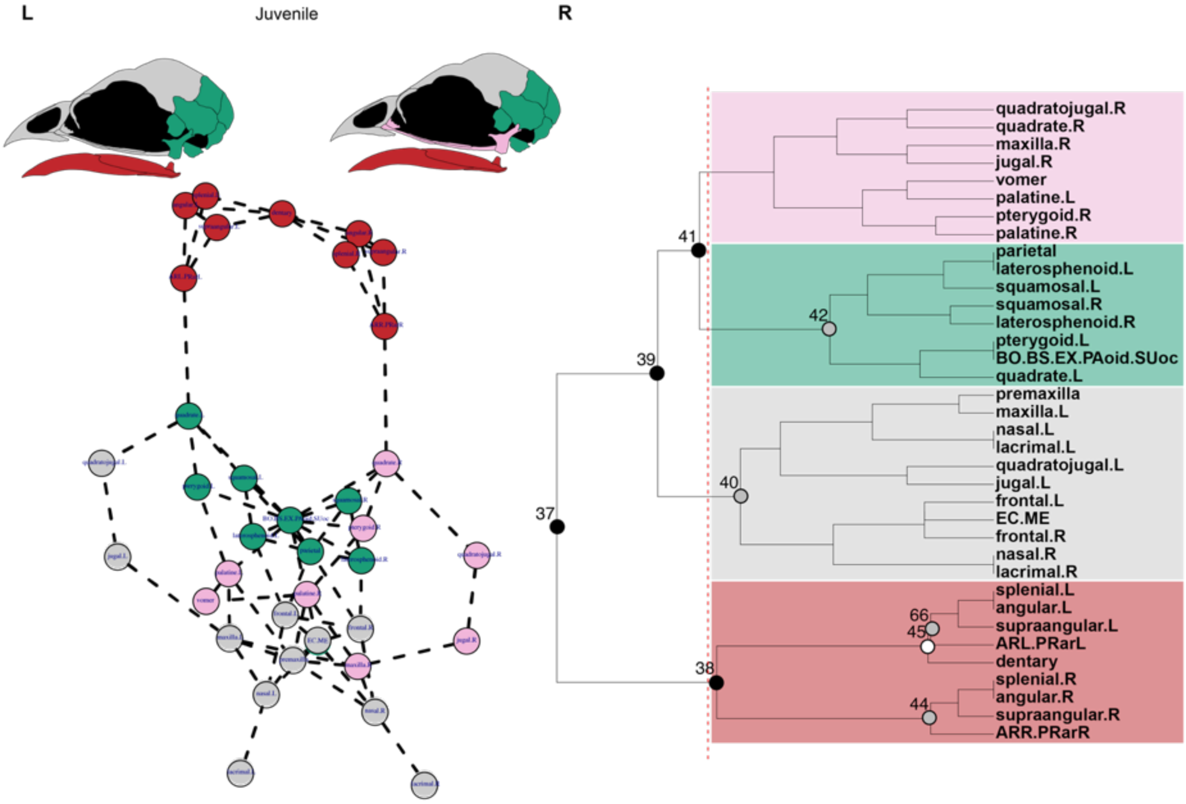

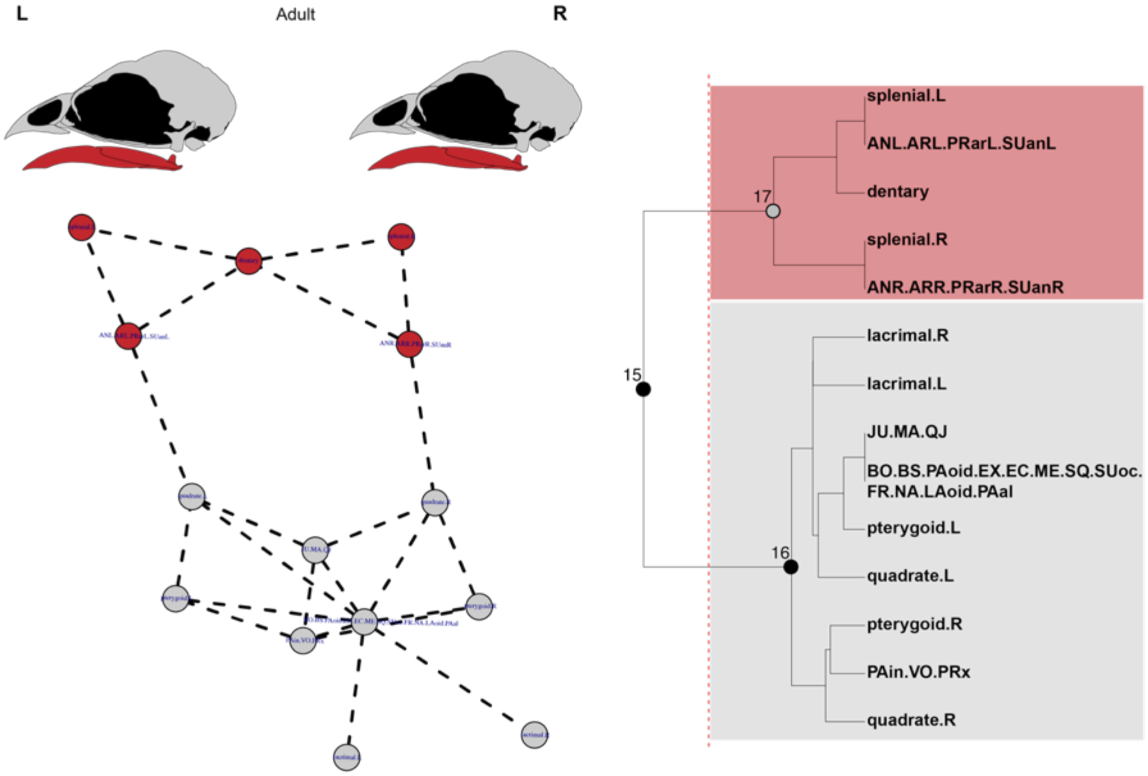

Supplement: Supplementary file 1 — Supplementary Information [file 42003_2020_914_MOESM1_ESM.pdf]
