# Supplementary material for: Birds have peramorphic skulls, too: anatomical network analyses reveal oppositional heterochronies in avian skull evolution
Source: Commun Biol. 2020 Apr 24;3:195. doi: 10.1038/s42003-020-0914-4 (PMC7181600; doi:10.1038/s42003-020-0914-4)
Supplement: Supplementary file 2 — Description of Additional Supplementary Files [file 42003_2020_914_MOESM2_ESM.pdf]

Description of Additional Supplementary

- Supplementary Data 1 (Network data)
- Supplementary Data 2 (Supplementary tables S1-S8: all source data underlying the graphs and charts in the main figures are available in the supplementary tables S1-S8).
- Supplementary Data 3 (Phylogenetic trees)
- Supplementary Data 4 (R code for anatomical network analysis)
