# Supplementary material for: Birds have peramorphic skulls, too: anatomical network analyses reveal oppositional heterochronies in avian skull evolution
Source: Commun Biol. 2020 Apr 24;3:195. doi: 10.1038/s42003-020-0914-4 (PMC7181600; doi:10.1038/s42003-020-0914-4)
Supplement: Supplementary file 7 — Reporting Summary [file 42003_2020_914_MOESM7_ESM.pdf]

nature research

Corresponding author(s): Christian Foth
Last updated by author(s): Oct 23, 2019

Reporting Summary

Nature Research wishes to improve the reproducibility of the work that we publish. This form provides structure for consistency and transparency in reporting. For further information on Nature Research policies, see Authors & Referees and the Editorial Policy Checklist.

Statistics

For all statistical analyses, confirm that the following items are present in the figure legend, table legend, main text, or Methods section.

n/a	Confirmed	
☐	☒	The exact sample size (*n*) for each experimental group/condition, given as a discrete number and unit of measurement
☐	☒	A statement on whether measurements were taken from distinct samples or whether the same sample was measured repeatedly
☐	☒	The statistical test(s) used AND whether they are one- or two-sided<br>*Only common tests should be described solely by name; describe more complex techniques in the Methods section.*
☐	☒	A description of all covariates tested
☐	☒	A description of any assumptions or corrections, such as tests of normality and adjustment for multiple comparisons
☐	☒	A full description of the statistical parameters including central tendency (e.g. means) or other basic estimates (e.g. regression coefficient) AND variation (e.g. standard deviation) or associated estimates of uncertainty (e.g. confidence intervals)
☐	☒	For null hypothesis testing, the test statistic (e.g. *F*, *t*, *r*) with confidence intervals, effect sizes, degrees of freedom and *P* value noted<br>*Give P values as exact values whenever suitable.*
☒	☐	For Bayesian analysis, information on the choice of priors and Markov chain Monte Carlo settings
☐	☒	For hierarchical and complex designs, identification of the appropriate level for tests and full reporting of outcomes
☒	☐	Estimates of effect sizes (e.g. Cohen's *d*, Pearson's *r*), indicating how they were calculated

Our web collection on statistics for biologists contains articles on many of the points above.

Software and code

Policy information about availability of computer code

Data collection	NA
Data analysis	NA

For manuscripts utilizing custom algorithms or software that are central to the research but not yet described in published literature, software must be made available to editors/reviewers. We strongly encourage code deposition in a community repository (e.g. GitHub). See the Nature Research guidelines for submitting code & software for further information.

Data

Policy information about availability of data

All manuscripts must include a data availability statement. This statement should provide the following information, where applicable:

- Accession codes, unique identifiers, or web links for publicly available datasets
- A list of figures that have associated raw data
- A description of any restrictions on data availability

All raw data and additional results are given in the extended data and supplementary information files

Field-specific reporting

Please select the one below that is the best fit for your research. If you are not sure, read the appropriate sections before making your selection.

☐ Life sciences ☐ Behavioural & social sciences ☒ Ecological, evolutionary & environmental sciences

For a reference copy of the document with all sections, see nature.com/documents/nr-reporting-summary-flat.pdf

Ecological, evolutionary & environmental sciences study design

All studies must disclose on these points even when the disclosure is negative.

Study description	We studied the skull bone configuration of juvenile and adult crown birds using Anatomical Network Analyses and compared these pattern with that of non-avian dinosaurs and Alligator.
Research sample	The sample contains information of all present bone contacts of skull bones in juvenile and adult crown birds, non-avian dinosaurs and Alligator.
Sampling strategy	The sample for extant birds based on the disposability of juvenile bird skulls in scientific collections. The sample of non-avian dinosaurs based on species with complete skulls and propper anatomical descriptions.
Data collection	The data were collected by both authors from osteological collections and from the scientific literature.
Timing and spatial scale	The data collections started in Feb 2019 and ended Jul 2019.
Data exclusions	No data were excluded from the analyses.
Reproducibility	For anatomical network analyses, the contact between all skull bones were scored in a similarity matrix. All subsequent analyses were reproducible.
Randomization	Specimens were grouped according to their taxonomic classifications. For ancestral state analyses, terminal taxa were shuffled 10,000 times, while the tree topology was held constant, and compared with the original analyses.
Blinding	Based on the type of analyses, no blinding was needed.

Did the study involve field work? ☐ Yes ☒ No

Reporting for specific materials, systems and methods

We require information from authors about some types of materials, experimental systems and methods used in many studies. Here, indicate whether each material, system or method listed is relevant to your study. If you are not sure if a list item applies to your research, read the appropriate section before selecting a response.

Materials & experimental systems

n/a	Involved in the study	
☒	☐	Antibodies
☒	☐	Eukaryotic cell lines
☐	☒	Palaeontology
☐	☒	Animals and other organisms
☒	☐	Human research participants
☒	☐	Clinical data

Methods

n/a	Involved in the study	
☒	☐	ChIP-seq
☒	☐	Flow cytometry
☒	☐	MRI-based neuroimaging

Palaeontology

Specimen provenance	All fossil data based on literature sources.
Specimen deposition	All information on species deposition are given in the relevant literature cited.
Dating methods	All dates for fossil taxa were taken from the Paleobiology Database.

☐ Tick this box to confirm that the raw and calibrated dates are available in the paper or in Supplementary Information.

Animals and other organisms

Policy information about studies involving animals; ARRIVE guidelines recommended for reporting animal research

Laboratory animals	NA
Wild animals	All extant species represent skeletal specimens that are deposited in scientific collections.
Field-collected samples	NA

Ethics oversight | NA

Note that full information on the approval of the study protocol must also be provided in the manuscript.
